# Supplementary figures and images for: Functional development of a V3/glycan-specific broadly neutralizing antibody isolated from a case of HIV superinfection
Source: eLife. 2021 Jul 15;10:e68110. doi: 10.7554/eLife.68110 (PMC8376252; doi:10.7554/eLife.68110)

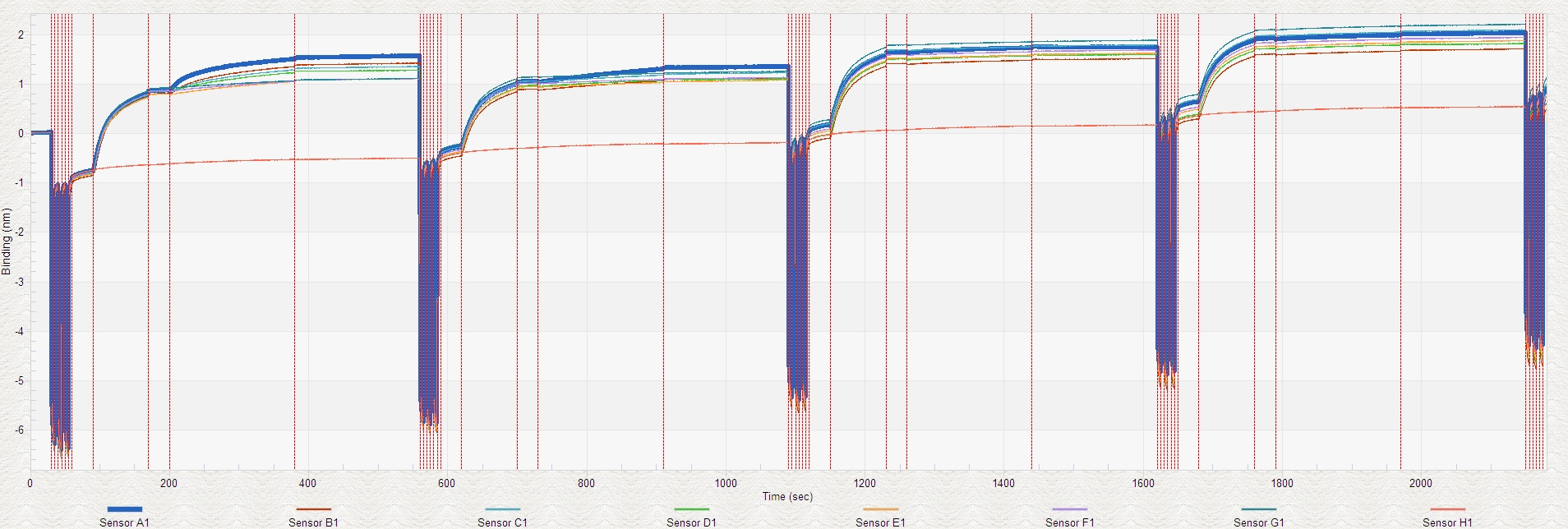

Supplement: Figure 2—source data 2. [file elife-68110-fig2-data2.zip › QA013.2 Dbl Ref BLI_2020.05.29/Mat_Gmat-L0_LatestInt_Naive_2020.05.29/Experiment_1/Misc. Files/200529_Assay_1.jpg]

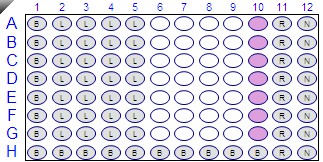

Supplement: Figure 2—source data 2. [file elife-68110-fig2-data2.zip › QA013.2 Dbl Ref BLI_2020.05.29/Mat_Gmat-L0_LatestInt_Naive_2020.05.29/Experiment_1/Misc. Files/Plate1Definition.jpg]

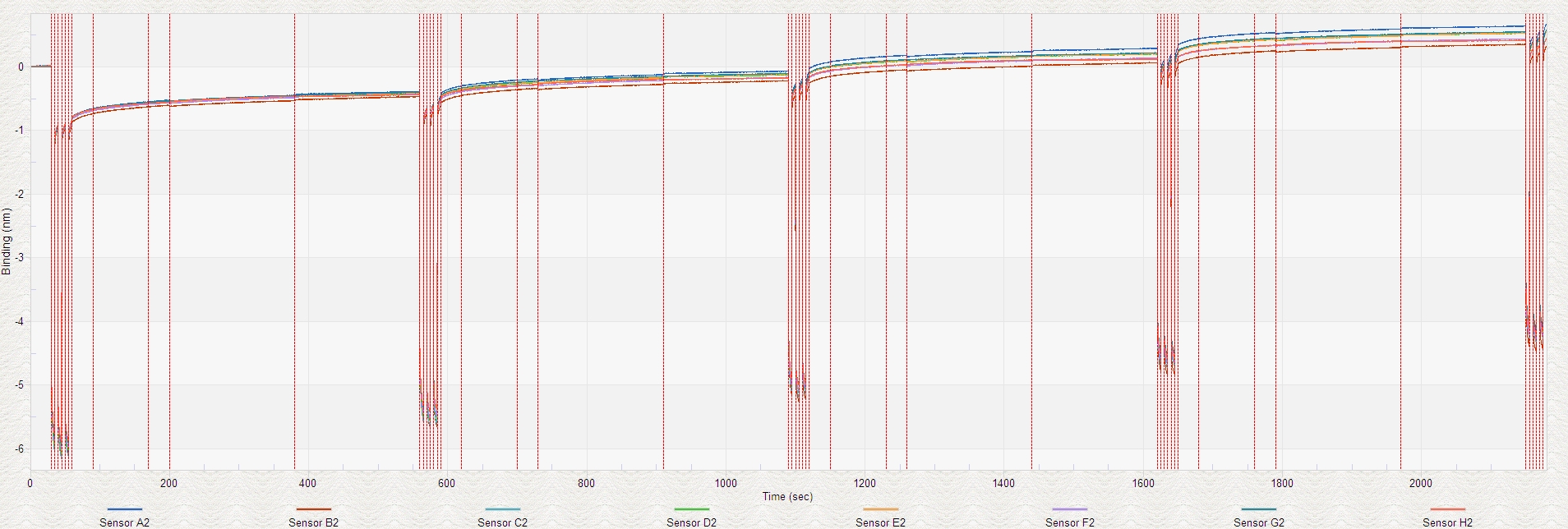

Supplement: Figure 2—source data 2. [file elife-68110-fig2-data2.zip › QA013.2 Dbl Ref BLI_2020.05.29/Mat_Gmat-L0_LatestInt_Naive_2020.05.29/Experiment_1/Misc. Files/200529_Assay_2.jpg]

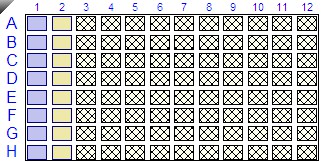

Supplement: Figure 2—source data 2. [file elife-68110-fig2-data2.zip › QA013.2 Dbl Ref BLI_2020.05.29/Mat_Gmat-L0_LatestInt_Naive_2020.05.29/Experiment_1/Misc. Files/SensorPlate.jpg]

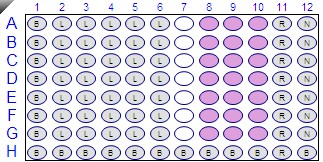

Supplement: Figure 2—source data 2. [file elife-68110-fig2-data2.zip › QA013.2 Dbl Ref_21.02.04/02.04.21/QA013.2 Lineage Replicates_21.02.04/Plate1Definition.jpg]

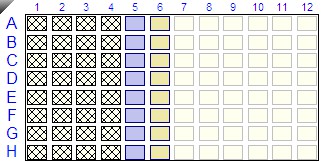

Supplement: Figure 2—source data 2. [file elife-68110-fig2-data2.zip › QA013.2 Dbl Ref_21.02.04/02.04.21/QA013.2 Lineage Replicates_21.02.04/SensorPlate.jpg]

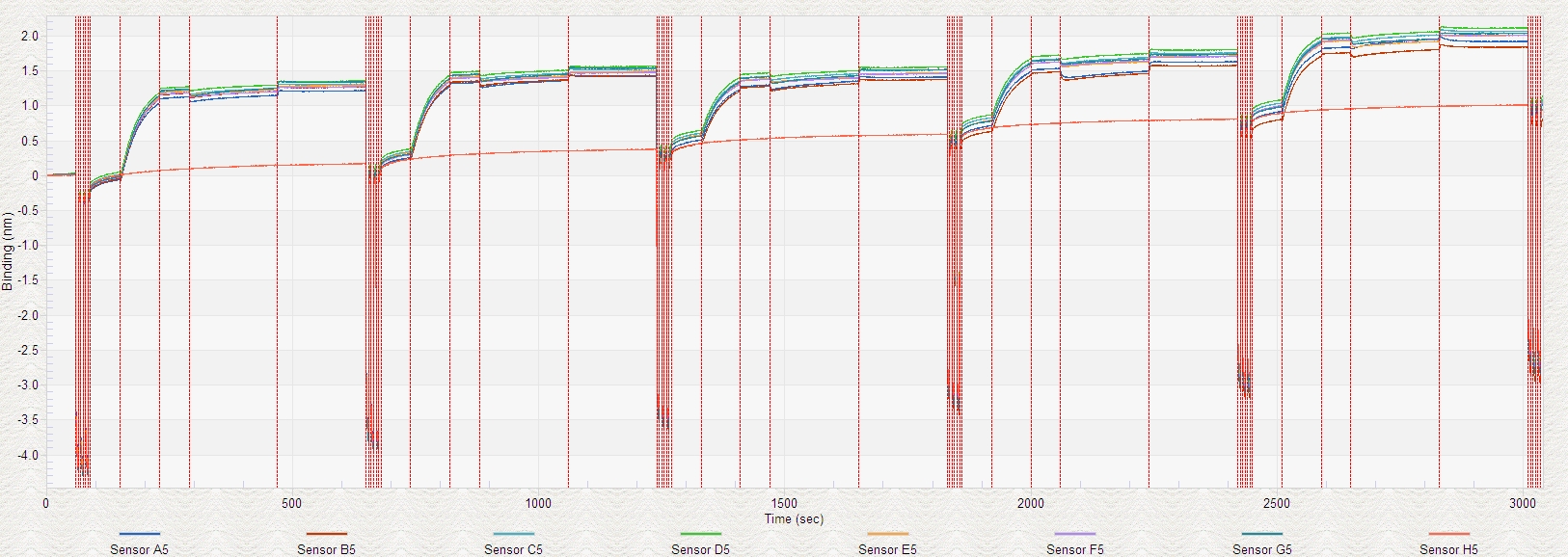

Supplement: Figure 2—source data 2. [file elife-68110-fig2-data2.zip › QA013.2 Dbl Ref_21.02.04/02.04.21/QA013.2 Lineage Replicates_21.02.04/210204_Assay_1.jpg]

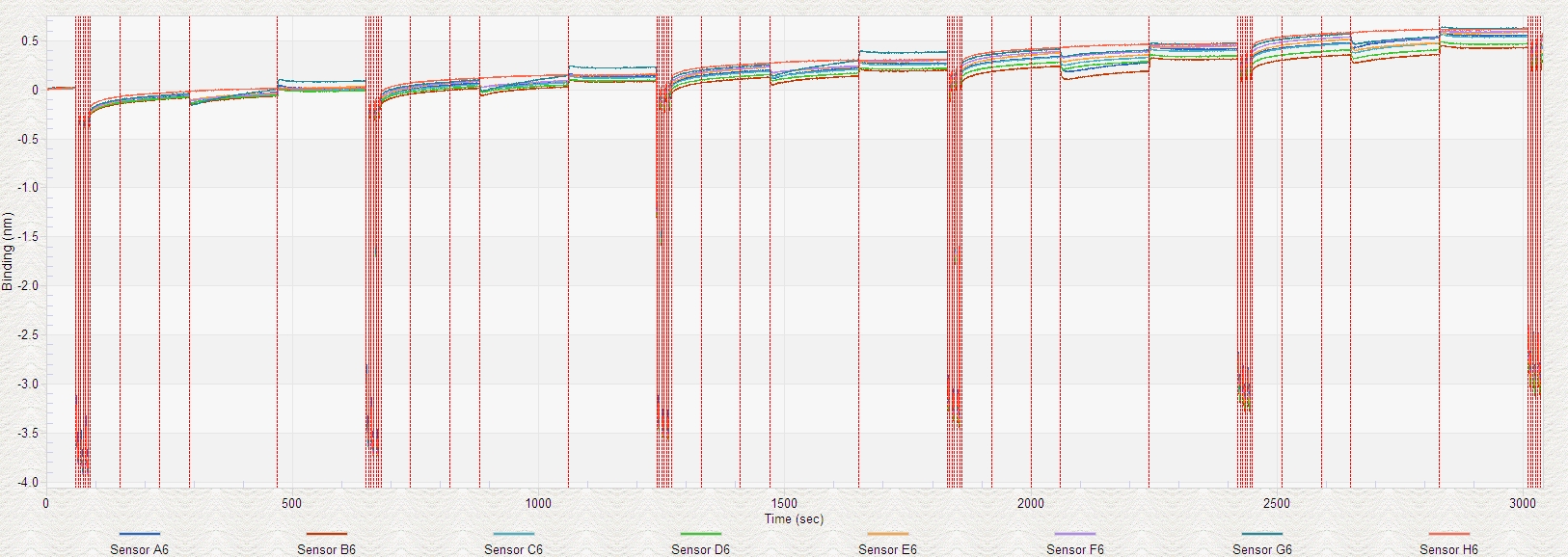

Supplement: Figure 2—source data 2. [file elife-68110-fig2-data2.zip › QA013.2 Dbl Ref_21.02.04/02.04.21/QA013.2 Lineage Replicates_21.02.04/210204_Assay_2.jpg]

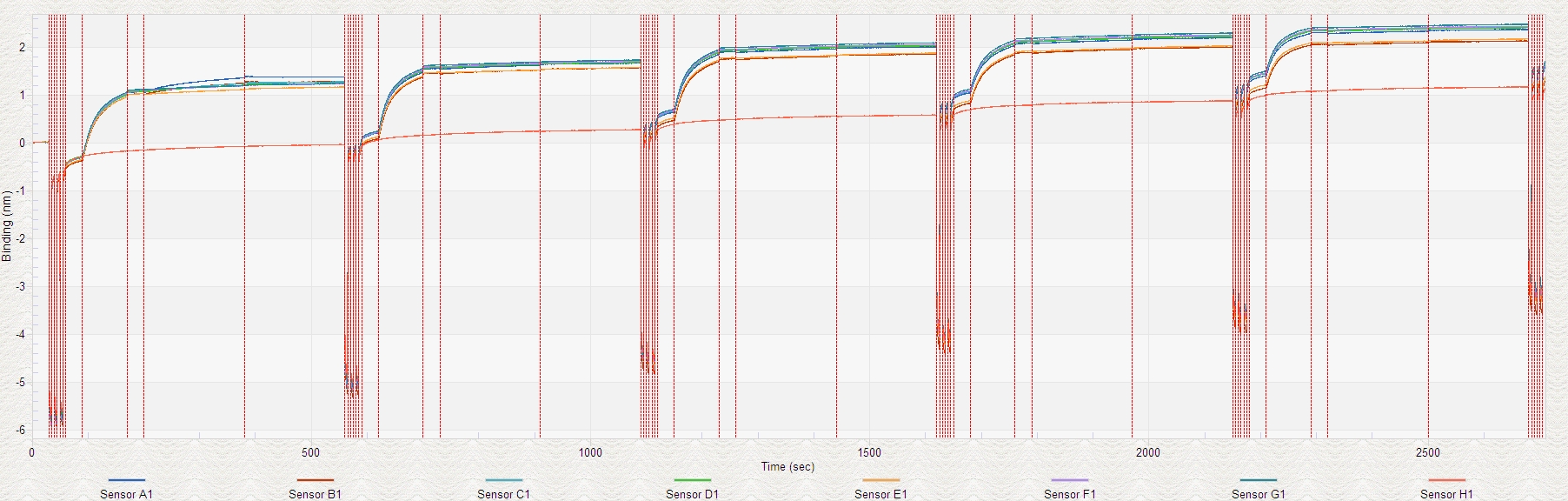

Supplement: Figure 2—source data 2. [file elife-68110-fig2-data2.zip › QA013.2 Dbl Ref BLI_2020.06.25/QA013 autologous binding_MIG_naive_G7-L4/2020.06.jpg]

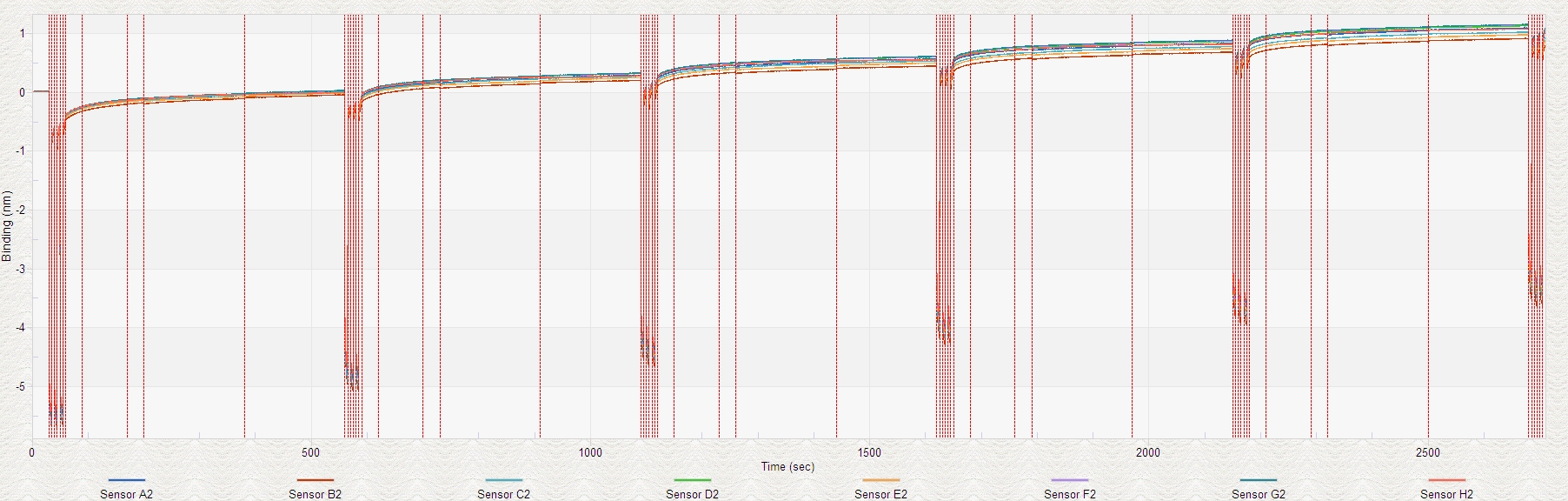

Supplement: Figure 2—source data 2. [file elife-68110-fig2-data2.zip › QA013.2 Dbl Ref BLI_2020.06.25/QA013 autologous binding_MIG_naive_G7-L4/2020.06.25_Assay_2_2.jpg]

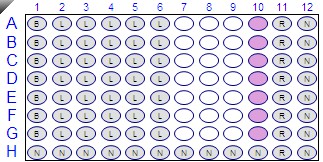

Supplement: Figure 2—source data 2. [file elife-68110-fig2-data2.zip › QA013.2 Dbl Ref BLI_2020.06.25/QA013 autologous binding_MIG_naive_G7-L4/Plate1Definition.jpg]

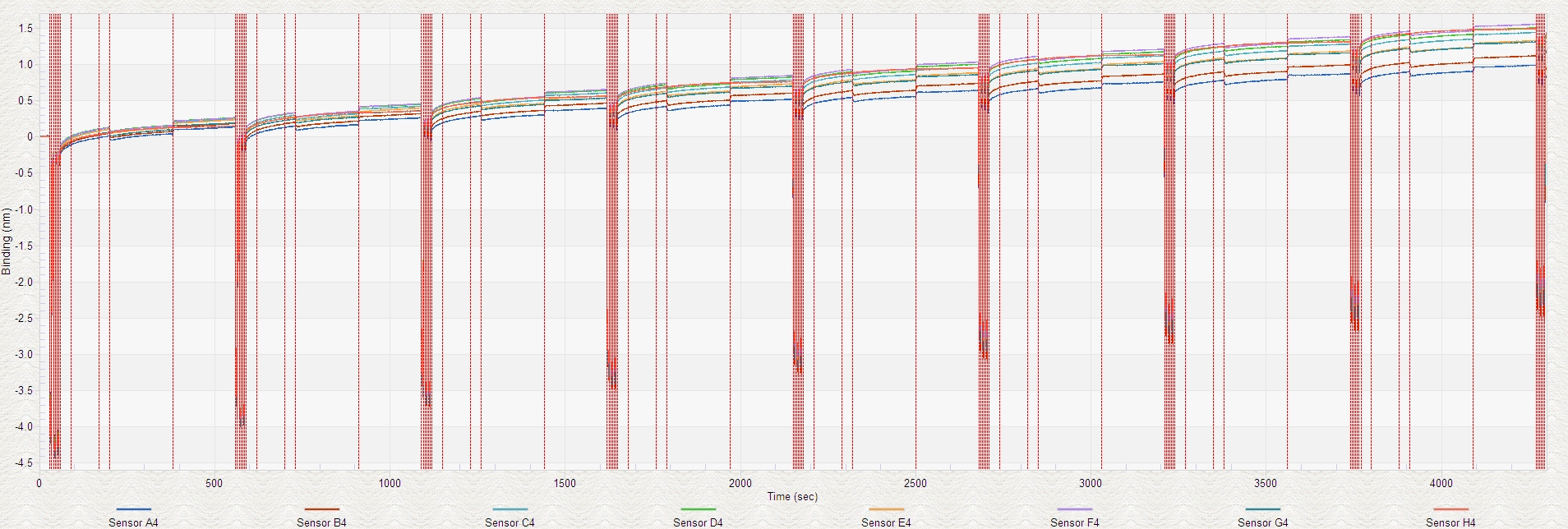

Supplement: Figure 4—source data 2. [file elife-68110-fig4-data2.zip › QA013.2 Dbl Ref_01.28.21/QA013.2_Lineage Replicates_BG505 SOSIP trimer/210128_Assay_2.jpg]

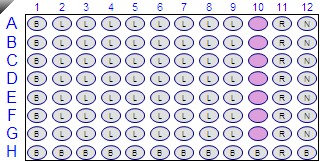

Supplement: Figure 4—source data 2. [file elife-68110-fig4-data2.zip › QA013.2 Dbl Ref_01.28.21/QA013.2_Lineage Replicates_BG505 SOSIP trimer/Plate1Definition.jpg]

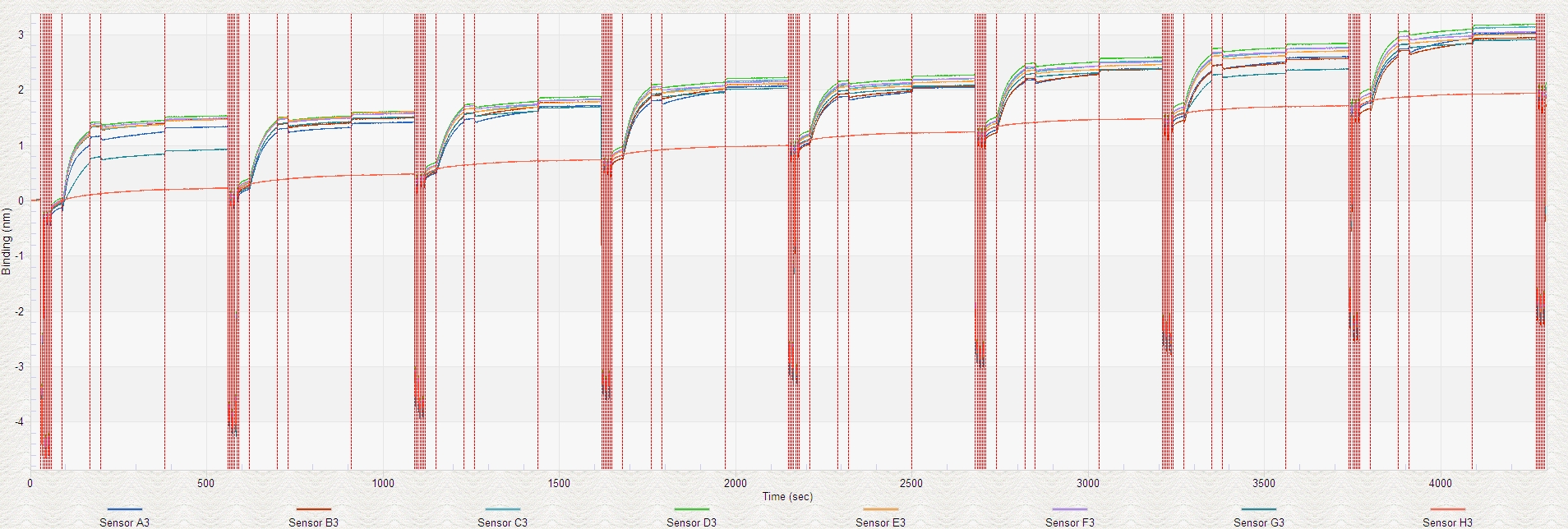

Supplement: Figure 4—source data 2. [file elife-68110-fig4-data2.zip › QA013.2 Dbl Ref_01.28.21/QA013.2_Lineage Replicates_BG505 SOSIP trimer/210128_Assay_1.jpg]

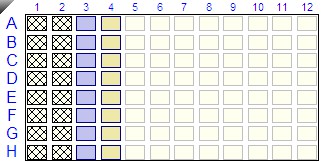

Supplement: Figure 4—source data 2. [file elife-68110-fig4-data2.zip › QA013.2 Dbl Ref_01.28.21/QA013.2_Lineage Replicates_BG505 SOSIP trimer/SensorPlate.jpg]
