# Supplementary material for: Functional development of a V3/glycan-specific broadly neutralizing antibody isolated from a case of HIV superinfection
Source: eLife. 2021 Jul 15;10:e68110. doi: 10.7554/eLife.68110 (PMC8376252; doi:10.7554/eLife.68110)
Supplement: Figure 3—source data 2. [file elife-68110-fig3-data2.zip › eLife 04-03-2021-RA-eLife-68110_cryo-EM data/D_1000255470_val-report-full_P1.pdf]

Preliminary Full wwPDB EM Validation Report (i)

Mar 13, 2021 – 05:04 PM EST

Deposition ID : D_1000255470

This is a Preliminary Full wwPDB EM Validation Report.

This report is produced by the wwPDB Deposition System during initial deposition but before annotation of the structure.

We welcome your comments at *validation@mail.wwpdb.org*
A user guide is available at
https://www.wwpdb.org/validation/2017/EMValidationReportHelp
with specific help available everywhere you see the (i) symbol.

PRELIMINARY VALIDATION REPORT

The following versions of software and data (see references (i)) were used in the production of this report:

EMDB validation analysis	:	0.0.0.dev61
Mogul	:	1.8.5 (274361), CSD as541be (2020)
MolProbity	:	4.02b-467
Percentile statistics	:	20191225.v01 (using entries in the PDB archive December 25th 2019)
Ideal geometry (proteins)	:	Engh & Huber (2001)
Ideal geometry (DNA, RNA)	:	Parkinson et al. (1996)
Validation Pipeline (wwPDB-VP)	:	2.17.1

1 Overall quality at a glance (i)

The following experimental techniques were used to determine the structure:
ELECTRON MICROSCOPY

The reported resolution of this entry is unknown.

Percentile scores (ranging between 0-100) for global validation metrics of the entry are shown in the following graphic. The table shows the number of entries on which the scores are based.

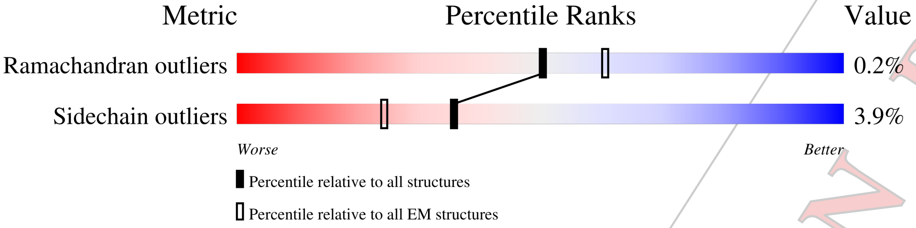

Metric	Whole archive (#Entries)	EM structures (#Entries)
Ramachandran outliers	154571	4023
Sidechain outliers	154315	3826

The table below summarises the geometric issues observed across the polymeric chains and their fit to the map. The red, orange, yellow and green segments of the bar indicate the fraction of residues that contain outliers for >=3, 2, 1 and 0 types of geometric quality criteria respectively. A grey segment represents the fraction of residues that are not modelled. The numeric value for each fraction is indicated below the corresponding segment, with a dot representing fractions <=5% The upper red bar (where present) indicates the fraction of residues that have poor fit to the EM map (all-atom inclusion < 40%). The numeric value is given above the bar.

Mol	Chain	Length	Quality of chain
1	A	481	• / 89% • 6%
1	E	481	• / 89% • 6%
1	I	481	• / 89% • 6%
2	B	153	• / 83% • 13%
2	F	153	• / 83% • 13%
2	J	153	• / 84% • 13%
3	C	128	• / 98% •
3	G	128	• / 98% •
3	K	128	• / 98% •

Continued on next page...

PRELIMINARY VALIDATION REPORT

Continued from previous page...

Mol	Chain	Length	Quality of chain
4	D	108	95%
4	H	108	95%
4	L	108	95%
5	1	3	33% 100%
5	6	3	33% 67%
5	7	3	33% 67%
5	M	3	67% 33% 67%
5	O	3	100%
5	P	3	33% 33% 67%
5	T	3	33% 100%
5	Y	3	33% 33% 67%
5	Z	3	33% 67%
5	d	3	67% 33% 67%
5	f	3	100%
5	g	3	33% 33% 67%
5	k	3	100%
5	p	3	33% 33% 67%
5	q	3	33% 67%
5	u	3	67% 67% 33%
5	w	3	100%
5	x	3	33% 33% 67%
6	5	2	50% 50% 50%
6	8	2	100%
6	9	2	100%
6	AA	2	50% 100%

Continued on next page...

PRELIMINARY VALIDATION REPORT

Continued from previous page...

Mol	Chain	Length	Quality of chain
6	N	2	100%
6	Q	2	50% 50%
6	R	2	100%
6	X	2	100%<br>50% 50%
6	a	2	100%
6	b	2	100%
6	c	2	50%<br>100%
6	e	2	100%
6	h	2	50% 50%
6	i	2	50% 50%
6	o	2	100%<br>50% 50%
6	r	2	100%
6	s	2	100%
6	t	2	50%<br>100%
6	v	2	100%
6	y	2	50% 50%
6	z	2	50%<br>50% 50%
7	0	7	14%<br>100%
7	S	7	14%<br>100%
7	j	7	14%<br>100%
8	2	5	60% 40%
8	U	5	60% 40%
8	l	5	60% 40%
9	3	7	100%
9	V	7	100%

Continued on next page...

PRELIMINARY VALIDATION REPORT

Continued from previous page...

Mol	Chain	Length	Quality of chain
9	m	7	100%
10	4	9	11% 100%
10	W	9	11% 100%
10	n	9	11% 100%

PRELIMINARY VALIDATION REPORT

2 Entry composition (i)

There are 12 unique types of molecules in this entry. The entry contains 21564 atoms, of which 0 are hydrogens and 0 are deuteriums.

In the tables below, the AltConf column contains the number of residues with at least one atom in alternate conformation and the Trace column contains the number of residues modelled with at most 2 atoms.

- Molecule 1 is a protein.

Mol	Chain	Residues	Atoms					AltConf	Trace
1	A	450	Total 3524	C 2215	N 623	O 658	S 28	0	0
1	E	450	Total 3524	C 2215	N 623	O 658	S 28	0	0
1	I	450	Total 3524	C 2215	N 623	O 658	S 28	0	0

- Molecule 2 is a protein.

Mol	Chain	Residues	Atoms					AltConf	Trace
2	B	133	Total 1056	C 670	N 181	O 199	S 6	0	0
2	F	133	Total 1056	C 670	N 181	O 199	S 6	0	0
2	J	133	Total 1056	C 670	N 181	O 199	S 6	0	0

- Molecule 3 is a protein.

Mol	Chain	Residues	Atoms					AltConf	Trace
3	C	128	Total 1015	C 640	N 180	O 192	S 3	0	0
3	G	128	Total 1015	C 640	N 180	O 192	S 3	0	0
3	K	128	Total 1015	C 640	N 180	O 192	S 3	0	0

- Molecule 4 is a protein.

Mol	Chain	Residues	Atoms					AltConf	Trace
4	D	107	Total 778	C 480	N 136	O 160	S 2	0	0

Continued on next page...

PRELIMINARY VALIDATION REPORT

Continued from previous page...

Mol	Chain	Residues	Atoms					AltConf	Trace
4	H	107	Total 778	C 480	N 136	O 160	S 2	0	0
4	L	107	Total 778	C 480	N 136	O 160	S 2	0	0

- Molecule 5 is an oligosaccharide called beta-D-mannopyranose-(1-4)-2-acetamido-2-deoxy-beta-D-glucopyranose-(1-4)-2-acetamido-2-deoxy-beta-D-glucopyranose.

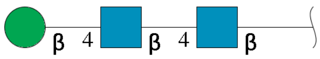

Mol	Chain	Residues	Atoms				AltConf	Trace
5	M	3	Total 39	C 22	N 2	O 15	0	0
5	O	3	Total 39	C 22	N 2	O 15	0	0
5	P	3	Total 39	C 22	N 2	O 15	0	0
5	T	3	Total 39	C 22	N 2	O 15	0	0
5	Y	3	Total 39	C 22	N 2	O 15	0	0
5	Z	3	Total 39	C 22	N 2	O 15	0	0
5	d	3	Total 39	C 22	N 2	O 15	0	0
5	f	3	Total 39	C 22	N 2	O 15	0	0
5	g	3	Total 39	C 22	N 2	O 15	0	0
5	k	3	Total 39	C 22	N 2	O 15	0	0
5	p	3	Total 39	C 22	N 2	O 15	0	0
5	q	3	Total 39	C 22	N 2	O 15	0	0
5	u	3	Total 39	C 22	N 2	O 15	0	0
5	w	3	Total 39	C 22	N 2	O 15	0	0
5	x	3	Total 39	C 22	N 2	O 15	0	0

Continued on next page...

PRELIMINARY VALIDATION REPORT

Continued from previous page...

Mol	Chain	Residues	Atoms	AltConf	Trace
5	1	3	Total 39 C 22 N 2 O 15	0	0
5	6	3	Total 39 C 22 N 2 O 15	0	0
5	7	3	Total 39 C 22 N 2 O 15	0	0

- Molecule 6 is an oligosaccharide called 2-acetamido-2-deoxy-beta-D-glucopyranose-(1-4)-2-acetamido-2-deoxy-beta-D-glucopyranose.

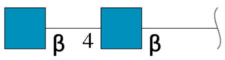

Mol	Chain	Residues	Atoms	AltConf	Trace
6	N	2	Total 28 C 16 N 2 O 10	0	0
6	Q	2	Total 28 C 16 N 2 O 10	0	0
6	R	2	Total 28 C 16 N 2 O 10	0	0
6	X	2	Total 28 C 16 N 2 O 10	0	0
6	a	2	Total 28 C 16 N 2 O 10	0	0
6	b	2	Total 28 C 16 N 2 O 10	0	0
6	c	2	Total 28 C 16 N 2 O 10	0	0
6	e	2	Total 28 C 16 N 2 O 10	0	0
6	h	2	Total 28 C 16 N 2 O 10	0	0
6	i	2	Total 28 C 16 N 2 O 10	0	0
6	o	2	Total 28 C 16 N 2 O 10	0	0
6	r	2	Total 28 C 16 N 2 O 10	0	0
6	s	2	Total 28 C 16 N 2 O 10	0	0
6	t	2	Total 28 C 16 N 2 O 10	0	0

Continued on next page...

PRELIMINARY VALIDATION REPORT

Continued from previous page...

Mol	Chain	Residues	Atoms	AltConf	Trace
6	v	2	Total C N O<br>28 16 2 10	0	0
6	y	2	Total C N O<br>28 16 2 10	0	0
6	z	2	Total C N O<br>28 16 2 10	0	0
6	5	2	Total C N O<br>28 16 2 10	0	0
6	8	2	Total C N O<br>28 16 2 10	0	0
6	9	2	Total C N O<br>28 16 2 10	0	0
6	AA	2	Total C N O<br>28 16 2 10	0	0

- Molecule 7 is an oligosaccharide called alpha-D-mannopyranose-(1-2)-alpha-D-mannopyranose-(1-3)-[alpha-D-mannopyranose-(1-3)-alpha-D-mannopyranose-(1-6)]beta-D-mannopyranose-(1-4)-2-acetamido-2-deoxy-beta-D-glucopyranose-(1-4)-2-acetamido-2-deoxy-beta-D-glucopyranose.

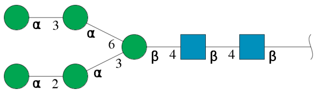

Mol	Chain	Residues	Atoms	AltConf	Trace
7	S	7	Total C N O<br>83 46 2 35	0	0
7	j	7	Total C N O<br>83 46 2 35	0	0
7	0	7	Total C N O<br>83 46 2 35	0	0

- Molecule 8 is an oligosaccharide called alpha-D-mannopyranose-(1-3)-[alpha-D-mannopyranose-(1-6)]beta-D-mannopyranose-(1-4)-2-acetamido-2-deoxy-beta-D-glucopyranose-(1-4)-2-acetamido-2-deoxy-beta-D-glucopyranose.

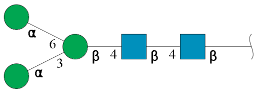

PRELIMINARY VALIDATION REPORT

Mol	Chain	Residues	Atoms				AltConf	Trace
8	U	5	Total 61	C 34	N 2	O 25	0	0
8	l	5	Total 61	C 34	N 2	O 25	0	0
8	2	5	Total 61	C 34	N 2	O 25	0	0

- Molecule 9 is an oligosaccharide called alpha-D-mannopyranose-(1-3)-[alpha-D-mannopyranose-(1-6)]alpha-D-mannopyranose-(1-6)-[alpha-D-mannopyranose-(1-3)]beta-D-mannopyranose-(1-4)-2-acetamido-2-deoxy-beta-D-glucopyranose-(1-4)-2-acetamido-2-deoxy-beta-D-glucopyranose.

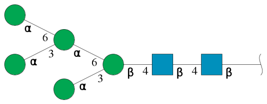

Mol	Chain	Residues	Atoms				AltConf	Trace
9	V	7	Total 83	C 46	N 2	O 35	0	0
9	m	7	Total 83	C 46	N 2	O 35	0	0
9	3	7	Total 83	C 46	N 2	O 35	0	0

- Molecule 10 is an oligosaccharide called alpha-D-mannopyranose-(1-2)-alpha-D-mannopyranose-(1-6)-[alpha-D-mannopyranose-(1-3)]alpha-D-mannopyranose-(1-6)-[alpha-D-mannopyranose-(1-2)-alpha-D-mannopyranose-(1-3)]beta-D-mannopyranose-(1-4)-2-acetamido-2-deoxy-beta-D-glucopyranose-(1-4)-2-acetamido-2-deoxy-beta-D-glucopyranose.

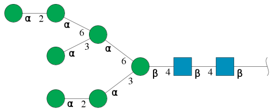

Mol	Chain	Residues	Atoms				AltConf	Trace
10	W	9	Total 105	C 58	N 2	O 45	0	0
10	n	9	Total 105	C 58	N 2	O 45	0	0
10	4	9	Total 105	C 58	N 2	O 45	0	0

- Molecule 11 is alpha-D-mannopyranose (three-letter code: MAN) (formula: $C_6H_{12}O_6$).

PRELIMINARY VALIDATION REPORT

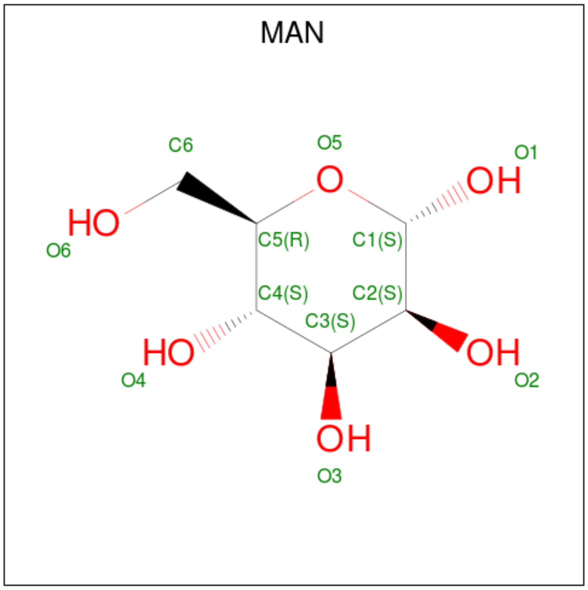

Mol	Chain	Residues	Atoms	AltConf
11	A	1	Total C O<br>11 6 5	0
11	E	1	Total C O<br>11 6 5	0
11	I	1	Total C O<br>11 6 5	0

- Molecule 12 is 2-acetamido-2-deoxy-beta-D-glucopyranose (three-letter code: NAG) (formula: $C_8H_{15}NO_6$).

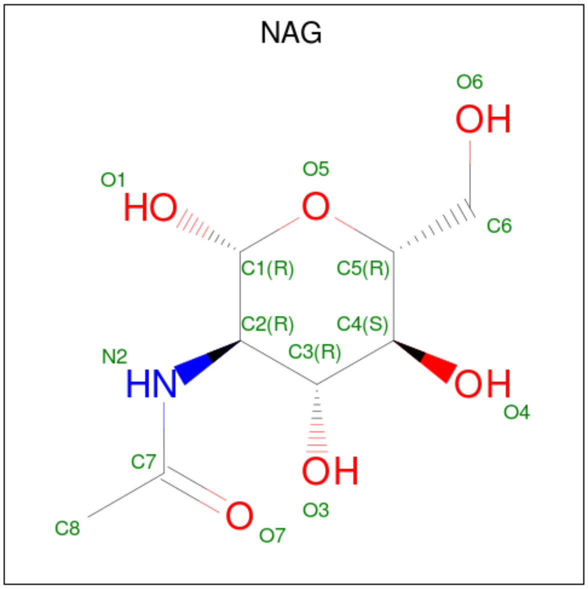

PRELIMINARY VALIDATION REPORT

Mol	Chain	Residues	Atoms				AltConf
12	A	1	Total 14	C 8	N 1	O 5	0
12	B	1	Total 28	C 16	N 2	O 10	0
12	B	1	Total 28	C 16	N 2	O 10	0
12	E	1	Total 14	C 8	N 1	O 5	0
12	F	1	Total 28	C 16	N 2	O 10	0
12	F	1	Total 28	C 16	N 2	O 10	0
12	I	1	Total 14	C 8	N 1	O 5	0
12	J	1	Total 28	C 16	N 2	O 10	0
12	J	1	Total 28	C 16	N 2	O 10	0

PRELIMINARY VALIDATION REPORT

3 Residue-property plots (i)

These plots are drawn for all protein, RNA, DNA and oligosaccharide chains in the entry. The first graphic for a chain summarises the proportions of the various outlier classes displayed in the second graphic. The second graphic shows the sequence view annotated by issues in geometry and atom inclusion in map density. Residues are color-coded according to the number of geometric quality criteria for which they contain at least one outlier: green = 0, yellow = 1, orange = 2 and red = 3 or more. A red diamond above a residue indicates a poor fit to the EM map for this residue (all-atom inclusion < 40%). Stretches of 2 or more consecutive residues without any outlier are shown as a green connector. Residues present in the sample, but not in the model, are shown in grey.

- Molecule 1:

Chain A:

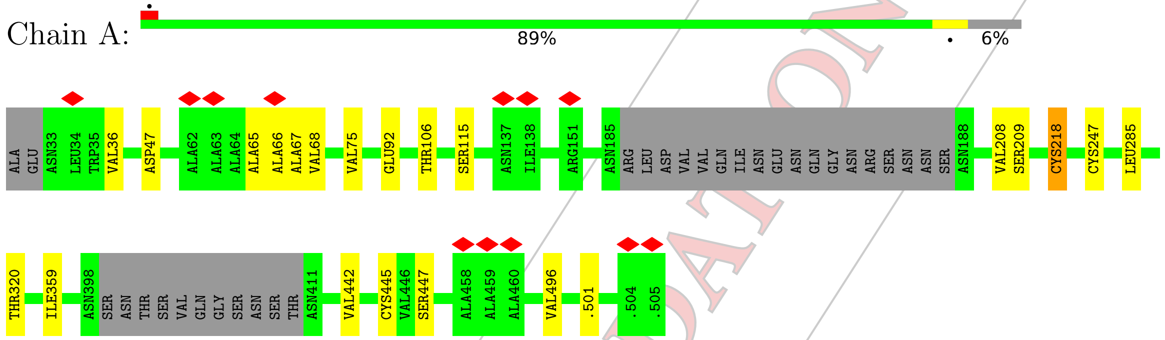

- Molecule 1:

Chain E:

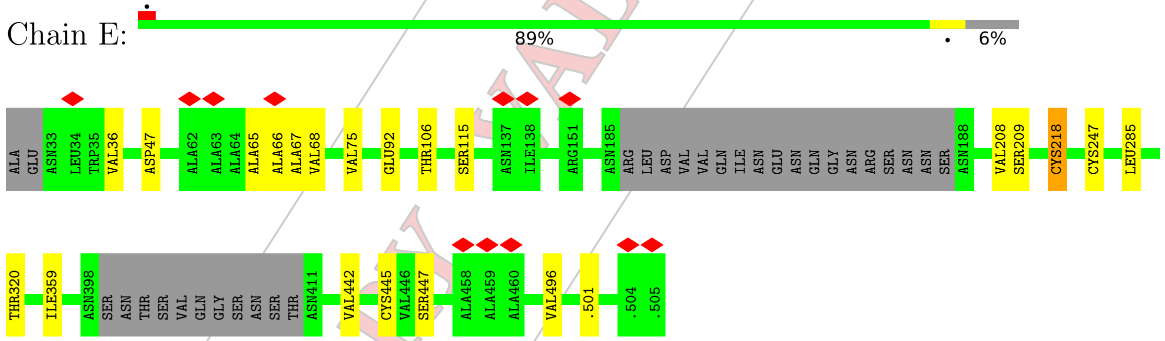

- Molecule 1:

Chain I:

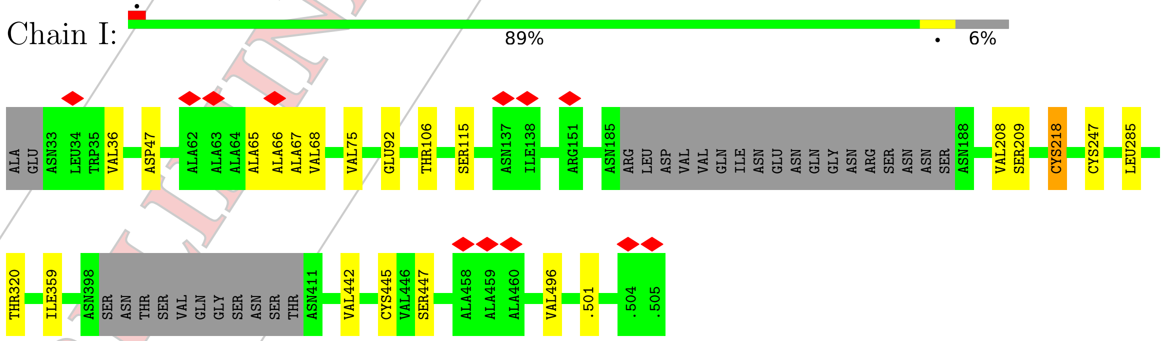

- Molecule 2:

PRELIMINARY VALIDATION REPORT

Chain B: 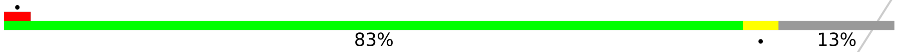

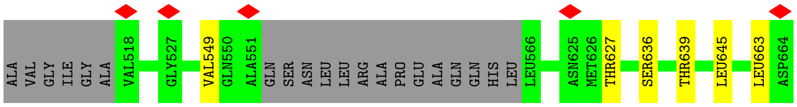

• Molecule 2:

Chain F: 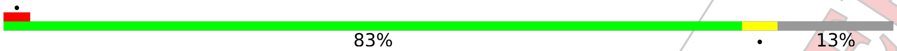

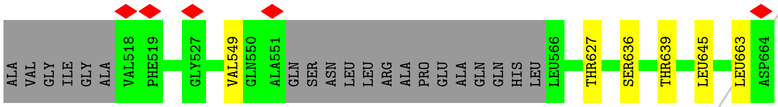

• Molecule 2:

Chain J: 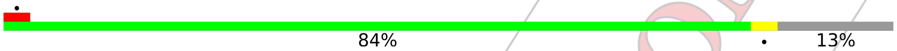

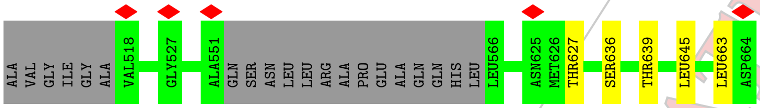

• Molecule 3:

Chain C: 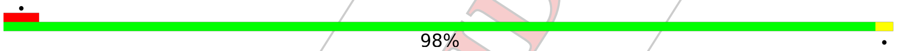

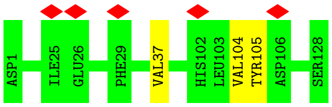

• Molecule 3:

Chain G: 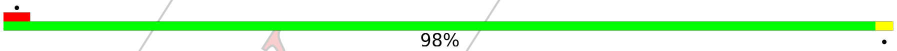

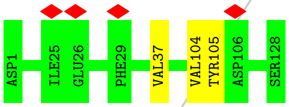

• Molecule 3:

Chain K: 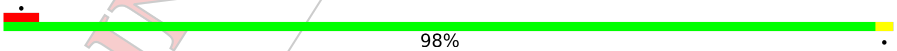

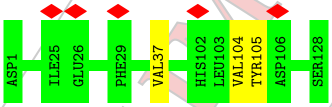

• Molecule 4:

Chain D: 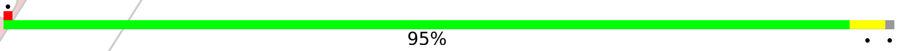

PRELIMINARY VALIDATION REPORT

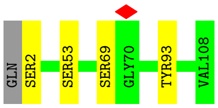

• Molecule 4:

Chain H: 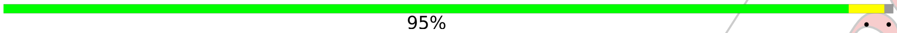

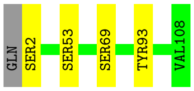

• Molecule 4:

Chain L: 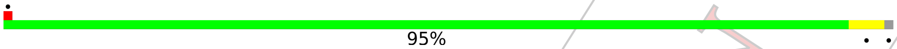

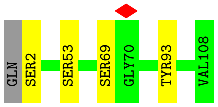

• Molecule 5: beta-D-mannopyranose-(1-4)-2-acetamido-2-deoxy-beta-D-glucopyranose-(1-4)-2-acetamido-2-deoxy-beta-D-glucopyranose

Chain M: 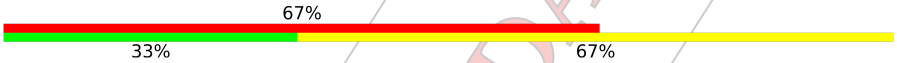

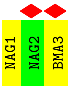

• Molecule 5: beta-D-mannopyranose-(1-4)-2-acetamido-2-deoxy-beta-D-glucopyranose-(1-4)-2-acetamido-2-deoxy-beta-D-glucopyranose

Chain O: 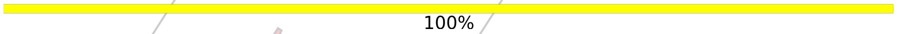

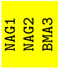

• Molecule 5: beta-D-mannopyranose-(1-4)-2-acetamido-2-deoxy-beta-D-glucopyranose-(1-4)-2-acetamido-2-deoxy-beta-D-glucopyranose

Chain P: 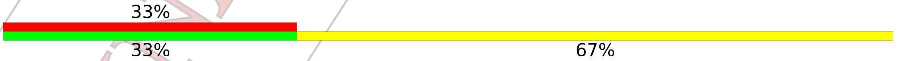

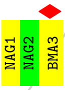

• Molecule 5: beta-D-mannopyranose-(1-4)-2-acetamido-2-deoxy-beta-D-glucopyranose-(1-4)-2-acetamido-2-deoxy-beta-D-glucopyranose

Chain T: 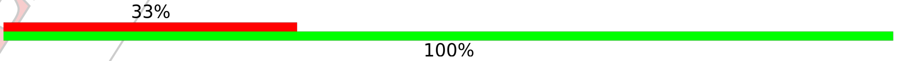

PRELIMINARY VALIDATION REPORT

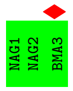

• Molecule 5: beta-D-mannopyranose-(1-4)-2-acetamido-2-deoxy-beta-D-glucopyranose-(1-4)-2-acetamido-2-deoxy-beta-D-glucopyranose

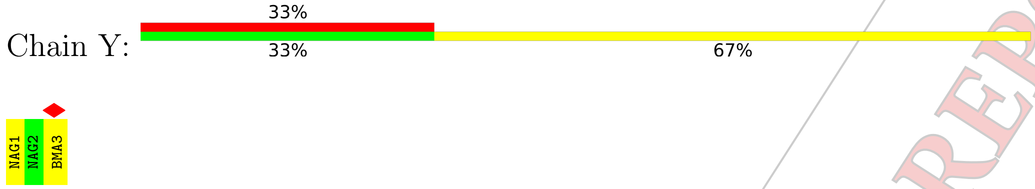

• Molecule 5: beta-D-mannopyranose-(1-4)-2-acetamido-2-deoxy-beta-D-glucopyranose-(1-4)-2-acetamido-2-deoxy-beta-D-glucopyranose

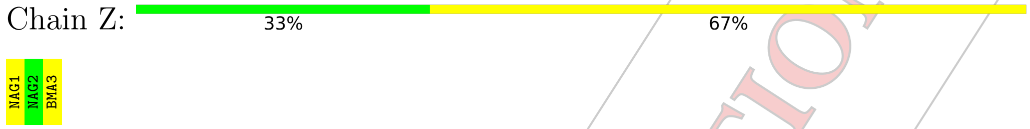

• Molecule 5: beta-D-mannopyranose-(1-4)-2-acetamido-2-deoxy-beta-D-glucopyranose-(1-4)-2-acetamido-2-deoxy-beta-D-glucopyranose

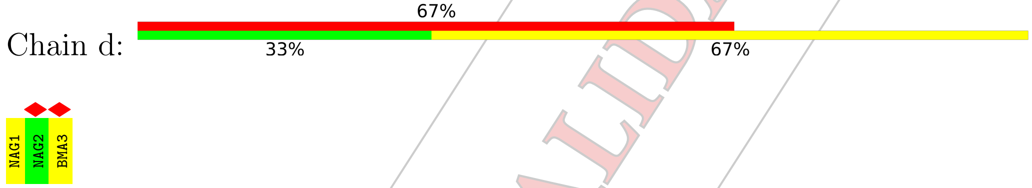

• Molecule 5: beta-D-mannopyranose-(1-4)-2-acetamido-2-deoxy-beta-D-glucopyranose-(1-4)-2-acetamido-2-deoxy-beta-D-glucopyranose

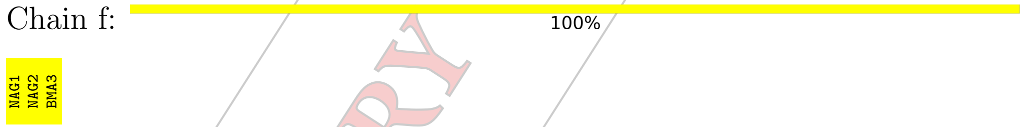

• Molecule 5: beta-D-mannopyranose-(1-4)-2-acetamido-2-deoxy-beta-D-glucopyranose-(1-4)-2-acetamido-2-deoxy-beta-D-glucopyranose

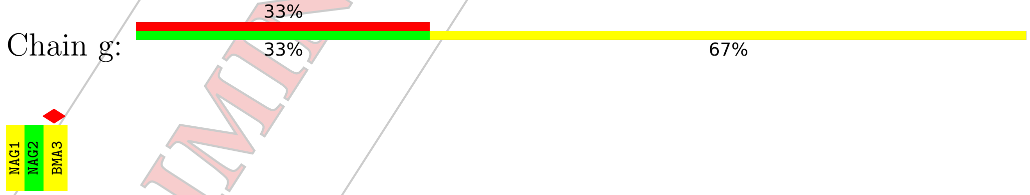

• Molecule 5: beta-D-mannopyranose-(1-4)-2-acetamido-2-deoxy-beta-D-glucopyranose-(1-4)-2-acetamido-2-deoxy-beta-D-glucopyranose

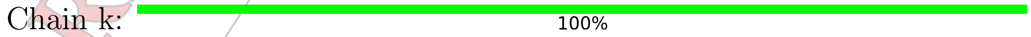

PRELIMINARY VALIDATION REPORT

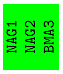

• Molecule 5: beta-D-mannopyranose-(1-4)-2-acetamido-2-deoxy-beta-D-glucopyranose-(1-4)-2-acetamido-2-deoxy-beta-D-glucopyranose

Chain p:

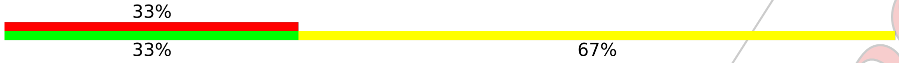

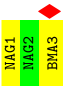

• Molecule 5: beta-D-mannopyranose-(1-4)-2-acetamido-2-deoxy-beta-D-glucopyranose-(1-4)-2-acetamido-2-deoxy-beta-D-glucopyranose

Chain q:

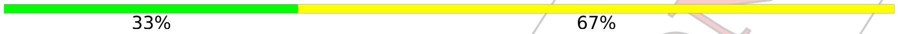

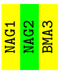

• Molecule 5: beta-D-mannopyranose-(1-4)-2-acetamido-2-deoxy-beta-D-glucopyranose-(1-4)-2-acetamido-2-deoxy-beta-D-glucopyranose

Chain u:

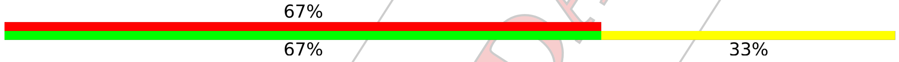

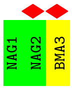

• Molecule 5: beta-D-mannopyranose-(1-4)-2-acetamido-2-deoxy-beta-D-glucopyranose-(1-4)-2-acetamido-2-deoxy-beta-D-glucopyranose

Chain w:

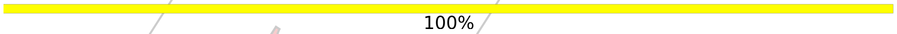

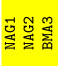

• Molecule 5: beta-D-mannopyranose-(1-4)-2-acetamido-2-deoxy-beta-D-glucopyranose-(1-4)-2-acetamido-2-deoxy-beta-D-glucopyranose

Chain x:

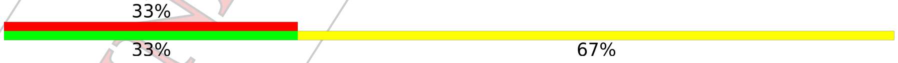

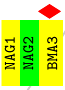

• Molecule 5: beta-D-mannopyranose-(1-4)-2-acetamido-2-deoxy-beta-D-glucopyranose-(1-4)-2-acetamido-2-deoxy-beta-D-glucopyranose

Chain 1:

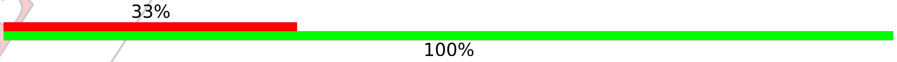

PRELIMINARY VALIDATION REPORT

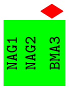

• Molecule 5: beta-D-mannopyranose-(1-4)-2-acetamido-2-deoxy-beta-D-glucopyranose-(1-4)-2-ac etamido-2-deoxy-beta-D-glucopyranose

Chain 6:

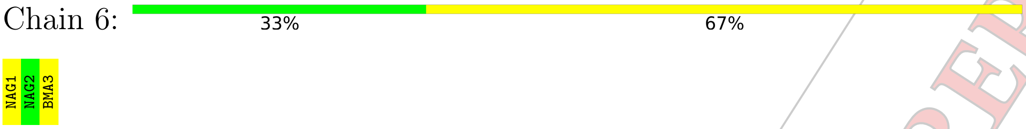

• Molecule 5: beta-D-mannopyranose-(1-4)-2-acetamido-2-deoxy-beta-D-glucopyranose-(1-4)-2-ac etamido-2-deoxy-beta-D-glucopyranose

Chain 7:

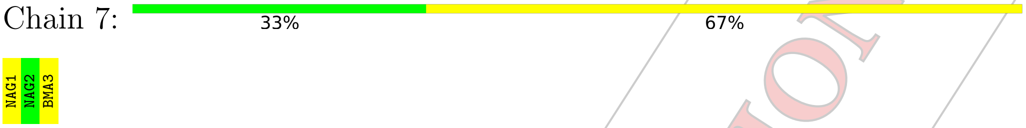

• Molecule 6: 2-acetamido-2-deoxy-beta-D-glucopyranose-(1-4)-2-acetamido-2-deoxy-beta-D-gluc opyranose

Chain N:

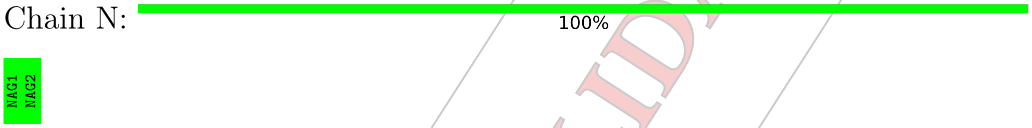

• Molecule 6: 2-acetamido-2-deoxy-beta-D-glucopyranose-(1-4)-2-acetamido-2-deoxy-beta-D-gluc opyranose

Chain Q:

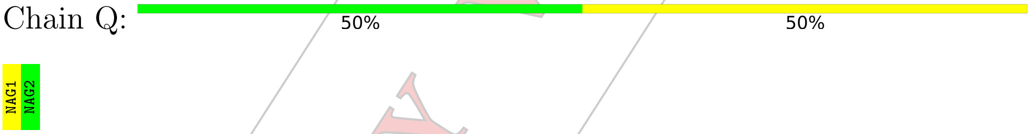

• Molecule 6: 2-acetamido-2-deoxy-beta-D-glucopyranose-(1-4)-2-acetamido-2-deoxy-beta-D-gluc opyranose

Chain R:

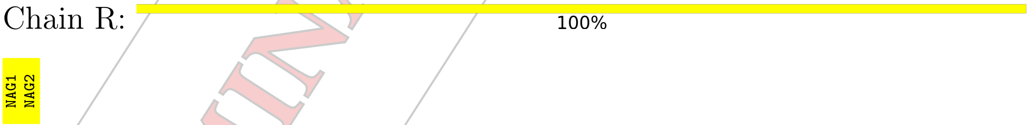

• Molecule 6: 2-acetamido-2-deoxy-beta-D-glucopyranose-(1-4)-2-acetamido-2-deoxy-beta-D-gluc opyranose

Chain X:

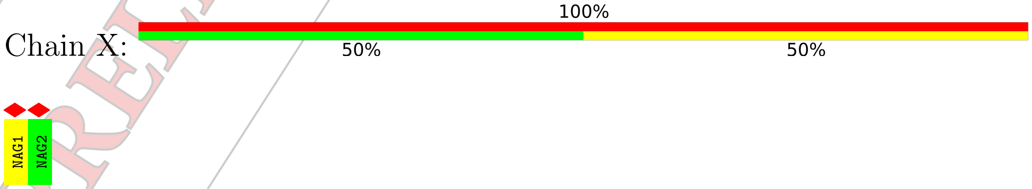

PRELIMINARY VALIDATION REPORT

• Molecule 6: 2-acetamido-2-deoxy-beta-D-glucopyranose-(1-4)-2-acetamido-2-deoxy-beta-D-glucopyranose

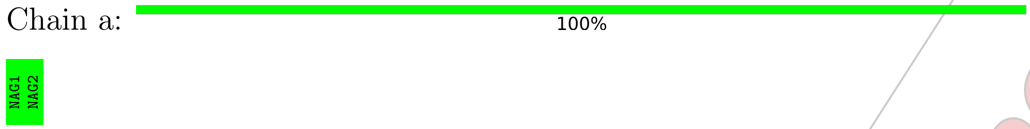

• Molecule 6: 2-acetamido-2-deoxy-beta-D-glucopyranose-(1-4)-2-acetamido-2-deoxy-beta-D-glucopyranose

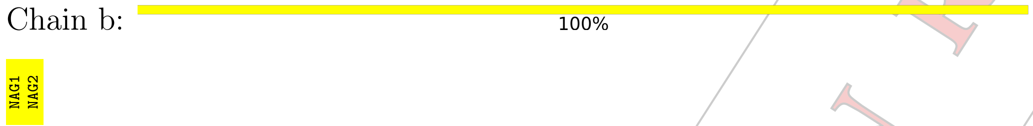

• Molecule 6: 2-acetamido-2-deoxy-beta-D-glucopyranose-(1-4)-2-acetamido-2-deoxy-beta-D-glucopyranose

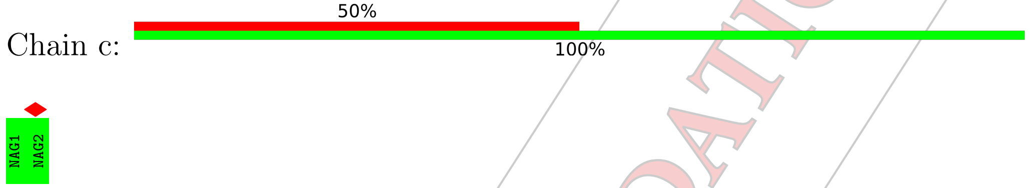

• Molecule 6: 2-acetamido-2-deoxy-beta-D-glucopyranose-(1-4)-2-acetamido-2-deoxy-beta-D-glucopyranose

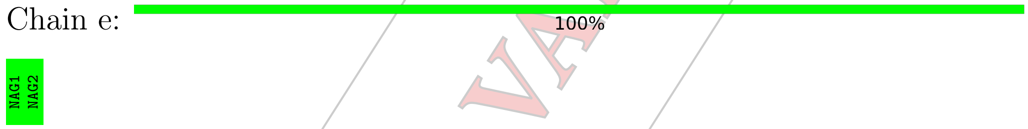

• Molecule 6: 2-acetamido-2-deoxy-beta-D-glucopyranose-(1-4)-2-acetamido-2-deoxy-beta-D-glucopyranose

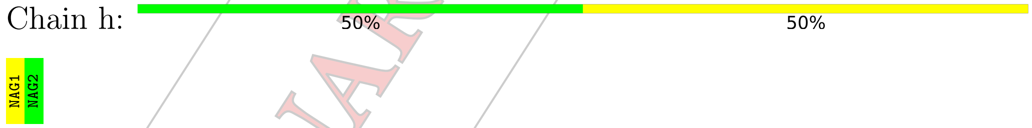

• Molecule 6: 2-acetamido-2-deoxy-beta-D-glucopyranose-(1-4)-2-acetamido-2-deoxy-beta-D-glucopyranose

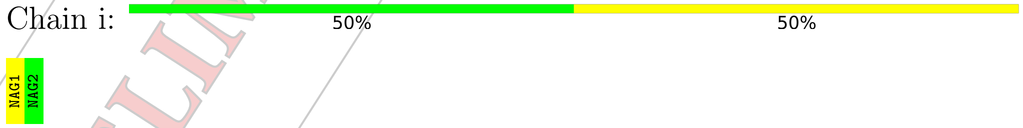

• Molecule 6: 2-acetamido-2-deoxy-beta-D-glucopyranose-(1-4)-2-acetamido-2-deoxy-beta-D-glucopyranose

PRELIMINARY VALIDATION REPORT

Chain o:

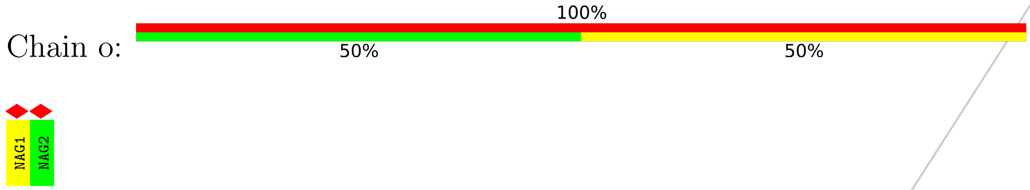

• Molecule 6: 2-acetamido-2-deoxy-beta-D-glucopyranose-(1-4)-2-acetamido-2-deoxy-beta-D-glucopyranose

Chain r:

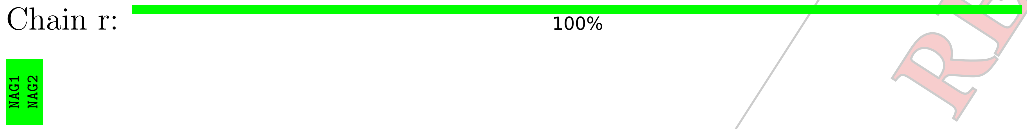

• Molecule 6: 2-acetamido-2-deoxy-beta-D-glucopyranose-(1-4)-2-acetamido-2-deoxy-beta-D-glucopyranose

Chain s:

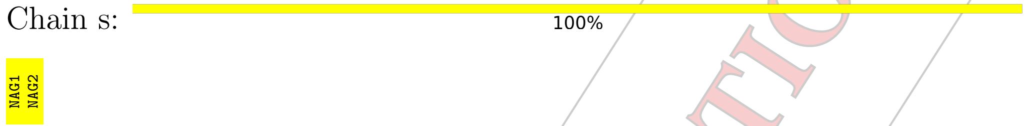

• Molecule 6: 2-acetamido-2-deoxy-beta-D-glucopyranose-(1-4)-2-acetamido-2-deoxy-beta-D-glucopyranose

Chain t:

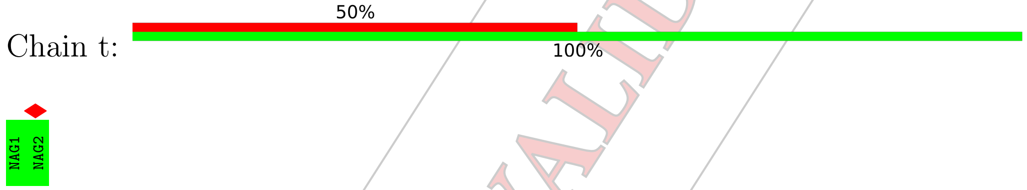

• Molecule 6: 2-acetamido-2-deoxy-beta-D-glucopyranose-(1-4)-2-acetamido-2-deoxy-beta-D-glucopyranose

Chain v:

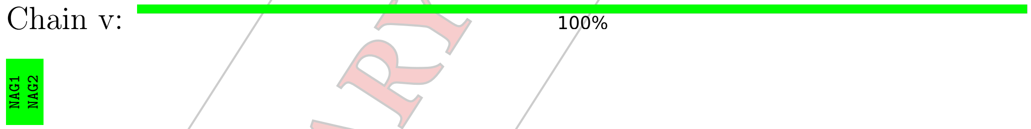

• Molecule 6: 2-acetamido-2-deoxy-beta-D-glucopyranose-(1-4)-2-acetamido-2-deoxy-beta-D-glucopyranose

Chain y:

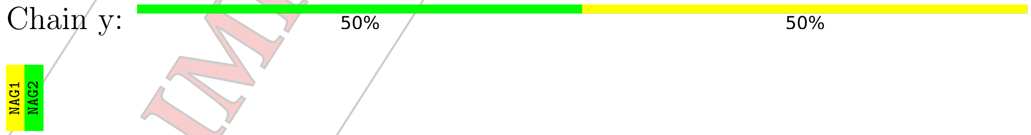

• Molecule 6: 2-acetamido-2-deoxy-beta-D-glucopyranose-(1-4)-2-acetamido-2-deoxy-beta-D-glucopyranose

Chain z:

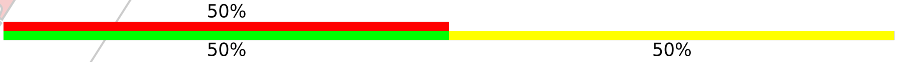

PRELIMINARY VALIDATION REPORT

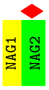

• Molecule 6: 2-acetamido-2-deoxy-beta-D-glucopyranose-(1-4)-2-acetamido-2-deoxy-beta-D-gluc opyranose

Chain 5:

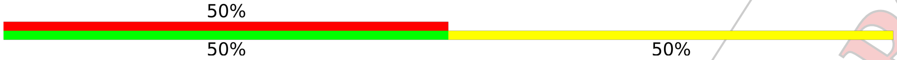

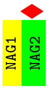

• Molecule 6: 2-acetamido-2-deoxy-beta-D-glucopyranose-(1-4)-2-acetamido-2-deoxy-beta-D-gluc opyranose

Chain 8:

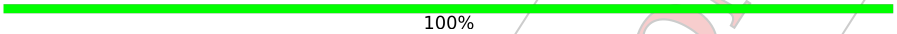

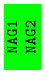

• Molecule 6: 2-acetamido-2-deoxy-beta-D-glucopyranose-(1-4)-2-acetamido-2-deoxy-beta-D-gluc opyranose

Chain 9:

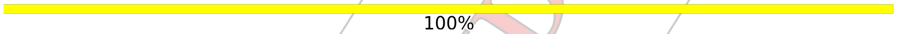

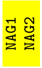

• Molecule 6: 2-acetamido-2-deoxy-beta-D-glucopyranose-(1-4)-2-acetamido-2-deoxy-beta-D-gluc opyranose

Chain AA:

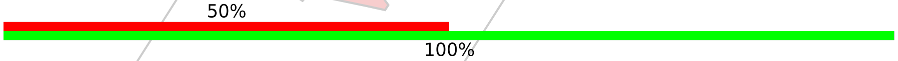

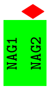

• Molecule 7: alpha-D-mannopyranose-(1-2)-alpha-D-mannopyranose-(1-3)-[alpha-D-mannopyran ose-(1-3)-alpha-D-mannopyranose-(1-6)]beta-D-mannopyranose-(1-4)-2-acetamido-2-deoxy-beta-D-glucopyranose-(1-4)-2-acetamido-2-deoxy-beta-D-glucopyranose

Chain S:

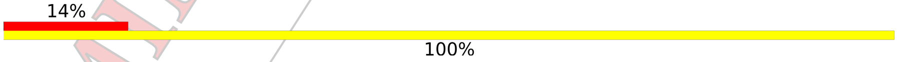

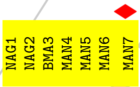

• Molecule 7: alpha-D-mannopyranose-(1-2)-alpha-D-mannopyranose-(1-3)-[alpha-D-mannopyran ose-(1-3)-alpha-D-mannopyranose-(1-6)]beta-D-mannopyranose-(1-4)-2-acetamido-2-deoxy-beta-D-glucopyranose-(1-4)-2-acetamido-2-deoxy-beta-D-glucopyranose

PRELIMINARY VALIDATION REPORT

Chain j:

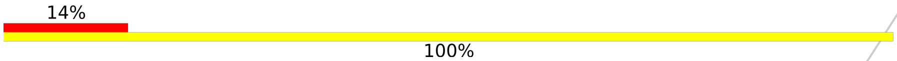

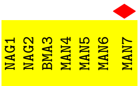

• Molecule 7: alpha-D-mannopyranose-(1-2)-alpha-D-mannopyranose-(1-3)-[alpha-D-mannopyranose-(1-3)-alpha-D-mannopyranose-(1-6)]beta-D-mannopyranose-(1-4)-2-acetamido-2-deoxy-beta-D-glucopyranose-(1-4)-2-acetamido-2-deoxy-beta-D-glucopyranose

Chain 0:

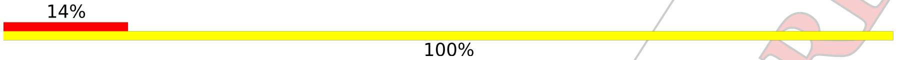

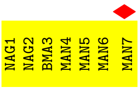

• Molecule 8: alpha-D-mannopyranose-(1-3)-[alpha-D-mannopyranose-(1-6)]beta-D-mannopyranose-(1-4)-2-acetamido-2-deoxy-beta-D-glucopyranose-(1-4)-2-acetamido-2-deoxy-beta-D-glucopyranose

Chain U:

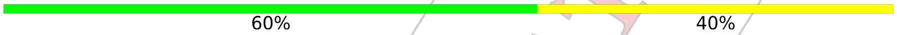

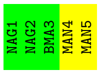

• Molecule 8: alpha-D-mannopyranose-(1-3)-[alpha-D-mannopyranose-(1-6)]beta-D-mannopyranose-(1-4)-2-acetamido-2-deoxy-beta-D-glucopyranose-(1-4)-2-acetamido-2-deoxy-beta-D-glucopyranose

Chain l:

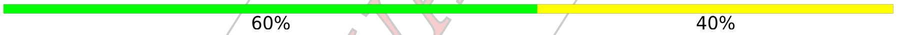

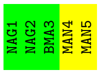

• Molecule 8: alpha-D-mannopyranose-(1-3)-[alpha-D-mannopyranose-(1-6)]beta-D-mannopyranose-(1-4)-2-acetamido-2-deoxy-beta-D-glucopyranose-(1-4)-2-acetamido-2-deoxy-beta-D-glucopyranose

Chain 2:

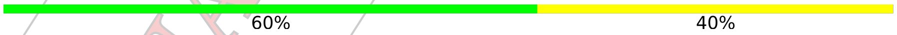

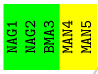

• Molecule 9: alpha-D-mannopyranose-(1-3)-[alpha-D-mannopyranose-(1-6)]alpha-D-mannopyranose-(1-6)-[alpha-D-mannopyranose-(1-3)]beta-D-mannopyranose-(1-4)-2-acetamido-2-deoxy-beta-D-glucopyranose-(1-4)-2-acetamido-2-deoxy-beta-D-glucopyranose

Chain V:

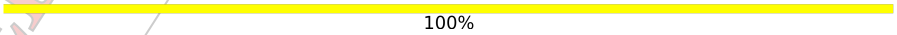

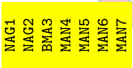

PRELIMINARY VALIDATION REPORT

- Molecule 9: alpha-D-mannopyranose-(1-3)-[alpha-D-mannopyranose-(1-6)]alpha-D-mannopyranose-(1-6)-[alpha-D-mannopyranose-(1-3)]beta-D-mannopyranose-(1-4)-2-acetamido-2-deoxy-beta-D-glucopyranose-(1-4)-2-acetamido-2-deoxy-beta-D-glucopyranose

Chain m:

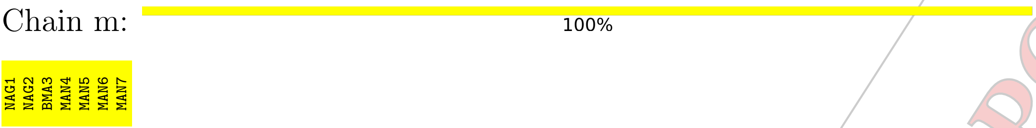

- Molecule 9: alpha-D-mannopyranose-(1-3)-[alpha-D-mannopyranose-(1-6)]alpha-D-mannopyranose-(1-6)-[alpha-D-mannopyranose-(1-3)]beta-D-mannopyranose-(1-4)-2-acetamido-2-deoxy-beta-D-glucopyranose-(1-4)-2-acetamido-2-deoxy-beta-D-glucopyranose

Chain 3:

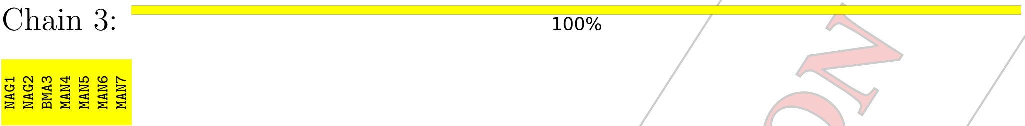

- Molecule 10: alpha-D-mannopyranose-(1-2)-alpha-D-mannopyranose-(1-6)-[alpha-D-mannopyranose-(1-3)]alpha-D-mannopyranose-(1-6)-[alpha-D-mannopyranose-(1-2)-alpha-D-mannopyranose-(1-3)]beta-D-mannopyranose-(1-4)-2-acetamido-2-deoxy-beta-D-glucopyranose-(1-4)-2-acetamido-2-deoxy-beta-D-glucopyranose

Chain W:

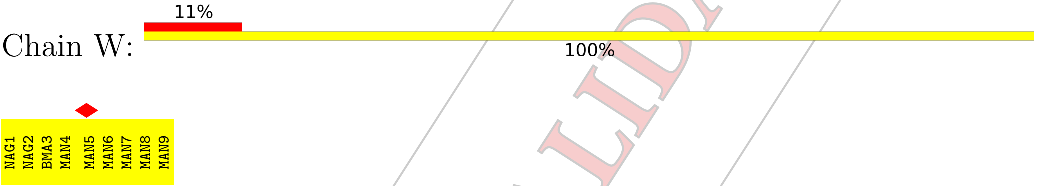

- Molecule 10: alpha-D-mannopyranose-(1-2)-alpha-D-mannopyranose-(1-6)-[alpha-D-mannopyranose-(1-3)]alpha-D-mannopyranose-(1-6)-[alpha-D-mannopyranose-(1-2)-alpha-D-mannopyranose-(1-3)]beta-D-mannopyranose-(1-4)-2-acetamido-2-deoxy-beta-D-glucopyranose-(1-4)-2-acetamido-2-deoxy-beta-D-glucopyranose

Chain n:

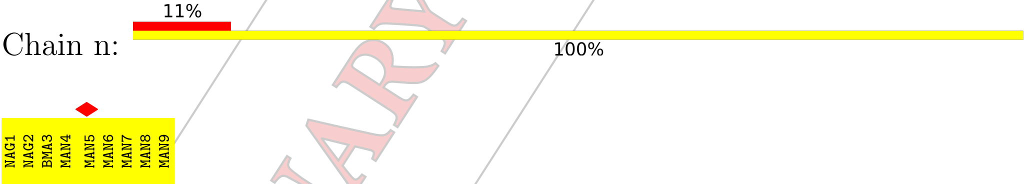

- Molecule 10: alpha-D-mannopyranose-(1-2)-alpha-D-mannopyranose-(1-6)-[alpha-D-mannopyranose-(1-3)]alpha-D-mannopyranose-(1-6)-[alpha-D-mannopyranose-(1-2)-alpha-D-mannopyranose-(1-3)]beta-D-mannopyranose-(1-4)-2-acetamido-2-deoxy-beta-D-glucopyranose-(1-4)-2-acetamido-2-deoxy-beta-D-glucopyranose

Chain 4:

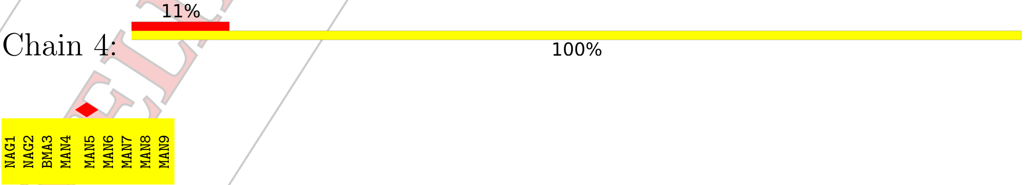

PRELIMINARY VALIDATION REPORT

4 Experimental information ⓘ

Property	Value	Source
EM reconstruction method	SINGLE PARTICLE	Depositor
Imposed symmetry	POINT, Not provided	
Number of particles used	Not provided	
Resolution determination method	Not provided	
CTF correction method	Not provided	
Microscope	Not provided	
Voltage (kV)	Not provided	
Electron dose ($e^-/\text{Å}^2$)	Not provided	
Minimum defocus (nm)	Not provided	
Maximum defocus (nm)	Not provided	
Magnification	Not provided	
Image detector	Not provided	
Maximum map value	56.840	Depositor
Minimum map value	-26.521	Depositor
Average map value	-0.000	Depositor
Map value standard deviation	1.000	Depositor
Recommended contour level	7.8	Depositor
Map size (Å)	540.0, 540.0, 540.0	wwPDB
Map dimensions	400, 400, 400	wwPDB
Map angles (°)	90.0, 90.0, 90.0	wwPDB
Pixel spacing (Å)	1.35, 1.35, 1.35	Depositor

PRELIMINARY VALIDATION REPORT

5 Model quality (i)

5.1 Standard geometry (i)

Bond lengths and bond angles in the following residue types are not validated in this section: NAG, BMA, MAN

The Z score for a bond length (or angle) is the number of standard deviations the observed value is removed from the expected value. A bond length (or angle) with $|Z| > 5$ is considered an outlier worth inspection. RMSZ is the root-mean-square of all Z scores of the bond lengths (or angles).

Mol	Chain	Bond lengths		Bond angles	
		RMSZ	#$|Z|$ >5	RMSZ	#$|Z|$ >5
1	A	0.60	1/3597 (0.0%)	0.68	3/4888 (0.1%)
1	E	0.60	1/3597 (0.0%)	0.68	3/4888 (0.1%)
1	I	0.60	1/3597 (0.0%)	0.68	3/4888 (0.1%)
2	B	0.46	0/1074	0.59	0/1455
2	F	0.46	0/1074	0.59	0/1455
2	J	0.46	0/1074	0.59	0/1455
3	C	0.44	0/1040	0.59	0/1411
3	G	0.44	0/1040	0.59	0/1411
3	K	0.44	0/1040	0.59	0/1411
4	D	0.44	0/794	0.63	2/1078 (0.2%)
4	H	0.44	0/794	0.63	2/1078 (0.2%)
4	L	0.44	0/794	0.63	2/1078 (0.2%)
All	All	0.54	3/19515 (0.0%)	0.65	15/26496 (0.1%)

Chiral center outliers are detected by calculating the chiral volume of a chiral center and verifying if the center is modelled as a planar moiety or with the opposite hand.A planarity outlier is detected by checking planarity of atoms in a peptide group, atoms in a mainchain group or atoms of a sidechain that are expected to be planar.

Mol	Chain	#Chirality outliers	#Planarity outliers
1	A	0	2
1	E	0	2
1	I	0	2
All	All	0	6

All (3) bond length outliers are listed below:

Mol	Chain	Res	Type	Atoms	Z	Observed(Å)	Ideal(Å)
1	A	218	CYS	C-N	-6.35	1.19	1.34
1	I	218	CYS	C-N	-6.35	1.19	1.34
1	E	218	CYS	C-N	-6.31	1.19	1.34

PRELIMINARY VALIDATION REPORT

All (15) bond angle outliers are listed below:

Mol	Chain	Res	Type	Atoms	Z	Observed(°)	Ideal(°)
1	A	445	CYS	CA-CB-SG	6.75	126.16	114.00
1	I	445	CYS	CA-CB-SG	6.75	126.14	114.00
1	E	445	CYS	CA-CB-SG	6.74	126.13	114.00
1	A	247	CYS	CA-CB-SG	6.59	125.86	114.00
1	E	247	CYS	CA-CB-SG	6.59	125.85	114.00
1	I	247	CYS	CA-CB-SG	6.58	125.84	114.00
4	L	93	TYR	CG-CD2-CE2	-6.04	116.47	121.30
4	H	93	TYR	CG-CD2-CE2	-6.02	116.49	121.30
4	D	93	TYR	CG-CD2-CE2	-6.01	116.49	121.30
1	E	65	ALA	N-CA-C	5.97	127.12	111.00
1	A	65	ALA	N-CA-C	5.96	127.10	111.00
1	I	65	ALA	N-CA-C	5.96	127.09	111.00
4	L	93	TYR	CB-CG-CD1	-5.41	117.76	121.00
4	H	93	TYR	CB-CG-CD1	-5.38	117.78	121.00
4	D	93	TYR	CB-CG-CD1	-5.36	117.78	121.00

There are no chirality outliers.

All (6) planarity outliers are listed below:

Mol	Chain	Res	Type	Group
1	A	501	CYS	Peptide
1	A	66	ALA	Peptide
1	E	501	CYS	Peptide
1	E	66	ALA	Peptide
1	I	501	CYS	Peptide
1	I	66	ALA	Peptide

5.2 Too-close contacts (i)

Due to software issues we are unable to calculate clashes - this section is therefore empty.

5.3 Torsion angles (i)

5.3.1 Protein backbone (i)

In the following table, the Percentiles column shows the percent Ramachandran outliers of the chain as a percentile score with respect to all PDB entries followed by that with respect to all EM entries.

PRELIMINARY VALIDATION REPORT

The Analysed column shows the number of residues for which the backbone conformation was analysed, and the total number of residues.

Mol	Chain	Analysed	Favoured	Allowed	Outliers	Percentiles	
1	A	444/481 (92%)	375 (84%)	68 (15%)	1 (0%)	47	47
1	E	444/481 (92%)	375 (84%)	68 (15%)	1 (0%)	47	47
1	I	444/481 (92%)	375 (84%)	68 (15%)	1 (0%)	47	47
2	B	129/153 (84%)	103 (80%)	25 (19%)	1 (1%)	19	19
2	F	129/153 (84%)	103 (80%)	25 (19%)	1 (1%)	19	19
2	J	129/153 (84%)	103 (80%)	26 (20%)	0	100	100
3	C	126/128 (98%)	107 (85%)	19 (15%)	0	100	100
3	G	126/128 (98%)	107 (85%)	19 (15%)	0	100	100
3	K	126/128 (98%)	107 (85%)	19 (15%)	0	100	100
4	D	105/108 (97%)	93 (89%)	12 (11%)	0	100	100
4	H	105/108 (97%)	93 (89%)	12 (11%)	0	100	100
4	L	105/108 (97%)	93 (89%)	12 (11%)	0	100	100
All	All	2412/2610 (92%)	2034 (84%)	373 (16%)	5 (0%)	50	47

All (5) Ramachandran outliers are listed below:

Mol	Chain	Res	Type
1	A	67	ALA
1	E	67	ALA
1	I	67	ALA
2	B	549	VAL
2	F	549	VAL

5.3.2 Protein sidechains (i)

In the following table, the Percentiles column shows the percent sidechain outliers of the chain as a percentile score with respect to all PDB entries followed by that with respect to all EM entries.

The Analysed column shows the number of residues for which the sidechain conformation was analysed, and the total number of residues.

Mol	Chain	Analysed	Rotameric	Outliers	Percentiles	
1	A	395/418 (94%)	379 (96%)	16 (4%)	30	30
1	E	395/418 (94%)	379 (96%)	16 (4%)	30	30

Continued on next page...

PRELIMINARY VALIDATION REPORT

Continued from previous page...

Mol	Chain	Analysed	Rotameric	Outliers	Percentiles	
1	I	395/418 (94%)	379 (96%)	16 (4%)	30	30
2	B	114/128 (89%)	109 (96%)	5 (4%)	28	28
2	F	114/128 (89%)	109 (96%)	5 (4%)	28	28
2	J	114/128 (89%)	109 (96%)	5 (4%)	28	28
3	C	106/106 (100%)	103 (97%)	3 (3%)	43	43
3	G	106/106 (100%)	103 (97%)	3 (3%)	43	43
3	K	106/106 (100%)	103 (97%)	3 (3%)	43	43
4	D	85/86 (99%)	82 (96%)	3 (4%)	36	36
4	H	85/86 (99%)	82 (96%)	3 (4%)	36	36
4	L	85/86 (99%)	82 (96%)	3 (4%)	36	36
All	All	2100/2214 (95%)	2019 (96%)	81 (4%)	36	32

All (81) residues with a non-rotameric sidechain are listed below:

Mol	Chain	Res	Type
1	A	36	VAL
1	A	47	ASP
1	A	68	VAL
1	A	75	VAL
1	A	92	GLU
1	A	106	THR
1	A	115	SER
1	A	208	VAL
1	A	209	SER
1	A	218	CYS
1	A	285	LEU
1	A	320	THR
1	A	359	ILE
1	A	442	VAL
1	A	447	SER
1	A	496	VAL
2	B	627	THR
2	B	636	SER
2	B	639	THR
2	B	645	LEU
2	B	663	LEU
3	C	37	VAL
3	C	104	VAL

Continued on next page...

PRELIMINARY VALIDATION REPORT

Continued from previous page...

Mol	Chain	Res	Type
3	C	105	TYR
4	D	2	SER
4	D	53	SER
4	D	69	SER
1	E	36	VAL
1	E	47	ASP
1	E	68	VAL
1	E	75	VAL
1	E	92	GLU
1	E	106	THR
1	E	115	SER
1	E	208	VAL
1	E	209	SER
1	E	218	CYS
1	E	285	LEU
1	E	320	THR
1	E	359	ILE
1	E	442	VAL
1	E	447	SER
1	E	496	VAL
2	F	627	THR
2	F	636	SER
2	F	639	THR
2	F	645	LEU
2	F	663	LEU
3	G	37	VAL
3	G	104	VAL
3	G	105	TYR
4	H	2	SER
4	H	53	SER
4	H	69	SER
1	I	36	VAL
1	I	47	ASP
1	I	68	VAL
1	I	75	VAL
1	I	92	GLU
1	I	106	THR
1	I	115	SER
1	I	208	VAL
1	I	209	SER
1	I	218	CYS
1	I	285	LEU

Continued on next page...

PRELIMINARY VALIDATION REPORT

Continued from previous page...

Mol	Chain	Res	Type
1	I	320	THR
1	I	359	ILE
1	I	442	VAL
1	I	447	SER
1	I	496	VAL
2	J	627	THR
2	J	636	SER
2	J	639	THR
2	J	645	LEU
2	J	663	LEU
3	K	37	VAL
3	K	104	VAL
3	K	105	TYR
4	L	2	SER
4	L	53	SER
4	L	69	SER

Sometimes sidechains can be flipped to improve hydrogen bonding and reduce clashes. All (36) such sidechains are listed below:

Mol	Chain	Res	Type
1	A	82	GLN
1	A	105	HIS
1	A	114	GLN
1	A	195	ASN
1	A	374	HIS
1	A	422	GLN
1	A	425	ASN
2	B	650	GLN
3	C	52	ASN
3	C	77	ASN
4	D	16	GLN
4	D	91	GLN
1	E	82	GLN
1	E	105	HIS
1	E	114	GLN
1	E	195	ASN
1	E	374	HIS
1	E	422	GLN
1	E	425	ASN
2	F	650	GLN
2	F	656	ASN
3	G	52	ASN

Continued on next page...

PRELIMINARY VALIDATION REPORT

Continued from previous page...

Mol	Chain	Res	Type
3	G	77	ASN
4	H	16	GLN
1	I	82	GLN
1	I	105	HIS
1	I	114	GLN
1	I	195	ASN
1	I	374	HIS
1	I	422	GLN
1	I	425	ASN
2	J	650	GLN
3	K	52	ASN
3	K	77	ASN
4	L	16	GLN
4	L	91	GLN

5.3.3 RNA (i)

There are no RNA molecules in this entry.

5.4 Non-standard residues in protein, DNA, RNA chains (i)

There are no non-standard protein/DNA/RNA residues in this entry.

5.5 Carbohydrates (i)

180 monosaccharides are modelled in this entry.

In the following table, the Counts columns list the number of bonds (or angles) for which Mogul statistics could be retrieved, the number of bonds (or angles) that are observed in the model and the number of bonds (or angles) that are defined in the Chemical Component Dictionary. The Link column lists molecule types, if any, to which the group is linked. The Z score for a bond length (or angle) is the number of standard deviations the observed value is removed from the expected value. A bond length (or angle) with $|Z| > 2$ is considered an outlier worth inspection. RMSZ is the root-mean-square of all Z scores of the bond lengths (or angles).

Mol	Type	Chain	Res	Link	Bond lengths			Bond angles		
					Counts	RMSZ	$\#\|Z\| > 2$	Counts	RMSZ	$\#\|Z\| > 2$
7	NAG	0	1	1,7	14,14,15	1.00	1 (7%)	17,19,21	0.48	0
7	NAG	0	2	7	14,14,15	0.77	1 (7%)	17,19,21	0.68	0
7	BMA	0	3	7	11,11,12	0.91	1 (9%)	15,15,17	0.86	0
7	MAN	0	4	7	11,11,12	0.76	0	15,15,17	1.35	2 (13%)

PRELIMINARY VALIDATION REPORT

Mol	Type	Chain	Res	Link	Bond lengths			Bond angles		
					Counts	RMSZ	#|Z| > 2	Counts	RMSZ	#|Z| > 2
7	MAN	0	5	7	11,11,12	0.65	0	15,15,17	1.53	2 (13%)
7	MAN	0	6	7	11,11,12	0.61	0	15,15,17	0.95	1 (6%)
7	MAN	0	7	7	11,11,12	0.49	0	15,15,17	1.13	2 (13%)
5	NAG	1	1	1,5	14,14,15	0.50	0	17,19,21	0.33	0
5	NAG	1	2	5	14,14,15	0.21	0	17,19,21	0.45	0
5	BMA	1	3	5	11,11,12	0.49	0	15,15,17	0.82	0
8	NAG	2	1	1,8	14,14,15	0.47	0	17,19,21	0.52	0
8	NAG	2	2	8	14,14,15	0.31	0	17,19,21	0.47	0
8	BMA	2	3	8	11,11,12	0.62	0	15,15,17	0.70	0
8	MAN	2	4	8	11,11,12	0.49	0	15,15,17	1.07	2 (13%)
8	MAN	2	5	8	11,11,12	0.68	0	15,15,17	1.06	1 (6%)
9	NAG	3	1	1,9	14,14,15	0.29	0	17,19,21	1.11	1 (5%)
9	NAG	3	2	9	14,14,15	1.50	1 (7%)	17,19,21	1.12	1 (5%)
9	BMA	3	3	9	11,11,12	1.44	1 (9%)	15,15,17	1.38	2 (13%)
9	MAN	3	4	9	11,11,12	0.99	1 (9%)	15,15,17	1.17	1 (6%)
9	MAN	3	5	9	11,11,12	1.42	1 (9%)	15,15,17	1.91	4 (26%)
9	MAN	3	6	9	11,11,12	0.85	1 (9%)	15,15,17	0.92	1 (6%)
9	MAN	3	7	9	11,11,12	1.05	1 (9%)	15,15,17	1.12	1 (6%)
10	NAG	4	1	10,1	14,14,15	0.61	1 (7%)	17,19,21	0.52	0
10	NAG	4	2	10	14,14,15	0.86	1 (7%)	17,19,21	0.57	0
10	BMA	4	3	10	11,11,12	1.24	2 (18%)	15,15,17	1.35	3 (20%)
10	MAN	4	4	10	11,11,12	0.52	0	15,15,17	1.15	2 (13%)
10	MAN	4	5	10	11,11,12	1.03	1 (9%)	15,15,17	1.72	3 (20%)
10	MAN	4	6	10	11,11,12	2.82	7 (63%)	15,15,17	2.01	4 (26%)
10	MAN	4	7	10	11,11,12	0.70	0	15,15,17	0.94	1 (6%)
10	MAN	4	8	10	11,11,12	1.66	1 (9%)	15,15,17	1.42	3 (20%)
10	MAN	4	9	10	11,11,12	1.59	2 (18%)	15,15,17	1.57	3 (20%)
6	NAG	5	1	1,6	14,14,15	0.73	1 (7%)	17,19,21	0.56	0
6	NAG	5	2	6	14,14,15	0.37	0	17,19,21	0.45	0
5	NAG	6	1	1,5	14,14,15	1.02	1 (7%)	17,19,21	0.72	0
5	NAG	6	2	5	14,14,15	0.36	0	17,19,21	0.47	0
5	BMA	6	3	5	11,11,12	0.84	1 (9%)	15,15,17	0.91	0
5	NAG	7	1	1,5	14,14,15	0.58	1 (7%)	17,19,21	0.51	0
5	NAG	7	2	5	14,14,15	0.21	0	17,19,21	0.52	0
5	BMA	7	3	5	11,11,12	0.63	0	15,15,17	0.97	1 (6%)
6	NAG	8	1	1,6	14,14,15	0.31	0	17,19,21	0.46	0
6	NAG	8	2	6	14,14,15	0.35	0	17,19,21	0.49	0

PRELIMINARY VALIDATION REPORT

Mol	Type	Chain	Res	Link	Bond lengths			Bond angles		
					Counts	RMSZ	#|Z| > 2	Counts	RMSZ	#|Z| > 2
6	NAG	9	1	1,6	14,14,15	0.80	1 (7%)	17,19,21	0.67	0
6	NAG	9	2	6	14,14,15	0.25	0	17,19,21	0.71	1 (5%)
6	NAG	AA	1	2,6	14,14,15	0.41	0	17,19,21	0.40	0
6	NAG	AA	2	6	14,14,15	0.31	0	17,19,21	0.35	0
5	NAG	M	1	1,5	14,14,15	0.34	0	17,19,21	0.65	1 (5%)
5	NAG	M	2	5	14,14,15	0.23	0	17,19,21	0.40	0
5	BMA	M	3	5	11,11,12	0.60	0	15,15,17	1.00	1 (6%)
6	NAG	N	1	1,6	14,14,15	0.45	0	17,19,21	0.73	0
6	NAG	N	2	6	14,14,15	0.28	0	17,19,21	0.53	0
5	NAG	O	1	1,5	14,14,15	0.85	1 (7%)	17,19,21	0.55	0
5	NAG	O	2	5	14,14,15	0.69	1 (7%)	17,19,21	0.67	0
5	BMA	O	3	5	11,11,12	0.49	0	15,15,17	0.96	1 (6%)
5	NAG	P	1	1,5	14,14,15	1.39	2 (14%)	17,19,21	0.67	0
5	NAG	P	2	5	14,14,15	0.22	0	17,19,21	0.37	0
5	BMA	P	3	5	11,11,12	0.55	0	15,15,17	0.85	1 (6%)
6	NAG	Q	1	1,6	14,14,15	0.57	0	17,19,21	0.96	1 (5%)
6	NAG	Q	2	6	14,14,15	0.23	0	17,19,21	0.62	0
6	NAG	R	1	1,6	14,14,15	0.80	1 (7%)	17,19,21	0.67	0
6	NAG	R	2	6	14,14,15	0.58	1 (7%)	17,19,21	0.56	0
7	NAG	S	1	1,7	14,14,15	1.00	1 (7%)	17,19,21	0.47	0
7	NAG	S	2	7	14,14,15	0.76	1 (7%)	17,19,21	0.68	0
7	BMA	S	3	7	11,11,12	0.92	1 (9%)	15,15,17	0.85	0
7	MAN	S	4	7	11,11,12	0.78	0	15,15,17	1.34	2 (13%)
7	MAN	S	5	7	11,11,12	0.65	0	15,15,17	1.53	2 (13%)
7	MAN	S	6	7	11,11,12	0.61	0	15,15,17	0.95	1 (6%)
7	MAN	S	7	7	11,11,12	0.49	0	15,15,17	1.14	2 (13%)
5	NAG	T	1	1,5	14,14,15	0.49	0	17,19,21	0.34	0
5	NAG	T	2	5	14,14,15	0.22	0	17,19,21	0.45	0
5	BMA	T	3	5	11,11,12	0.49	0	15,15,17	0.83	0
8	NAG	U	1	1,8	14,14,15	0.46	0	17,19,21	0.52	0
8	NAG	U	2	8	14,14,15	0.32	0	17,19,21	0.48	0
8	BMA	U	3	8	11,11,12	0.63	0	15,15,17	0.70	0
8	MAN	U	4	8	11,11,12	0.50	0	15,15,17	1.06	2 (13%)
8	MAN	U	5	8	11,11,12	0.67	0	15,15,17	1.05	1 (6%)
9	NAG	V	1	1,9	14,14,15	0.29	0	17,19,21	1.11	1 (5%)
9	NAG	V	2	9	14,14,15	1.51	1 (7%)	17,19,21	1.12	1 (5%)
9	BMA	V	3	9	11,11,12	1.44	1 (9%)	15,15,17	1.37	2 (13%)

PRELIMINARY VALIDATION REPORT

Mol	Type	Chain	Res	Link	Bond lengths			Bond angles		
					Counts	RMSZ	#|Z| > 2	Counts	RMSZ	#|Z| > 2
9	MAN	V	4	9	11,11,12	0.98	1 (9%)	15,15,17	1.17	1 (6%)
9	MAN	V	5	9	11,11,12	1.43	1 (9%)	15,15,17	1.91	4 (26%)
9	MAN	V	6	9	11,11,12	0.84	1 (9%)	15,15,17	0.91	1 (6%)
9	MAN	V	7	9	11,11,12	1.04	1 (9%)	15,15,17	1.13	1 (6%)
10	NAG	W	1	10,1	14,14,15	0.61	1 (7%)	17,19,21	0.53	0
10	NAG	W	2	10	14,14,15	0.87	1 (7%)	17,19,21	0.57	0
10	BMA	W	3	10	11,11,12	1.24	2 (18%)	15,15,17	1.35	3 (20%)
10	MAN	W	4	10	11,11,12	0.52	0	15,15,17	1.15	2 (13%)
10	MAN	W	5	10	11,11,12	1.04	1 (9%)	15,15,17	1.71	3 (20%)
10	MAN	W	6	10	11,11,12	2.81	7 (63%)	15,15,17	2.01	4 (26%)
10	MAN	W	7	10	11,11,12	0.71	0	15,15,17	0.93	1 (6%)
10	MAN	W	8	10	11,11,12	1.67	1 (9%)	15,15,17	1.42	3 (20%)
10	MAN	W	9	10	11,11,12	1.59	2 (18%)	15,15,17	1.56	3 (20%)
6	NAG	X	1	1,6	14,14,15	0.72	1 (7%)	17,19,21	0.56	0
6	NAG	X	2	6	14,14,15	0.36	0	17,19,21	0.45	0
5	NAG	Y	1	1,5	14,14,15	1.03	1 (7%)	17,19,21	0.72	0
5	NAG	Y	2	5	14,14,15	0.38	0	17,19,21	0.47	0
5	BMA	Y	3	5	11,11,12	0.83	1 (9%)	15,15,17	0.92	0
5	NAG	Z	1	1,5	14,14,15	0.58	1 (7%)	17,19,21	0.51	0
5	NAG	Z	2	5	14,14,15	0.21	0	17,19,21	0.53	0
5	BMA	Z	3	5	11,11,12	0.64	0	15,15,17	0.97	1 (6%)
6	NAG	a	1	1,6	14,14,15	0.32	0	17,19,21	0.46	0
6	NAG	a	2	6	14,14,15	0.35	0	17,19,21	0.49	0
6	NAG	b	1	1,6	14,14,15	0.80	1 (7%)	17,19,21	0.68	0
6	NAG	b	2	6	14,14,15	0.26	0	17,19,21	0.72	1 (5%)
6	NAG	c	1	2,6	14,14,15	0.41	0	17,19,21	0.39	0
6	NAG	c	2	6	14,14,15	0.29	0	17,19,21	0.36	0
5	NAG	d	1	1,5	14,14,15	0.35	0	17,19,21	0.65	1 (5%)
5	NAG	d	2	5	14,14,15	0.23	0	17,19,21	0.40	0
5	BMA	d	3	5	11,11,12	0.58	0	15,15,17	1.00	1 (6%)
6	NAG	e	1	1,6	14,14,15	0.45	0	17,19,21	0.74	0
6	NAG	e	2	6	14,14,15	0.30	0	17,19,21	0.52	0
5	NAG	f	1	1,5	14,14,15	0.87	1 (7%)	17,19,21	0.54	0
5	NAG	f	2	5	14,14,15	0.71	1 (7%)	17,19,21	0.67	0
5	BMA	f	3	5	11,11,12	0.49	0	15,15,17	0.96	1 (6%)
5	NAG	g	1	1,5	14,14,15	1.41	2 (14%)	17,19,21	0.67	0
5	NAG	g	2	5	14,14,15	0.23	0	17,19,21	0.37	0

PRELIMINARY VALIDATION REPORT

Mol	Type	Chain	Res	Link	Bond lengths			Bond angles		
					Counts	RMSZ	#|Z| > 2	Counts	RMSZ	#|Z| > 2
5	BMA	g	3	5	11,11,12	0.55	0	15,15,17	0.84	1 (6%)
6	NAG	h	1	1,6	14,14,15	0.57	0	17,19,21	0.96	1 (5%)
6	NAG	h	2	6	14,14,15	0.23	0	17,19,21	0.61	0
6	NAG	i	1	1,6	14,14,15	0.81	1 (7%)	17,19,21	0.66	0
6	NAG	i	2	6	14,14,15	0.55	0	17,19,21	0.56	0
7	NAG	j	1	1,7	14,14,15	0.99	1 (7%)	17,19,21	0.47	0
7	NAG	j	2	7	14,14,15	0.78	1 (7%)	17,19,21	0.67	0
7	BMA	j	3	7	11,11,12	0.91	1 (9%)	15,15,17	0.85	0
7	MAN	j	4	7	11,11,12	0.75	0	15,15,17	1.35	2 (13%)
7	MAN	j	5	7	11,11,12	0.65	0	15,15,17	1.54	2 (13%)
7	MAN	j	6	7	11,11,12	0.63	0	15,15,17	0.95	1 (6%)
7	MAN	j	7	7	11,11,12	0.50	0	15,15,17	1.14	2 (13%)
5	NAG	k	1	1,5	14,14,15	0.50	0	17,19,21	0.34	0
5	NAG	k	2	5	14,14,15	0.22	0	17,19,21	0.44	0
5	BMA	k	3	5	11,11,12	0.50	0	15,15,17	0.83	0
8	NAG	l	1	1,8	14,14,15	0.47	0	17,19,21	0.51	0
8	NAG	l	2	8	14,14,15	0.31	0	17,19,21	0.47	0
8	BMA	l	3	8	11,11,12	0.61	0	15,15,17	0.70	0
8	MAN	l	4	8	11,11,12	0.48	0	15,15,17	1.06	2 (13%)
8	MAN	l	5	8	11,11,12	0.68	0	15,15,17	1.05	1 (6%)
9	NAG	m	1	1,9	14,14,15	0.29	0	17,19,21	1.11	1 (5%)
9	NAG	m	2	9	14,14,15	1.52	1 (7%)	17,19,21	1.12	1 (5%)
9	BMA	m	3	9	11,11,12	1.45	1 (9%)	15,15,17	1.36	2 (13%)
9	MAN	m	4	9	11,11,12	0.99	1 (9%)	15,15,17	1.17	1 (6%)
9	MAN	m	5	9	11,11,12	1.42	1 (9%)	15,15,17	1.91	4 (26%)
9	MAN	m	6	9	11,11,12	0.84	1 (9%)	15,15,17	0.92	1 (6%)
9	MAN	m	7	9	11,11,12	1.05	1 (9%)	15,15,17	1.12	1 (6%)
10	NAG	n	1	10,1	14,14,15	0.60	1 (7%)	17,19,21	0.52	0
10	NAG	n	2	10	14,14,15	0.87	1 (7%)	17,19,21	0.57	0
10	BMA	n	3	10	11,11,12	1.24	2 (18%)	15,15,17	1.35	3 (20%)
10	MAN	n	4	10	11,11,12	0.52	0	15,15,17	1.15	2 (13%)
10	MAN	n	5	10	11,11,12	1.04	1 (9%)	15,15,17	1.71	3 (20%)
10	MAN	n	6	10	11,11,12	2.81	7 (63%)	15,15,17	2.02	4 (26%)
10	MAN	n	7	10	11,11,12	0.72	0	15,15,17	0.93	1 (6%)
10	MAN	n	8	10	11,11,12	1.67	1 (9%)	15,15,17	1.42	2 (13%)
10	MAN	n	9	10	11,11,12	1.59	2 (18%)	15,15,17	1.56	3 (20%)

PRELIMINARY VALIDATION REPORT

Mol	Type	Chain	Res	Link	Bond lengths			Bond angles		
					Counts	RMSZ	#|Z| > 2	Counts	RMSZ	#|Z| > 2
6	NAG	o	1	1,6	14,14,15	0.72	1 (7%)	17,19,21	0.56	0
6	NAG	o	2	6	14,14,15	0.36	0	17,19,21	0.45	0
5	NAG	p	1	1,5	14,14,15	1.02	1 (7%)	17,19,21	0.72	0
5	NAG	p	2	5	14,14,15	0.36	0	17,19,21	0.47	0
5	BMA	p	3	5	11,11,12	0.85	1 (9%)	15,15,17	0.91	0
5	NAG	q	1	1,5	14,14,15	0.58	1 (7%)	17,19,21	0.51	0
5	NAG	q	2	5	14,14,15	0.22	0	17,19,21	0.53	0
5	BMA	q	3	5	11,11,12	0.65	0	15,15,17	0.97	1 (6%)
6	NAG	r	1	1,6	14,14,15	0.33	0	17,19,21	0.46	0
6	NAG	r	2	6	14,14,15	0.34	0	17,19,21	0.49	0
6	NAG	s	1	1,6	14,14,15	0.79	1 (7%)	17,19,21	0.68	0
6	NAG	s	2	6	14,14,15	0.25	0	17,19,21	0.72	1 (5%)
6	NAG	t	1	2,6	14,14,15	0.40	0	17,19,21	0.40	0
6	NAG	t	2	6	14,14,15	0.30	0	17,19,21	0.36	0
5	NAG	u	1	1,5	14,14,15	0.35	0	17,19,21	0.64	0
5	NAG	u	2	5	14,14,15	0.22	0	17,19,21	0.40	0
5	BMA	u	3	5	11,11,12	0.59	0	15,15,17	1.01	1 (6%)
6	NAG	v	1	1,6	14,14,15	0.45	0	17,19,21	0.73	0
6	NAG	v	2	6	14,14,15	0.28	0	17,19,21	0.52	0
5	NAG	w	1	1,5	14,14,15	0.86	1 (7%)	17,19,21	0.54	0
5	NAG	w	2	5	14,14,15	0.69	1 (7%)	17,19,21	0.67	0
5	BMA	w	3	5	11,11,12	0.48	0	15,15,17	0.96	1 (6%)
5	NAG	x	1	1,5	14,14,15	1.41	2 (14%)	17,19,21	0.66	0
5	NAG	x	2	5	14,14,15	0.24	0	17,19,21	0.37	0
5	BMA	x	3	5	11,11,12	0.56	0	15,15,17	0.85	1 (6%)
6	NAG	y	1	1,6	14,14,15	0.57	0	17,19,21	0.96	1 (5%)
6	NAG	y	2	6	14,14,15	0.21	0	17,19,21	0.62	0
6	NAG	z	1	1,6	14,14,15	0.81	1 (7%)	17,19,21	0.67	0
6	NAG	z	2	6	14,14,15	0.56	0	17,19,21	0.57	0

In the following table, the Chirals column lists the number of chiral outliers, the number of chiral centers analysed, the number of these observed in the model and the number defined in the Chemical Component Dictionary. Similar counts are reported in the Torsion and Rings columns. '-' means no outliers of that kind were identified.

Mol	Type	Chain	Res	Link	Chirals	Torsions	Rings
7	NAG	0	1	1,7	-	3/6/23/26	0/1/1/1
7	NAG	0	2	7	-	3/6/23/26	0/1/1/1
7	BMA	0	3	7	-	2/2/19/22	0/1/1/1

Continued on next page...

PRELIMINARY VALIDATION REPORT

Continued from previous page...

Mol	Type	Chain	Res	Link	Chirals	Torsions	Rings
7	MAN	0	4	7	-	2/2/19/22	0/1/1/1
7	MAN	0	5	7	-	1/2/19/22	0/1/1/1
7	MAN	0	6	7	-	0/2/19/22	0/1/1/1
7	MAN	0	7	7	-	2/2/19/22	0/1/1/1
5	NAG	1	1	1,5	-	1/6/23/26	0/1/1/1
5	NAG	1	2	5	-	2/6/23/26	0/1/1/1
5	BMA	1	3	5	-	2/2/19/22	0/1/1/1
8	NAG	2	1	1,8	-	2/6/23/26	0/1/1/1
8	NAG	2	2	8	-	4/6/23/26	0/1/1/1
8	BMA	2	3	8	-	0/2/19/22	0/1/1/1
8	MAN	2	4	8	-	2/2/19/22	0/1/1/1
8	MAN	2	5	8	-	1/2/19/22	0/1/1/1
9	NAG	3	1	1,9	-	2/6/23/26	0/1/1/1
9	NAG	3	2	9	-	3/6/23/26	0/1/1/1
9	BMA	3	3	9	-	2/2/19/22	0/1/1/1
9	MAN	3	4	9	-	1/2/19/22	0/1/1/1
9	MAN	3	5	9	-	0/2/19/22	0/1/1/1
9	MAN	3	6	9	-	1/2/19/22	0/1/1/1
9	MAN	3	7	9	-	1/2/19/22	0/1/1/1
10	NAG	4	1	10,1	-	3/6/23/26	0/1/1/1
10	NAG	4	2	10	-	2/6/23/26	0/1/1/1
10	BMA	4	3	10	-	0/2/19/22	0/1/1/1
10	MAN	4	4	10	-	0/2/19/22	0/1/1/1
10	MAN	4	5	10	-	0/2/19/22	0/1/1/1
10	MAN	4	6	10	-	2/2/19/22	0/1/1/1
10	MAN	4	7	10	-	1/2/19/22	0/1/1/1
10	MAN	4	8	10	-	1/2/19/22	0/1/1/1
10	MAN	4	9	10	-	0/2/19/22	0/1/1/1
6	NAG	5	1	1,6	-	2/6/23/26	0/1/1/1
6	NAG	5	2	6	-	2/6/23/26	0/1/1/1
5	NAG	6	1	1,5	-	0/6/23/26	0/1/1/1
5	NAG	6	2	5	-	1/6/23/26	0/1/1/1
5	BMA	6	3	5	-	0/2/19/22	0/1/1/1
5	NAG	7	1	1,5	-	0/6/23/26	0/1/1/1
5	NAG	7	2	5	-	0/6/23/26	0/1/1/1
5	BMA	7	3	5	-	0/2/19/22	0/1/1/1
6	NAG	8	1	1,6	-	0/6/23/26	0/1/1/1
6	NAG	8	2	6	-	3/6/23/26	0/1/1/1

Continued on next page...

PRELIMINARY VALIDATION REPORT

Continued from previous page...

Mol	Type	Chain	Res	Link	Chirals	Torsions	Rings
6	NAG	9	1	1,6	-	4/6/23/26	0/1/1/1
6	NAG	9	2	6	-	2/6/23/26	0/1/1/1
6	NAG	AA	1	2,6	-	3/6/23/26	0/1/1/1
6	NAG	AA	2	6	-	0/6/23/26	0/1/1/1
5	NAG	M	1	1,5	-	2/6/23/26	0/1/1/1
5	NAG	M	2	5	-	0/6/23/26	0/1/1/1
5	BMA	M	3	5	-	2/2/19/22	0/1/1/1
6	NAG	N	1	1,6	-	2/6/23/26	0/1/1/1
6	NAG	N	2	6	-	2/6/23/26	0/1/1/1
5	NAG	O	1	1,5	-	0/6/23/26	0/1/1/1
5	NAG	O	2	5	-	2/6/23/26	0/1/1/1
5	BMA	O	3	5	-	2/2/19/22	0/1/1/1
5	NAG	P	1	1,5	-	2/6/23/26	0/1/1/1
5	NAG	P	2	5	-	0/6/23/26	0/1/1/1
5	BMA	P	3	5	-	2/2/19/22	0/1/1/1
6	NAG	Q	1	1,6	-	1/6/23/26	0/1/1/1
6	NAG	Q	2	6	-	0/6/23/26	0/1/1/1
6	NAG	R	1	1,6	-	2/6/23/26	0/1/1/1
6	NAG	R	2	6	-	0/6/23/26	0/1/1/1
7	NAG	S	1	1,7	-	3/6/23/26	0/1/1/1
7	NAG	S	2	7	-	3/6/23/26	0/1/1/1
7	BMA	S	3	7	-	2/2/19/22	0/1/1/1
7	MAN	S	4	7	-	2/2/19/22	0/1/1/1
7	MAN	S	5	7	-	1/2/19/22	0/1/1/1
7	MAN	S	6	7	-	0/2/19/22	0/1/1/1
7	MAN	S	7	7	-	2/2/19/22	0/1/1/1
5	NAG	T	1	1,5	-	1/6/23/26	0/1/1/1
5	NAG	T	2	5	-	2/6/23/26	0/1/1/1
5	BMA	T	3	5	-	2/2/19/22	0/1/1/1
8	NAG	U	1	1,8	-	2/6/23/26	0/1/1/1
8	NAG	U	2	8	-	4/6/23/26	0/1/1/1
8	BMA	U	3	8	-	0/2/19/22	0/1/1/1
8	MAN	U	4	8	-	2/2/19/22	0/1/1/1
8	MAN	U	5	8	-	1/2/19/22	0/1/1/1
9	NAG	V	1	1,9	-	2/6/23/26	0/1/1/1
9	NAG	V	2	9	-	3/6/23/26	0/1/1/1

Continued on next page...

PRELIMINARY VALIDATION REPORT

Continued from previous page...

Mol	Type	Chain	Res	Link	Chirals	Torsions	Rings
9	BMA	V	3	9	-	2/2/19/22	0/1/1/1
9	MAN	V	4	9	-	1/2/19/22	0/1/1/1
9	MAN	V	5	9	-	0/2/19/22	0/1/1/1
9	MAN	V	6	9	-	1/2/19/22	0/1/1/1
9	MAN	V	7	9	-	1/2/19/22	0/1/1/1
10	NAG	W	1	10,1	-	3/6/23/26	0/1/1/1
10	NAG	W	2	10	-	2/6/23/26	0/1/1/1
10	BMA	W	3	10	-	0/2/19/22	0/1/1/1
10	MAN	W	4	10	-	0/2/19/22	0/1/1/1
10	MAN	W	5	10	-	0/2/19/22	0/1/1/1
10	MAN	W	6	10	-	2/2/19/22	0/1/1/1
10	MAN	W	7	10	-	1/2/19/22	0/1/1/1
10	MAN	W	8	10	-	1/2/19/22	0/1/1/1
10	MAN	W	9	10	-	0/2/19/22	0/1/1/1
6	NAG	X	1	1,6	-	2/6/23/26	0/1/1/1
6	NAG	X	2	6	-	2/6/23/26	0/1/1/1
5	NAG	Y	1	1,5	-	0/6/23/26	0/1/1/1
5	NAG	Y	2	5	-	1/6/23/26	0/1/1/1
5	BMA	Y	3	5	-	0/2/19/22	0/1/1/1
5	NAG	Z	1	1,5	-	0/6/23/26	0/1/1/1
5	NAG	Z	2	5	-	0/6/23/26	0/1/1/1
5	BMA	Z	3	5	-	0/2/19/22	0/1/1/1
6	NAG	a	1	1,6	-	0/6/23/26	0/1/1/1
6	NAG	a	2	6	-	3/6/23/26	0/1/1/1
6	NAG	b	1	1,6	-	4/6/23/26	0/1/1/1
6	NAG	b	2	6	-	2/6/23/26	0/1/1/1
6	NAG	c	1	2,6	-	3/6/23/26	0/1/1/1
6	NAG	c	2	6	-	0/6/23/26	0/1/1/1
5	NAG	d	1	1,5	-	2/6/23/26	0/1/1/1
5	NAG	d	2	5	-	0/6/23/26	0/1/1/1
5	BMA	d	3	5	-	2/2/19/22	0/1/1/1
6	NAG	e	1	1,6	-	2/6/23/26	0/1/1/1
6	NAG	e	2	6	-	2/6/23/26	0/1/1/1
5	NAG	f	1	1,5	-	0/6/23/26	0/1/1/1
5	NAG	f	2	5	-	2/6/23/26	0/1/1/1
5	BMA	f	3	5	-	2/2/19/22	0/1/1/1
5	NAG	g	1	1,5	-	2/6/23/26	0/1/1/1
5	NAG	g	2	5	-	0/6/23/26	0/1/1/1

Continued on next page...

PRELIMINARY VALIDATION REPORT

Continued from previous page...

Mol	Type	Chain	Res	Link	Chirals	Torsions	Rings
5	BMA	g	3	5	-	2/2/19/22	0/1/1/1
6	NAG	h	1	1,6	-	1/6/23/26	0/1/1/1
6	NAG	h	2	6	-	0/6/23/26	0/1/1/1
6	NAG	i	1	1,6	-	2/6/23/26	0/1/1/1
6	NAG	i	2	6	-	0/6/23/26	0/1/1/1
7	NAG	j	1	1,7	-	3/6/23/26	0/1/1/1
7	NAG	j	2	7	-	3/6/23/26	0/1/1/1
7	BMA	j	3	7	-	2/2/19/22	0/1/1/1
7	MAN	j	4	7	-	2/2/19/22	0/1/1/1
7	MAN	j	5	7	-	1/2/19/22	0/1/1/1
7	MAN	j	6	7	-	0/2/19/22	0/1/1/1
7	MAN	j	7	7	-	2/2/19/22	0/1/1/1
5	NAG	k	1	1,5	-	1/6/23/26	0/1/1/1
5	NAG	k	2	5	-	2/6/23/26	0/1/1/1
5	BMA	k	3	5	-	2/2/19/22	0/1/1/1
8	NAG	l	1	1,8	-	2/6/23/26	0/1/1/1
8	NAG	l	2	8	-	4/6/23/26	0/1/1/1
8	BMA	l	3	8	-	0/2/19/22	0/1/1/1
8	MAN	l	4	8	-	2/2/19/22	0/1/1/1
8	MAN	l	5	8	-	1/2/19/22	0/1/1/1
9	NAG	m	1	1,9	-	2/6/23/26	0/1/1/1
9	NAG	m	2	9	-	3/6/23/26	0/1/1/1
9	BMA	m	3	9	-	2/2/19/22	0/1/1/1
9	MAN	m	4	9	-	1/2/19/22	0/1/1/1
9	MAN	m	5	9	-	0/2/19/22	0/1/1/1
9	MAN	m	6	9	-	1/2/19/22	0/1/1/1
9	MAN	m	7	9	-	1/2/19/22	0/1/1/1
10	NAG	n	1	10,1	-	3/6/23/26	0/1/1/1
10	NAG	n	2	10	-	2/6/23/26	0/1/1/1
10	BMA	n	3	10	-	0/2/19/22	0/1/1/1
10	MAN	n	4	10	-	0/2/19/22	0/1/1/1
10	MAN	n	5	10	-	0/2/19/22	0/1/1/1
10	MAN	n	6	10	-	2/2/19/22	0/1/1/1
10	MAN	n	7	10	-	1/2/19/22	0/1/1/1
10	MAN	n	8	10	-	1/2/19/22	0/1/1/1
10	MAN	n	9	10	-	0/2/19/22	0/1/1/1
6	NAG	o	1	1,6	-	2/6/23/26	0/1/1/1
6	NAG	o	2	6	-	2/6/23/26	0/1/1/1

Continued on next page...

PRELIMINARY VALIDATION REPORT

Continued from previous page...

Mol	Type	Chain	Res	Link	Chirals	Torsions	Rings
5	NAG	p	1	1,5	-	0/6/23/26	0/1/1/1
5	NAG	p	2	5	-	1/6/23/26	0/1/1/1
5	BMA	p	3	5	-	0/2/19/22	0/1/1/1
5	NAG	q	1	1,5	-	0/6/23/26	0/1/1/1
5	NAG	q	2	5	-	0/6/23/26	0/1/1/1
5	BMA	q	3	5	-	0/2/19/22	0/1/1/1
6	NAG	r	1	1,6	-	0/6/23/26	0/1/1/1
6	NAG	r	2	6	-	3/6/23/26	0/1/1/1
6	NAG	s	1	1,6	-	4/6/23/26	0/1/1/1
6	NAG	s	2	6	-	2/6/23/26	0/1/1/1
6	NAG	t	1	2,6	-	3/6/23/26	0/1/1/1
6	NAG	t	2	6	-	0/6/23/26	0/1/1/1
5	NAG	u	1	1,5	-	2/6/23/26	0/1/1/1
5	NAG	u	2	5	-	0/6/23/26	0/1/1/1
5	BMA	u	3	5	-	2/2/19/22	0/1/1/1
6	NAG	v	1	1,6	-	2/6/23/26	0/1/1/1
6	NAG	v	2	6	-	2/6/23/26	0/1/1/1
5	NAG	w	1	1,5	-	0/6/23/26	0/1/1/1
5	NAG	w	2	5	-	2/6/23/26	0/1/1/1
5	BMA	w	3	5	-	2/2/19/22	0/1/1/1
5	NAG	x	1	1,5	-	2/6/23/26	0/1/1/1
5	NAG	x	2	5	-	0/6/23/26	0/1/1/1
5	BMA	x	3	5	-	2/2/19/22	0/1/1/1
6	NAG	y	1	1,6	-	1/6/23/26	0/1/1/1
6	NAG	y	2	6	-	0/6/23/26	0/1/1/1
6	NAG	z	1	1,6	-	2/6/23/26	0/1/1/1
6	NAG	z	2	6	-	0/6/23/26	0/1/1/1

All (103) bond length outliers are listed below:

Mol	Chain	Res	Type	Atoms	Z	Observed(Å)	Ideal(Å)
9	m	2	NAG	C1-C2	5.24	1.60	1.52
9	V	2	NAG	C1-C2	5.23	1.60	1.52
9	3	2	NAG	C1-C2	5.19	1.60	1.52
5	g	1	NAG	O5-C1	-4.77	1.36	1.43
5	x	1	NAG	O5-C1	-4.77	1.36	1.43
5	P	1	NAG	O5-C1	-4.70	1.36	1.43
10	4	6	MAN	O3-C3	-4.66	1.32	1.43
10	n	6	MAN	O3-C3	-4.62	1.32	1.43

Continued on next page...

PRELIMINARY VALIDATION REPORT

Continued from previous page...

Mol	Chain	Res	Type	Atoms	Z	Observed(Å)	Ideal(Å)
10	W	6	MAN	O3-C3	-4.60	1.32	1.43
10	W	8	MAN	O5-C1	-4.09	1.37	1.43
10	n	8	MAN	O5-C1	-4.06	1.37	1.43
10	4	8	MAN	O5-C1	-4.06	1.37	1.43
10	4	6	MAN	C1-C2	3.69	1.60	1.52
10	n	6	MAN	O2-C2	-3.67	1.35	1.43
10	n	6	MAN	C1-C2	3.67	1.60	1.52
10	W	6	MAN	C1-C2	3.66	1.60	1.52
10	4	6	MAN	O2-C2	-3.65	1.35	1.43
10	W	6	MAN	O2-C2	-3.63	1.35	1.43
10	4	6	MAN	O6-C6	3.61	1.57	1.42
10	n	9	MAN	O5-C1	-3.59	1.38	1.43
10	n	6	MAN	O6-C6	3.59	1.57	1.42
10	W	6	MAN	O6-C6	3.58	1.57	1.42
10	W	9	MAN	O5-C1	-3.58	1.38	1.43
10	4	9	MAN	O5-C1	-3.58	1.38	1.43
7	S	1	NAG	O5-C1	-3.53	1.38	1.43
7	0	1	NAG	O5-C1	-3.52	1.38	1.43
7	j	1	NAG	O5-C1	-3.51	1.38	1.43
5	Y	1	NAG	O5-C1	-3.40	1.38	1.43
5	6	1	NAG	O5-C1	-3.37	1.38	1.43
5	p	1	NAG	O5-C1	-3.36	1.38	1.43
5	f	1	NAG	O5-C1	-3.17	1.38	1.43
10	n	2	NAG	O5-C1	-3.16	1.38	1.43
10	W	2	NAG	O5-C1	-3.16	1.38	1.43
10	4	2	NAG	O5-C1	-3.13	1.38	1.43
5	w	1	NAG	O5-C1	-3.12	1.38	1.43
10	W	9	MAN	C2-C3	-3.11	1.47	1.52
5	O	1	NAG	O5-C1	-3.09	1.38	1.43
10	4	9	MAN	C2-C3	-3.08	1.48	1.52
10	n	9	MAN	C2-C3	-3.06	1.48	1.52
9	3	5	MAN	C1-C2	3.06	1.59	1.52
9	m	5	MAN	C1-C2	3.06	1.59	1.52
9	V	5	MAN	C1-C2	3.06	1.59	1.52
10	n	6	MAN	O4-C4	-2.95	1.36	1.43
10	4	6	MAN	O4-C4	-2.93	1.36	1.43
10	W	6	MAN	O4-C4	-2.90	1.36	1.43
6	z	1	NAG	O5-C1	-2.88	1.39	1.43
6	b	1	NAG	O5-C1	-2.87	1.39	1.43
6	9	1	NAG	O5-C1	-2.86	1.39	1.43
6	i	1	NAG	O5-C1	-2.86	1.39	1.43
6	s	1	NAG	O5-C1	-2.84	1.39	1.43

Continued on next page...

PRELIMINARY VALIDATION REPORT

Continued from previous page...

Mol	Chain	Res	Type	Atoms	Z	Observed(Å)	Ideal(Å)
6	R	1	NAG	O5-C1	-2.84	1.39	1.43
9	3	3	BMA	C1-C2	2.75	1.58	1.52
9	V	3	BMA	C1-C2	2.75	1.58	1.52
9	m	3	BMA	C1-C2	2.74	1.58	1.52
10	n	5	MAN	O5-C5	2.73	1.49	1.43
10	W	5	MAN	O5-C5	2.69	1.48	1.43
10	4	5	MAN	O5-C5	2.66	1.48	1.43
6	5	1	NAG	O5-C1	-2.58	1.39	1.43
10	W	3	BMA	O5-C1	-2.58	1.39	1.43
10	n	3	BMA	O5-C1	-2.58	1.39	1.43
6	o	1	NAG	O5-C1	-2.56	1.39	1.43
10	4	3	BMA	O5-C1	-2.56	1.39	1.43
6	X	1	NAG	O5-C1	-2.55	1.39	1.43
5	f	2	NAG	O5-C1	-2.54	1.39	1.43
7	j	2	NAG	O5-C1	-2.52	1.39	1.43
10	W	6	MAN	C6-C5	2.50	1.60	1.51
10	n	6	MAN	C6-C5	2.50	1.60	1.51
10	W	6	MAN	C4-C5	-2.50	1.47	1.53
9	3	4	MAN	O5-C1	-2.49	1.39	1.43
5	O	2	NAG	O5-C1	-2.49	1.39	1.43
9	V	4	MAN	O5-C1	-2.48	1.39	1.43
10	4	6	MAN	C6-C5	2.48	1.60	1.51
7	0	2	NAG	O5-C1	-2.48	1.39	1.43
9	m	4	MAN	O5-C1	-2.47	1.39	1.43
5	w	2	NAG	O5-C1	-2.47	1.39	1.43
10	n	6	MAN	C4-C5	-2.47	1.47	1.53
7	S	2	NAG	O5-C1	-2.46	1.39	1.43
10	4	6	MAN	C4-C5	-2.43	1.47	1.53
9	m	7	MAN	O5-C1	-2.30	1.40	1.43
9	3	7	MAN	O5-C1	-2.30	1.40	1.43
9	V	7	MAN	O5-C1	-2.28	1.40	1.43
7	j	3	BMA	O5-C1	-2.27	1.40	1.43
7	S	3	BMA	O5-C1	-2.27	1.40	1.43
5	p	3	BMA	C1-C2	2.26	1.57	1.52
5	6	3	BMA	C1-C2	2.24	1.57	1.52
5	Y	3	BMA	C1-C2	2.24	1.57	1.52
7	0	3	BMA	O5-C1	-2.23	1.40	1.43
9	m	6	MAN	O5-C1	-2.20	1.40	1.43
9	3	6	MAN	O5-C1	-2.20	1.40	1.43
9	V	6	MAN	O5-C1	-2.17	1.40	1.43
10	4	1	NAG	O5-C1	-2.17	1.40	1.43
10	W	1	NAG	O5-C1	-2.15	1.40	1.43

Continued on next page...

PRELIMINARY VALIDATION REPORT

Continued from previous page...

Mol	Chain	Res	Type	Atoms	Z	Observed(Å)	Ideal(Å)
10	n	1	NAG	O5-C1	-2.11	1.40	1.43
5	q	1	NAG	O5-C1	-2.10	1.40	1.43
5	g	1	NAG	C1-C2	-2.07	1.49	1.52
5	7	1	NAG	O5-C1	-2.06	1.40	1.43
5	Z	1	NAG	O5-C1	-2.06	1.40	1.43
5	P	1	NAG	C1-C2	-2.05	1.49	1.52
5	x	1	NAG	C1-C2	-2.04	1.49	1.52
6	R	2	NAG	C1-C2	2.03	1.55	1.52
10	W	3	BMA	O3-C3	-2.02	1.38	1.43
10	4	3	BMA	O3-C3	-2.01	1.38	1.43
10	n	3	BMA	O3-C3	-2.01	1.38	1.43

All (139) bond angle outliers are listed below:

Mol	Chain	Res	Type	Atoms	Z	Observed(^{o})	Ideal(^{o})
10	n	6	MAN	C3-C4-C5	-4.93	101.45	110.24
10	4	6	MAN	C3-C4-C5	-4.92	101.46	110.24
10	W	6	MAN	C3-C4-C5	-4.92	101.47	110.24
7	j	5	MAN	C1-O5-C5	4.80	118.70	112.19
7	S	5	MAN	C1-O5-C5	4.78	118.67	112.19
7	0	5	MAN	C1-O5-C5	4.77	118.66	112.19
10	4	5	MAN	C1-O5-C5	4.22	117.90	112.19
10	W	5	MAN	C1-O5-C5	4.21	117.90	112.19
10	n	5	MAN	C1-O5-C5	4.20	117.88	112.19
9	3	5	MAN	C1-C2-C3	3.85	114.40	109.67
9	3	5	MAN	C1-O5-C5	3.81	117.36	112.19
9	V	5	MAN	C1-C2-C3	3.81	114.35	109.67
9	m	5	MAN	C1-C2-C3	3.81	114.35	109.67
9	m	5	MAN	C1-O5-C5	3.79	117.33	112.19
9	V	5	MAN	C1-O5-C5	3.78	117.31	112.19
10	n	4	MAN	C1-O5-C5	3.57	117.03	112.19
10	W	4	MAN	C1-O5-C5	3.54	116.99	112.19
10	4	4	MAN	C1-O5-C5	3.54	116.98	112.19
9	V	1	NAG	C1-O5-C5	3.41	116.81	112.19
9	m	1	NAG	C1-O5-C5	3.39	116.79	112.19
9	3	1	NAG	C1-O5-C5	3.39	116.78	112.19
9	V	3	BMA	C1-C2-C3	3.23	113.64	109.67
9	3	3	BMA	C1-C2-C3	3.23	113.63	109.67
9	m	3	BMA	C1-C2-C3	3.19	113.59	109.67
10	n	6	MAN	O5-C1-C2	3.19	115.69	110.77
10	W	6	MAN	O5-C1-C2	3.18	115.68	110.77
10	4	6	MAN	O5-C1-C2	3.17	115.66	110.77

Continued on next page...

PRELIMINARY VALIDATION REPORT

Continued from previous page...

Mol	Chain	Res	Type	Atoms	Z	Observed(°)	Ideal(°)
9	m	5	MAN	O2-C2-C3	-3.11	103.91	110.14
9	V	5	MAN	O2-C2-C3	-3.10	103.94	110.14
9	3	5	MAN	O2-C2-C3	-3.09	103.94	110.14
7	S	7	MAN	C1-O5-C5	3.07	116.35	112.19
7	j	7	MAN	C1-O5-C5	3.06	116.34	112.19
10	W	9	MAN	C2-C3-C4	-3.06	105.60	110.89
7	0	7	MAN	C1-O5-C5	3.06	116.33	112.19
10	n	9	MAN	C2-C3-C4	-3.04	105.64	110.89
10	4	9	MAN	C2-C3-C4	-3.04	105.64	110.89
10	n	5	MAN	C1-C2-C3	-3.03	105.94	109.67
10	W	5	MAN	C1-C2-C3	-3.03	105.95	109.67
7	j	4	MAN	C1-O5-C5	3.02	116.29	112.19
10	4	5	MAN	C1-C2-C3	-3.02	105.96	109.67
7	0	4	MAN	C1-O5-C5	3.00	116.25	112.19
10	n	9	MAN	O2-C2-C3	-2.98	104.18	110.14
10	4	9	MAN	O2-C2-C3	-2.96	104.20	110.14
10	W	9	MAN	O2-C2-C3	-2.95	104.22	110.14
7	S	4	MAN	C1-O5-C5	2.95	116.19	112.19
10	W	3	BMA	C2-C3-C4	2.91	115.93	110.89
10	4	3	BMA	C2-C3-C4	2.91	115.93	110.89
10	n	3	BMA	C2-C3-C4	2.90	115.91	110.89
9	m	4	MAN	C1-O5-C5	2.85	116.05	112.19
9	3	4	MAN	C1-O5-C5	2.83	116.03	112.19
9	V	4	MAN	C1-O5-C5	2.82	116.02	112.19
9	V	2	NAG	C3-C4-C5	-2.79	105.26	110.24
9	3	2	NAG	C3-C4-C5	-2.76	105.31	110.24
9	m	2	NAG	C3-C4-C5	-2.76	105.31	110.24
10	4	8	MAN	C3-C4-C5	-2.71	105.41	110.24
10	W	8	MAN	C3-C4-C5	-2.70	105.43	110.24
10	n	8	MAN	C3-C4-C5	-2.70	105.43	110.24
9	m	5	MAN	O5-C1-C2	2.67	114.90	110.77
9	m	6	MAN	O2-C2-C3	-2.67	104.79	110.14
9	V	5	MAN	O5-C1-C2	2.66	114.88	110.77
9	3	6	MAN	O2-C2-C3	-2.65	104.83	110.14
9	V	6	MAN	O2-C2-C3	-2.64	104.84	110.14
9	3	5	MAN	O5-C1-C2	2.64	114.84	110.77
10	4	5	MAN	O3-C3-C2	2.62	115.01	109.99
10	n	5	MAN	O3-C3-C2	2.61	114.99	109.99
10	4	9	MAN	C1-C2-C3	-2.60	106.47	109.67
10	W	5	MAN	O3-C3-C2	2.59	114.95	109.99
10	n	9	MAN	C1-C2-C3	-2.57	106.51	109.67
10	4	6	MAN	O2-C2-C3	-2.56	105.00	110.14

Continued on next page...

PRELIMINARY VALIDATION REPORT

Continued from previous page...

Mol	Chain	Res	Type	Atoms	Z	Observed(°)	Ideal(°)
10	W	9	MAN	C1-C2-C3	-2.56	106.52	109.67
10	n	6	MAN	O2-C2-C3	-2.56	105.02	110.14
10	W	6	MAN	O2-C2-C3	-2.55	105.03	110.14
6	s	2	NAG	C1-O5-C5	2.55	115.65	112.19
10	4	7	MAN	O2-C2-C3	-2.54	105.04	110.14
8	2	4	MAN	C1-O5-C5	2.54	115.64	112.19
6	b	2	NAG	C1-O5-C5	2.54	115.63	112.19
7	j	4	MAN	O2-C2-C3	-2.52	105.09	110.14
10	W	7	MAN	O2-C2-C3	-2.51	105.11	110.14
10	n	8	MAN	O2-C2-C3	-2.51	105.11	110.14
10	4	8	MAN	O2-C2-C3	-2.51	105.11	110.14
10	W	8	MAN	O2-C2-C3	-2.51	105.11	110.14
10	n	7	MAN	O2-C2-C3	-2.51	105.11	110.14
8	U	4	MAN	C1-O5-C5	2.51	115.59	112.19
7	S	7	MAN	O2-C2-C3	-2.51	105.12	110.14
7	0	4	MAN	O2-C2-C3	-2.50	105.12	110.14
6	9	2	NAG	C1-O5-C5	2.50	115.58	112.19
7	j	7	MAN	O2-C2-C3	-2.50	105.13	110.14
7	0	7	MAN	O2-C2-C3	-2.50	105.14	110.14
8	l	4	MAN	C1-O5-C5	2.49	115.57	112.19
7	S	4	MAN	O2-C2-C3	-2.49	105.14	110.14
6	Q	1	NAG	C1-O5-C5	2.41	115.45	112.19
10	4	6	MAN	C1-O5-C5	2.39	115.43	112.19
10	n	6	MAN	C1-O5-C5	2.39	115.43	112.19
6	h	1	NAG	C1-O5-C5	2.38	115.42	112.19
6	y	1	NAG	C1-O5-C5	2.38	115.41	112.19
9	m	7	MAN	O2-C2-C3	-2.36	105.40	110.14
10	W	6	MAN	C1-O5-C5	2.36	115.39	112.19
9	V	7	MAN	O2-C2-C3	-2.36	105.41	110.14
9	3	7	MAN	O2-C2-C3	-2.35	105.44	110.14
5	7	3	BMA	C1-O5-C5	2.34	115.37	112.19
8	U	4	MAN	O2-C2-C3	-2.34	105.46	110.14
5	q	3	BMA	C1-O5-C5	2.33	115.35	112.19
7	0	5	MAN	O2-C2-C3	-2.33	105.47	110.14
9	3	3	BMA	O6-C6-C5	-2.33	103.31	111.29
9	V	3	BMA	O6-C6-C5	-2.33	103.31	111.29
7	S	5	MAN	O2-C2-C3	-2.33	105.48	110.14
8	2	4	MAN	O2-C2-C3	-2.32	105.48	110.14
9	m	3	BMA	O6-C6-C5	-2.32	103.33	111.29
5	Z	3	BMA	C1-O5-C5	2.32	115.33	112.19
7	j	5	MAN	O2-C2-C3	-2.32	105.50	110.14
8	l	4	MAN	O2-C2-C3	-2.32	105.50	110.14

Continued on next page...

PRELIMINARY VALIDATION REPORT

Continued from previous page...

Mol	Chain	Res	Type	Atoms	Z	Observed(°)	Ideal(°)
7	0	6	MAN	O2-C2-C3	-2.24	105.66	110.14
7	j	6	MAN	O2-C2-C3	-2.20	105.72	110.14
7	S	6	MAN	O2-C2-C3	-2.19	105.76	110.14
10	W	3	BMA	C3-C4-C5	2.11	114.00	110.24
10	n	3	BMA	O2-C2-C3	-2.09	105.96	110.14
10	4	3	BMA	C3-C4-C5	2.09	113.96	110.24
5	g	3	BMA	O2-C2-C3	-2.08	105.96	110.14
10	W	3	BMA	O2-C2-C3	-2.08	105.96	110.14
10	4	3	BMA	O2-C2-C3	-2.08	105.96	110.14
10	n	3	BMA	C3-C4-C5	2.08	113.95	110.24
5	x	3	BMA	O2-C2-C3	-2.08	105.97	110.14
10	4	4	MAN	O2-C2-C3	-2.08	105.97	110.14
5	P	3	BMA	O2-C2-C3	-2.08	105.98	110.14
5	u	3	BMA	C1-O5-C5	2.08	115.01	112.19
5	w	3	BMA	O2-C2-C3	-2.07	106.00	110.14
5	M	3	BMA	C1-O5-C5	2.06	114.99	112.19
10	n	4	MAN	O2-C2-C3	-2.06	106.01	110.14
5	d	1	NAG	C1-O5-C5	2.06	114.98	112.19
10	W	4	MAN	O2-C2-C3	-2.06	106.02	110.14
8	U	5	MAN	O2-C2-C3	-2.06	106.02	110.14
5	d	3	BMA	C1-O5-C5	2.05	114.97	112.19
5	f	3	BMA	O2-C2-C3	-2.05	106.03	110.14
5	O	3	BMA	O2-C2-C3	-2.05	106.04	110.14
8	2	5	MAN	O2-C2-C3	-2.05	106.04	110.14
8	l	5	MAN	O2-C2-C3	-2.04	106.04	110.14
10	4	8	MAN	O5-C5-C4	-2.02	105.92	110.83
10	W	8	MAN	O5-C5-C4	-2.01	105.93	110.83
5	M	1	NAG	C1-O5-C5	2.00	114.91	112.19

There are no chirality outliers.

All (246) torsion outliers are listed below:

Mol	Chain	Res	Type	Atoms
6	X	1	NAG	O5-C5-C6-O6
6	o	1	NAG	O5-C5-C6-O6
6	5	1	NAG	O5-C5-C6-O6
10	W	6	MAN	O5-C5-C6-O6
10	n	6	MAN	O5-C5-C6-O6
10	4	6	MAN	O5-C5-C6-O6
6	X	2	NAG	O5-C5-C6-O6
6	o	2	NAG	O5-C5-C6-O6
6	5	2	NAG	O5-C5-C6-O6

Continued on next page...

PRELIMINARY VALIDATION REPORT

Continued from previous page...

Mol	Chain	Res	Type	Atoms
6	N	1	NAG	O5-C5-C6-O6
6	c	1	NAG	O5-C5-C6-O6
6	e	1	NAG	O5-C5-C6-O6
6	t	1	NAG	O5-C5-C6-O6
6	v	1	NAG	O5-C5-C6-O6
6	AA	1	NAG	O5-C5-C6-O6
7	S	4	MAN	O5-C5-C6-O6
7	j	4	MAN	O5-C5-C6-O6
7	0	4	MAN	O5-C5-C6-O6
5	M	1	NAG	O5-C5-C6-O6
5	d	1	NAG	O5-C5-C6-O6
5	u	1	NAG	O5-C5-C6-O6
6	b	1	NAG	O5-C5-C6-O6
6	s	1	NAG	O5-C5-C6-O6
6	9	1	NAG	O5-C5-C6-O6
6	X	2	NAG	C4-C5-C6-O6
6	o	2	NAG	C4-C5-C6-O6
6	5	2	NAG	C4-C5-C6-O6
5	O	2	NAG	O5-C5-C6-O6
5	f	2	NAG	O5-C5-C6-O6
5	k	2	NAG	O5-C5-C6-O6
5	w	2	NAG	O5-C5-C6-O6
5	1	2	NAG	O5-C5-C6-O6
6	N	2	NAG	O5-C5-C6-O6
6	a	2	NAG	O5-C5-C6-O6
6	e	2	NAG	O5-C5-C6-O6
6	r	2	NAG	O5-C5-C6-O6
6	v	2	NAG	O5-C5-C6-O6
6	8	2	NAG	O5-C5-C6-O6
10	W	1	NAG	O5-C5-C6-O6
10	n	1	NAG	O5-C5-C6-O6
10	4	1	NAG	O5-C5-C6-O6
5	T	2	NAG	O5-C5-C6-O6
6	X	1	NAG	C4-C5-C6-O6
6	o	1	NAG	C4-C5-C6-O6
6	5	1	NAG	C4-C5-C6-O6
8	U	4	MAN	C4-C5-C6-O6
8	l	4	MAN	C4-C5-C6-O6
8	2	4	MAN	C4-C5-C6-O6
10	W	6	MAN	C4-C5-C6-O6
10	n	6	MAN	C4-C5-C6-O6
10	4	6	MAN	C4-C5-C6-O6

Continued on next page...

PRELIMINARY VALIDATION REPORT

Continued from previous page...

Mol	Chain	Res	Type	Atoms
7	S	1	NAG	C1-C2-N2-C7
7	j	1	NAG	C1-C2-N2-C7
7	0	1	NAG	C1-C2-N2-C7
6	b	2	NAG	O5-C5-C6-O6
6	s	2	NAG	O5-C5-C6-O6
6	9	2	NAG	O5-C5-C6-O6
9	V	2	NAG	C4-C5-C6-O6
9	m	2	NAG	C4-C5-C6-O6
9	3	2	NAG	C4-C5-C6-O6
6	c	1	NAG	C4-C5-C6-O6
6	t	1	NAG	C4-C5-C6-O6
6	AA	1	NAG	C4-C5-C6-O6
5	T	2	NAG	C4-C5-C6-O6
5	k	2	NAG	C4-C5-C6-O6
5	1	2	NAG	C4-C5-C6-O6
6	N	1	NAG	C4-C5-C6-O6
6	e	1	NAG	C4-C5-C6-O6
6	v	1	NAG	C4-C5-C6-O6
8	U	2	NAG	C4-C5-C6-O6
8	l	2	NAG	C4-C5-C6-O6
8	2	2	NAG	C4-C5-C6-O6
10	W	1	NAG	C4-C5-C6-O6
10	n	1	NAG	C4-C5-C6-O6
10	4	1	NAG	C4-C5-C6-O6
6	N	2	NAG	C4-C5-C6-O6
6	e	2	NAG	C4-C5-C6-O6
6	v	2	NAG	C4-C5-C6-O6
7	S	4	MAN	C4-C5-C6-O6
7	j	4	MAN	C4-C5-C6-O6
7	0	4	MAN	C4-C5-C6-O6
6	b	2	NAG	C4-C5-C6-O6
6	s	2	NAG	C4-C5-C6-O6
6	9	2	NAG	C4-C5-C6-O6
8	U	4	MAN	O5-C5-C6-O6
8	l	4	MAN	O5-C5-C6-O6
8	2	4	MAN	O5-C5-C6-O6
9	V	2	NAG	O5-C5-C6-O6
9	m	2	NAG	O5-C5-C6-O6
9	3	2	NAG	O5-C5-C6-O6
5	O	2	NAG	C4-C5-C6-O6
6	R	1	NAG	O5-C5-C6-O6
6	i	1	NAG	O5-C5-C6-O6

Continued on next page...

PRELIMINARY VALIDATION REPORT

Continued from previous page...

Mol	Chain	Res	Type	Atoms
6	z	1	NAG	O5-C5-C6-O6
5	M	1	NAG	C4-C5-C6-O6
5	d	1	NAG	C4-C5-C6-O6
5	f	2	NAG	C4-C5-C6-O6
5	u	1	NAG	C4-C5-C6-O6
5	w	2	NAG	C4-C5-C6-O6
5	P	1	NAG	O5-C5-C6-O6
5	g	1	NAG	O5-C5-C6-O6
5	x	1	NAG	O5-C5-C6-O6
10	W	2	NAG	O5-C5-C6-O6
10	n	2	NAG	O5-C5-C6-O6
10	4	2	NAG	O5-C5-C6-O6
7	S	2	NAG	C4-C5-C6-O6
7	j	2	NAG	C4-C5-C6-O6
7	0	2	NAG	C4-C5-C6-O6
7	S	3	BMA	C4-C5-C6-O6
7	j	3	BMA	C4-C5-C6-O6
7	0	3	BMA	C4-C5-C6-O6
10	W	2	NAG	C4-C5-C6-O6
10	n	2	NAG	C4-C5-C6-O6
10	4	2	NAG	C4-C5-C6-O6
5	P	3	BMA	O5-C5-C6-O6
5	g	3	BMA	O5-C5-C6-O6
5	x	3	BMA	O5-C5-C6-O6
8	U	2	NAG	O5-C5-C6-O6
8	l	2	NAG	O5-C5-C6-O6
8	2	2	NAG	O5-C5-C6-O6
6	b	1	NAG	C1-C2-N2-C7
6	s	1	NAG	C1-C2-N2-C7
6	9	1	NAG	C1-C2-N2-C7
8	U	2	NAG	C1-C2-N2-C7
8	l	2	NAG	C1-C2-N2-C7
8	2	2	NAG	C1-C2-N2-C7
10	W	7	MAN	O5-C5-C6-O6
10	n	7	MAN	O5-C5-C6-O6
10	4	7	MAN	O5-C5-C6-O6
6	R	1	NAG	C4-C5-C6-O6
6	i	1	NAG	C4-C5-C6-O6
6	z	1	NAG	C4-C5-C6-O6
7	S	5	MAN	O5-C5-C6-O6
7	j	5	MAN	O5-C5-C6-O6
7	0	5	MAN	O5-C5-C6-O6

Continued on next page...

PRELIMINARY VALIDATION REPORT

Continued from previous page...

Mol	Chain	Res	Type	Atoms
5	P	3	BMA	C4-C5-C6-O6
5	g	3	BMA	C4-C5-C6-O6
5	x	3	BMA	C4-C5-C6-O6
7	S	2	NAG	O5-C5-C6-O6
7	j	2	NAG	O5-C5-C6-O6
7	0	2	NAG	O5-C5-C6-O6
7	S	3	BMA	O5-C5-C6-O6
7	j	3	BMA	O5-C5-C6-O6
7	0	3	BMA	O5-C5-C6-O6
9	m	6	MAN	O5-C5-C6-O6
9	3	6	MAN	O5-C5-C6-O6
9	V	6	MAN	O5-C5-C6-O6
9	V	7	MAN	O5-C5-C6-O6
9	m	7	MAN	O5-C5-C6-O6
9	3	7	MAN	O5-C5-C6-O6
6	b	1	NAG	C4-C5-C6-O6
6	s	1	NAG	C4-C5-C6-O6
9	m	3	BMA	O5-C5-C6-O6
10	W	8	MAN	O5-C5-C6-O6
10	n	8	MAN	O5-C5-C6-O6
10	4	8	MAN	O5-C5-C6-O6
6	9	1	NAG	C4-C5-C6-O6
9	3	3	BMA	O5-C5-C6-O6
6	a	2	NAG	C4-C5-C6-O6
6	8	2	NAG	C4-C5-C6-O6
6	r	2	NAG	C4-C5-C6-O6
8	l	1	NAG	C4-C5-C6-O6
8	2	1	NAG	C4-C5-C6-O6
6	Q	1	NAG	O5-C5-C6-O6
6	h	1	NAG	O5-C5-C6-O6
6	y	1	NAG	O5-C5-C6-O6
9	V	3	BMA	O5-C5-C6-O6
8	U	1	NAG	C4-C5-C6-O6
9	V	2	NAG	C3-C2-N2-C7
9	m	2	NAG	C3-C2-N2-C7
9	3	2	NAG	C3-C2-N2-C7
8	2	1	NAG	O5-C5-C6-O6
8	U	1	NAG	O5-C5-C6-O6
8	l	1	NAG	O5-C5-C6-O6
5	P	1	NAG	C4-C5-C6-O6
5	g	1	NAG	C4-C5-C6-O6
5	x	1	NAG	C4-C5-C6-O6

Continued on next page...

PRELIMINARY VALIDATION REPORT

Continued from previous page...

Mol	Chain	Res	Type	Atoms
5	Y	2	NAG	O5-C5-C6-O6
5	p	2	NAG	O5-C5-C6-O6
5	6	2	NAG	O5-C5-C6-O6
5	w	3	BMA	O5-C5-C6-O6
5	f	3	BMA	O5-C5-C6-O6
7	j	1	NAG	O5-C5-C6-O6
7	S	1	NAG	O5-C5-C6-O6
7	0	1	NAG	O5-C5-C6-O6
7	0	7	MAN	C4-C5-C6-O6
5	O	3	BMA	O5-C5-C6-O6
9	V	4	MAN	C4-C5-C6-O6
9	m	4	MAN	C4-C5-C6-O6
7	S	7	MAN	C4-C5-C6-O6
7	j	7	MAN	C4-C5-C6-O6
9	3	4	MAN	C4-C5-C6-O6
7	j	7	MAN	O5-C5-C6-O6
7	S	7	MAN	O5-C5-C6-O6
7	0	7	MAN	O5-C5-C6-O6
5	f	3	BMA	C4-C5-C6-O6
5	w	3	BMA	C4-C5-C6-O6
7	S	2	NAG	C1-C2-N2-C7
7	j	2	NAG	C1-C2-N2-C7
7	0	2	NAG	C1-C2-N2-C7
5	O	3	BMA	C4-C5-C6-O6
5	d	3	BMA	O5-C5-C6-O6
5	u	3	BMA	O5-C5-C6-O6
5	M	3	BMA	O5-C5-C6-O6
9	m	3	BMA	C4-C5-C6-O6
9	3	3	BMA	C4-C5-C6-O6
9	V	3	BMA	C4-C5-C6-O6
5	1	1	NAG	C1-C2-N2-C7
6	c	1	NAG	C1-C2-N2-C7
6	t	1	NAG	C1-C2-N2-C7
6	AA	1	NAG	C1-C2-N2-C7
7	S	1	NAG	C3-C2-N2-C7
7	j	1	NAG	C3-C2-N2-C7
7	0	1	NAG	C3-C2-N2-C7
9	3	1	NAG	C3-C2-N2-C7
10	W	1	NAG	C3-C2-N2-C7
10	n	1	NAG	C3-C2-N2-C7
10	4	1	NAG	C3-C2-N2-C7
5	k	1	NAG	C1-C2-N2-C7

Continued on next page...

PRELIMINARY VALIDATION REPORT

Continued from previous page...

Mol	Chain	Res	Type	Atoms
5	T	1	NAG	C1-C2-N2-C7
5	T	3	BMA	O5-C5-C6-O6
5	k	3	BMA	O5-C5-C6-O6
5	1	3	BMA	O5-C5-C6-O6
6	a	2	NAG	C1-C2-N2-C7
6	r	2	NAG	C1-C2-N2-C7
6	8	2	NAG	C1-C2-N2-C7
5	u	3	BMA	C4-C5-C6-O6
5	M	3	BMA	C4-C5-C6-O6
5	d	3	BMA	C4-C5-C6-O6
5	T	3	BMA	C4-C5-C6-O6
5	k	3	BMA	C4-C5-C6-O6
9	m	1	NAG	C1-C2-N2-C7
9	3	1	NAG	C1-C2-N2-C7
5	1	3	BMA	C4-C5-C6-O6
6	b	1	NAG	C3-C2-N2-C7
6	s	1	NAG	C3-C2-N2-C7
6	9	1	NAG	C3-C2-N2-C7
8	U	2	NAG	C3-C2-N2-C7
8	l	2	NAG	C3-C2-N2-C7
8	2	2	NAG	C3-C2-N2-C7
9	V	1	NAG	C3-C2-N2-C7
9	m	1	NAG	C3-C2-N2-C7
9	V	1	NAG	C1-C2-N2-C7
8	U	5	MAN	C4-C5-C6-O6
8	l	5	MAN	C4-C5-C6-O6
8	2	5	MAN	C4-C5-C6-O6

There are no ring outliers.

No monomer is involved in short contacts.

The following is a two-dimensional graphical depiction of Mogul quality analysis of bond lengths, bond angles, torsion angles, and ring geometry for oligosaccharide.

PRELIMINARY VALIDATION REPORT

Oligosaccharide Chain M

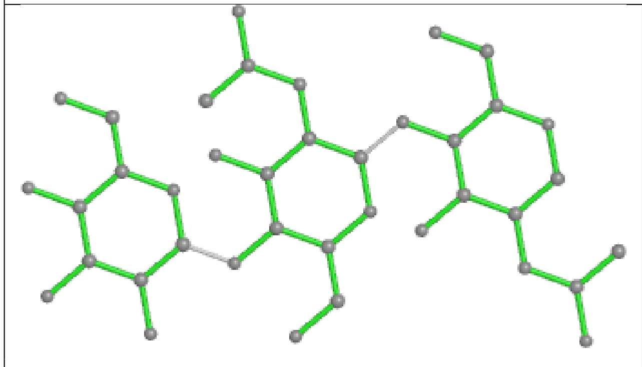

Bond lengths

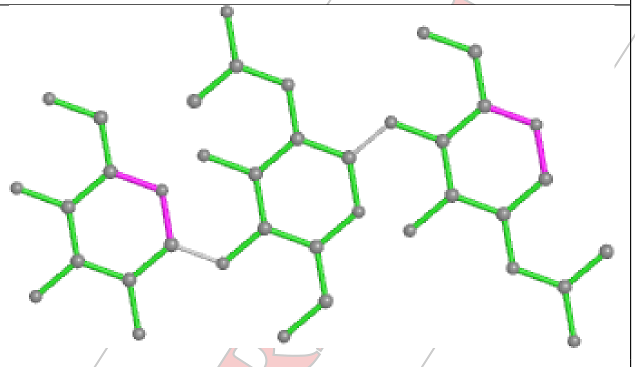

Bond angles

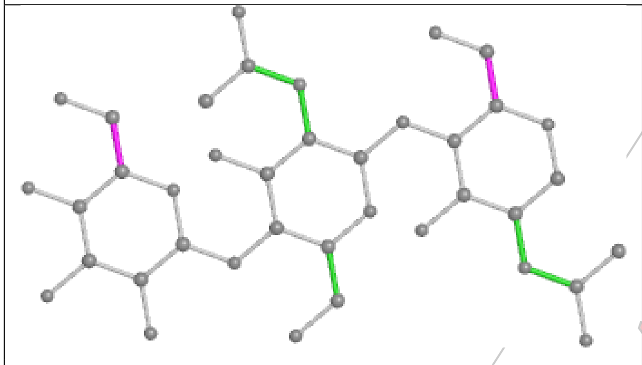

Torsions

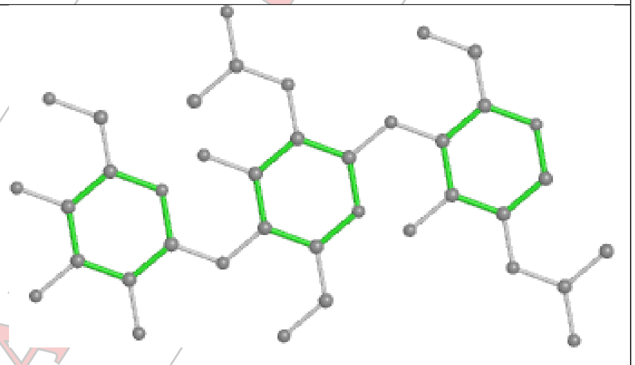

Rings

Oligosaccharide Chain O

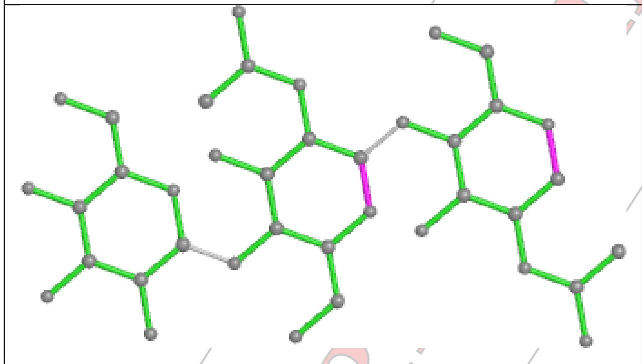

Bond lengths

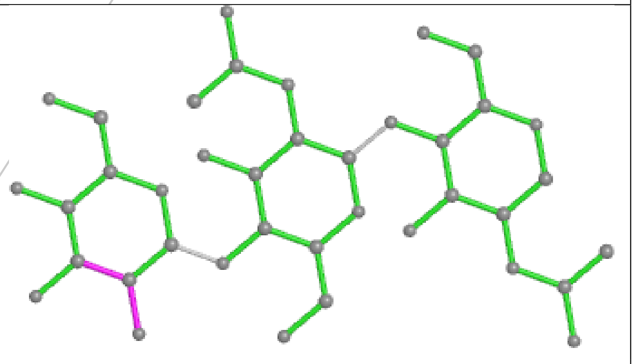

Bond angles

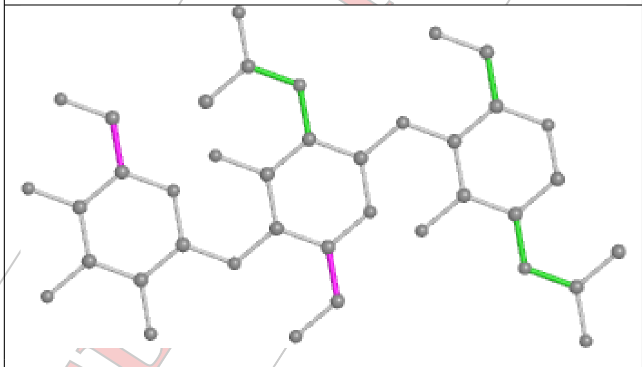

Torsions

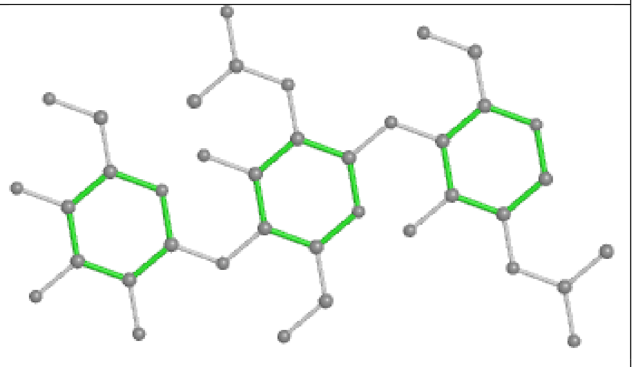

Rings

Oligosaccharide Chain P

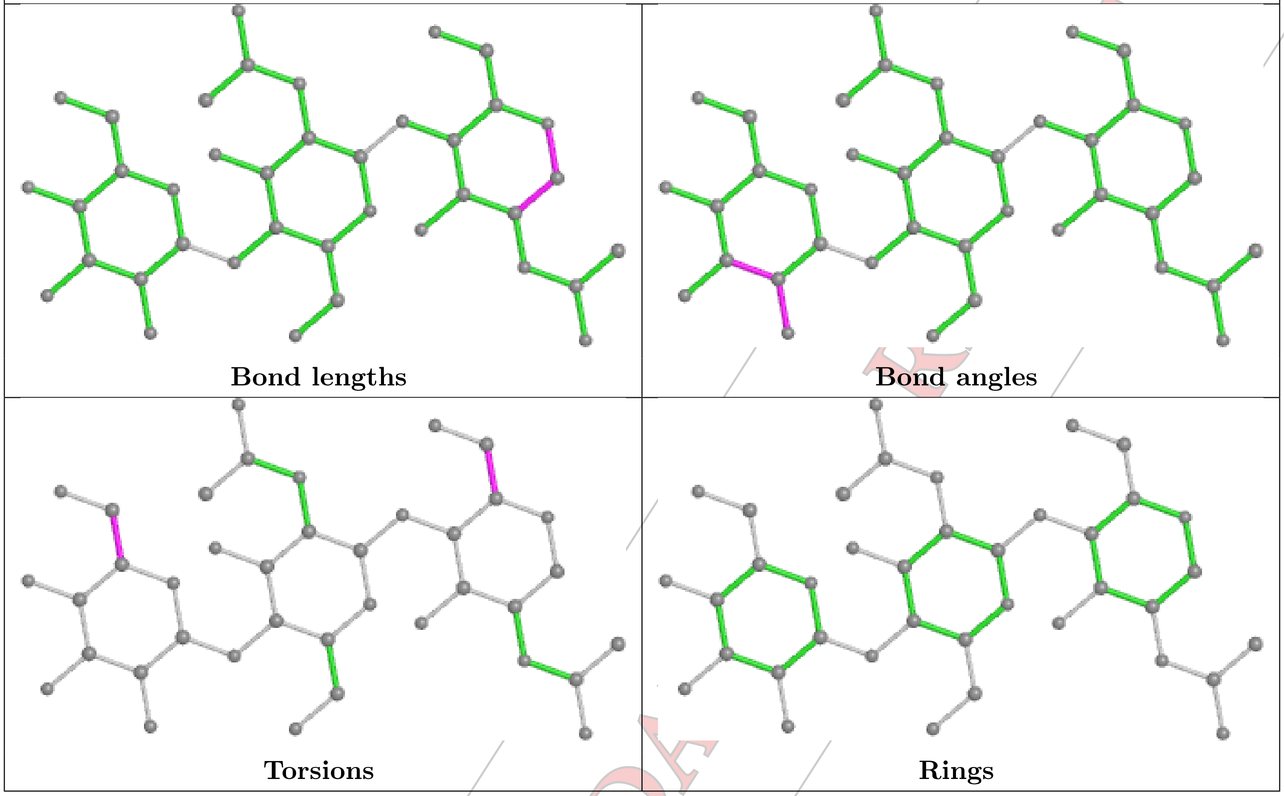

Oligosaccharide Chain T

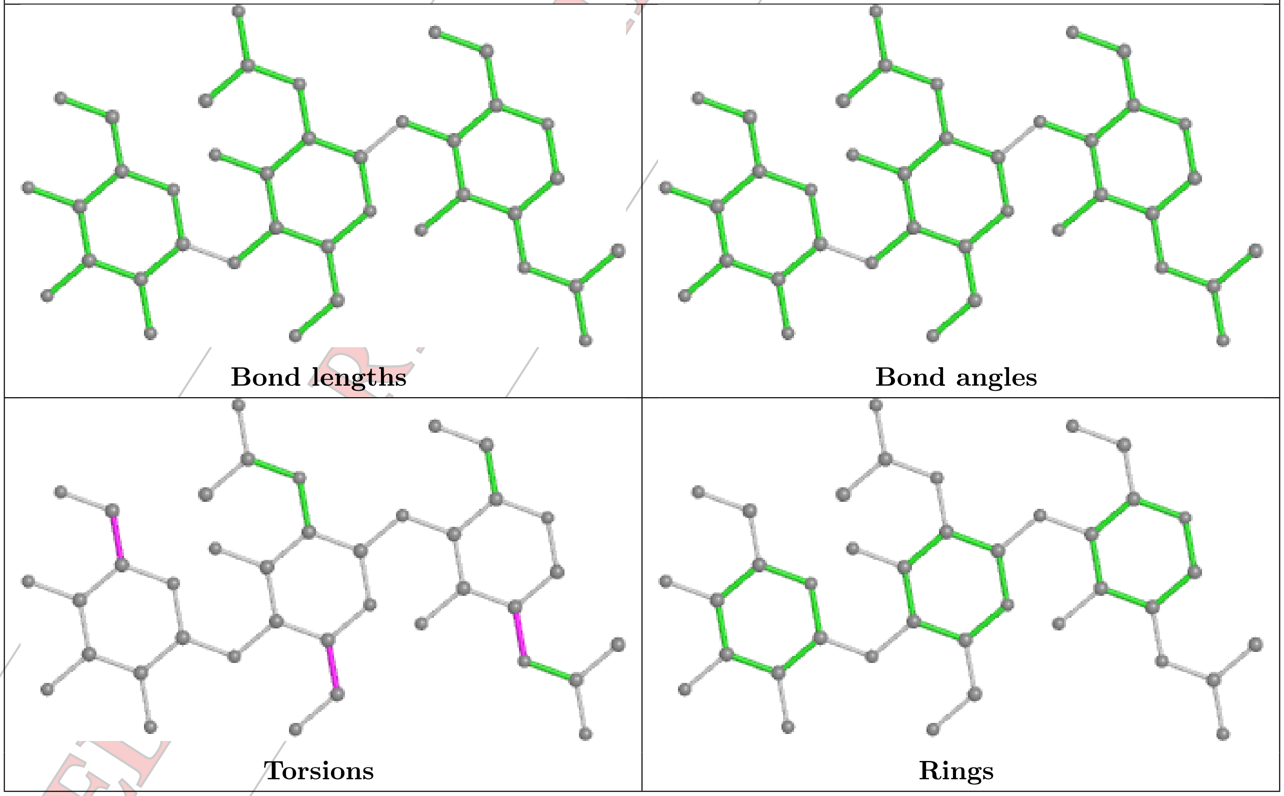

Oligosaccharide Chain Y

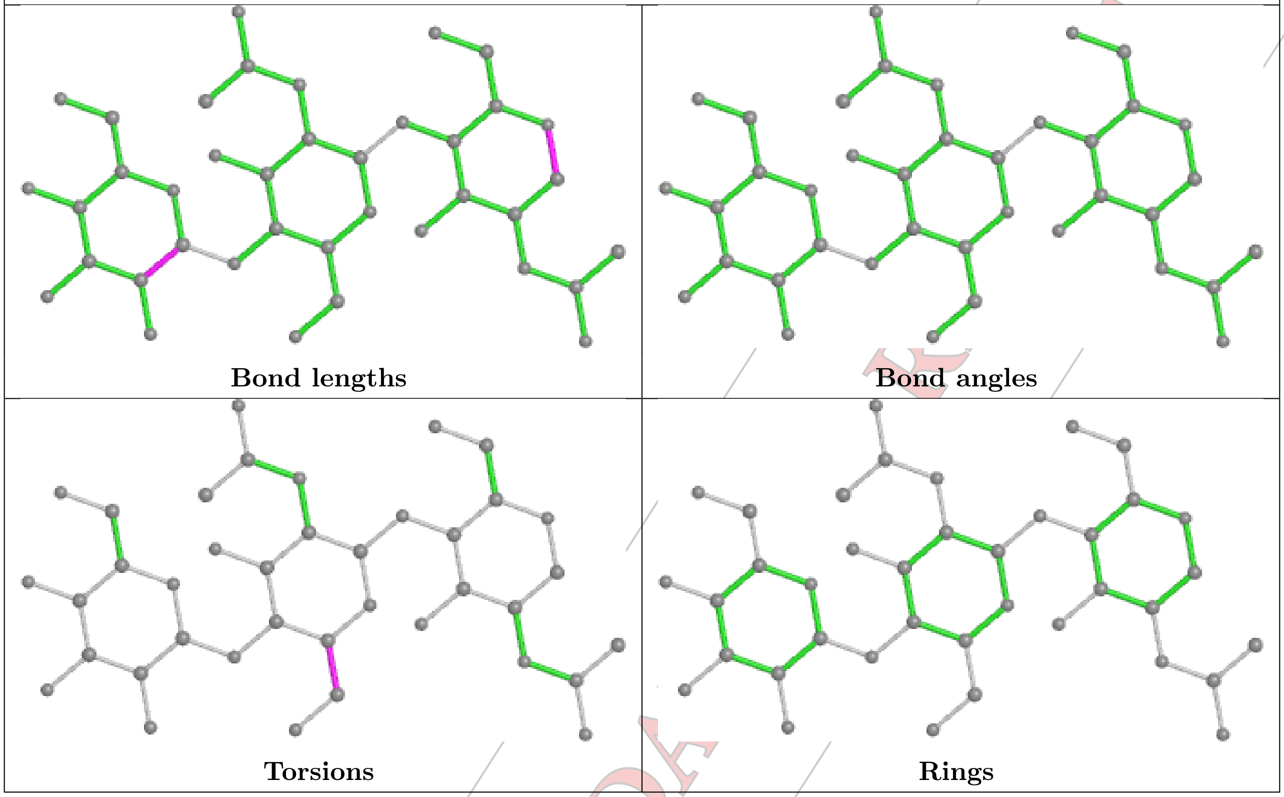

Oligosaccharide Chain Z

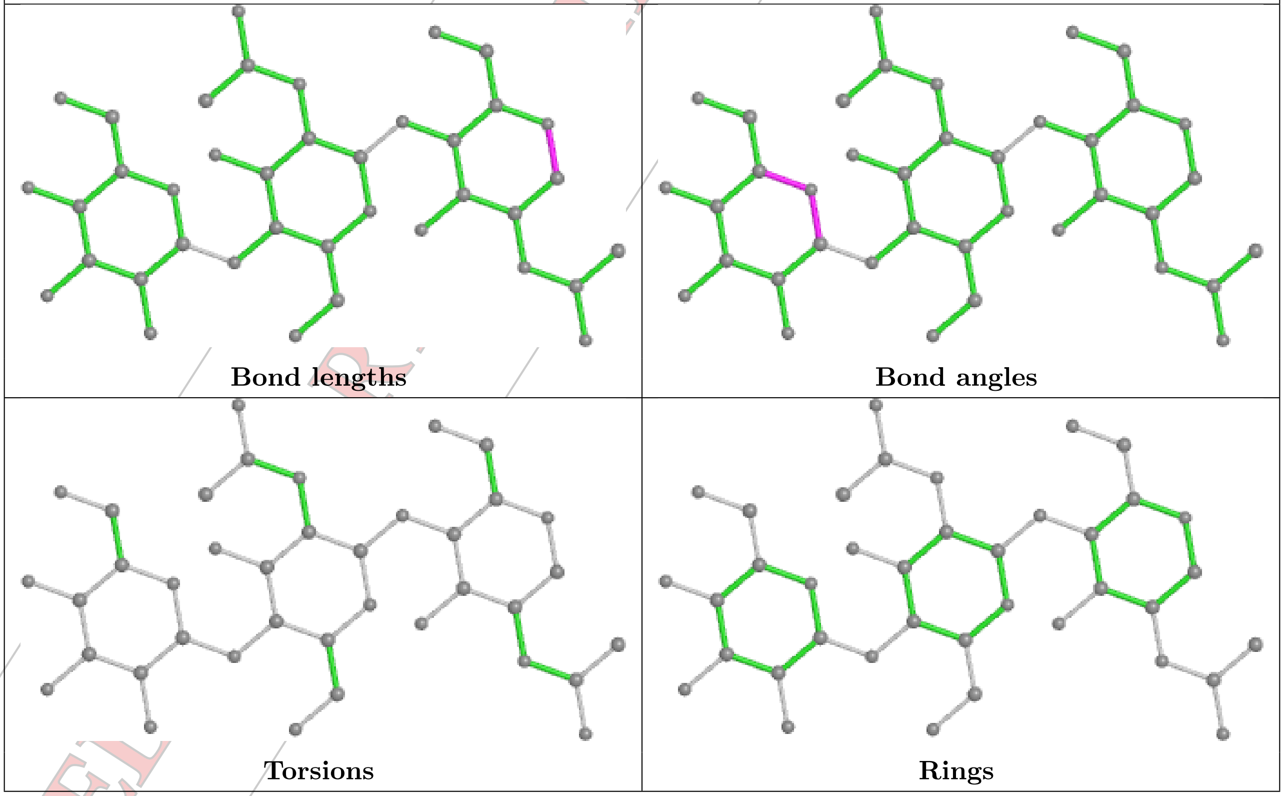

Oligosaccharide Chain N

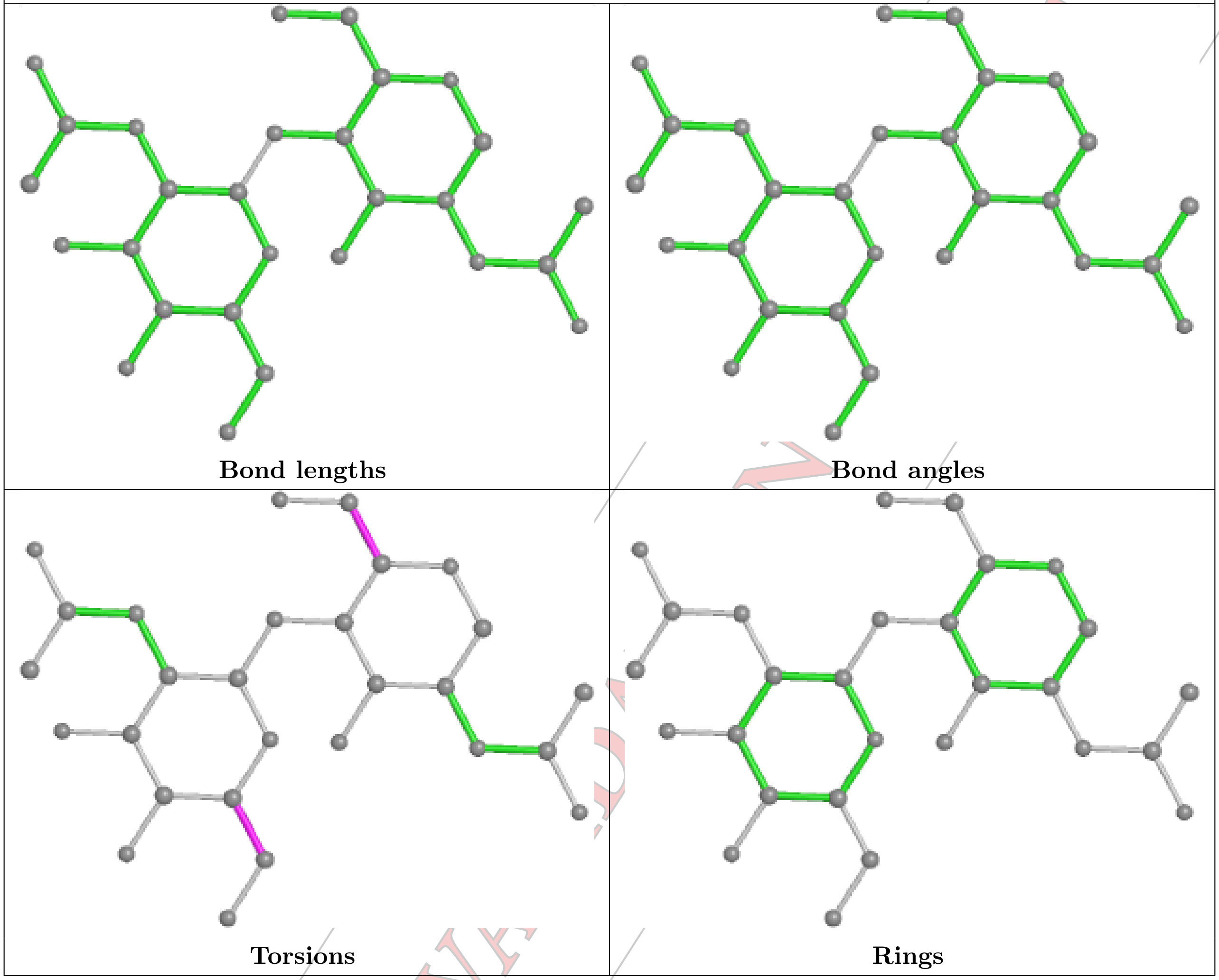

Oligosaccharide Chain Q

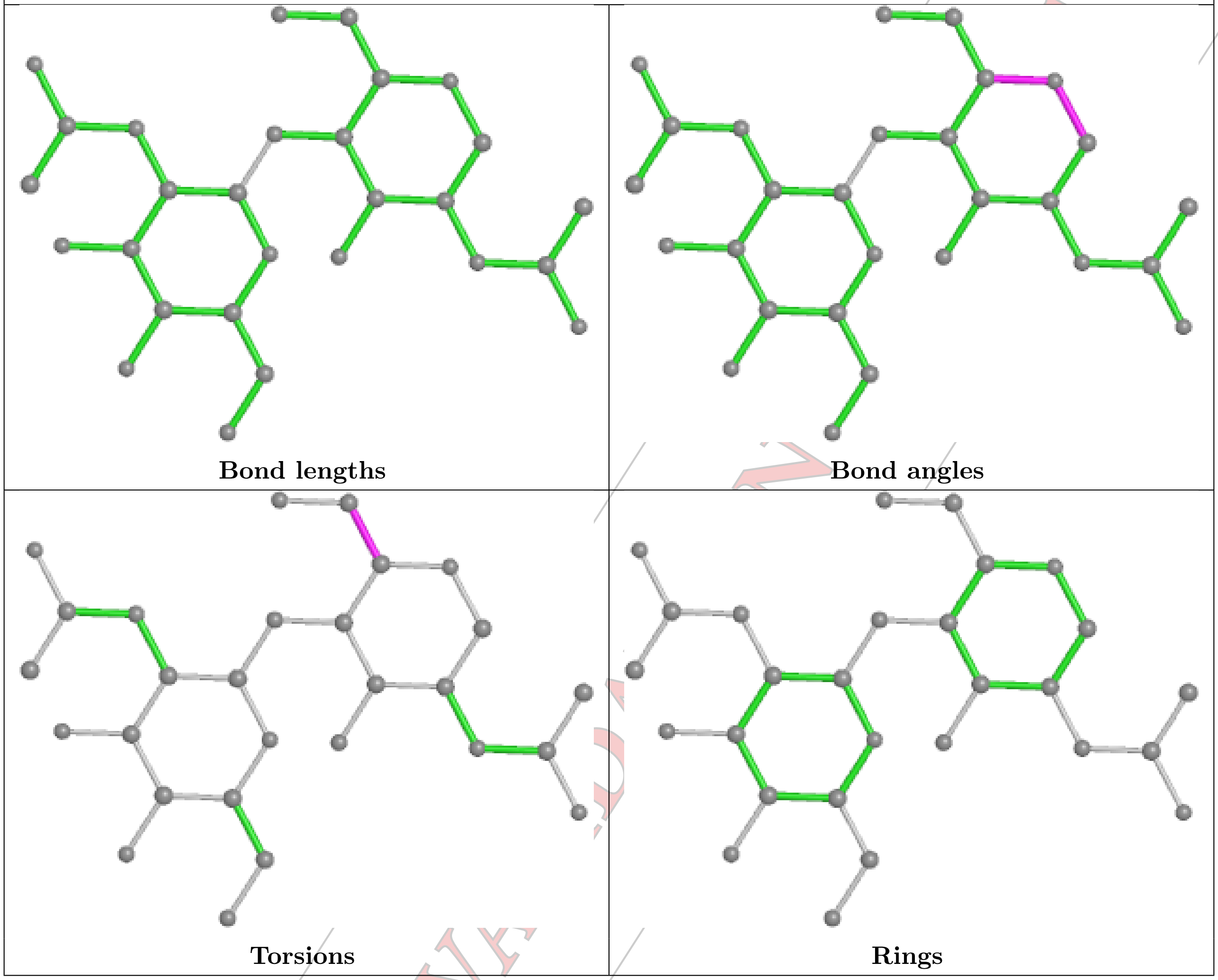

PRELIMINARY VALIDATION

Oligosaccharide Chain R

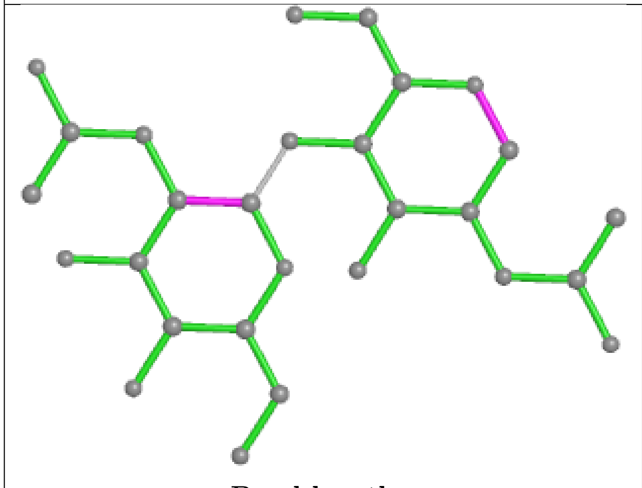

Bond lengths

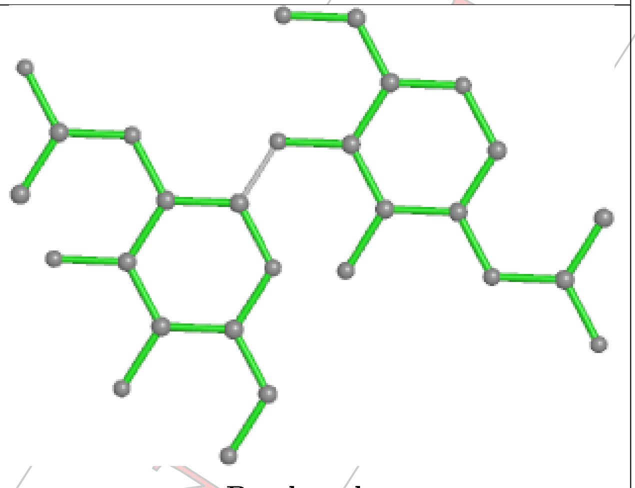

Bond angles

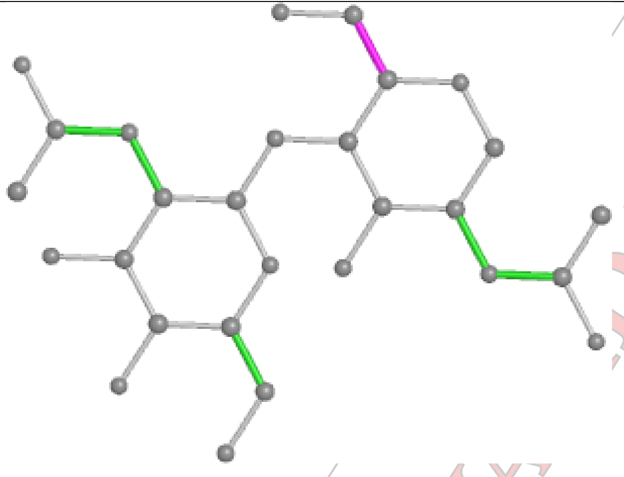

Torsions

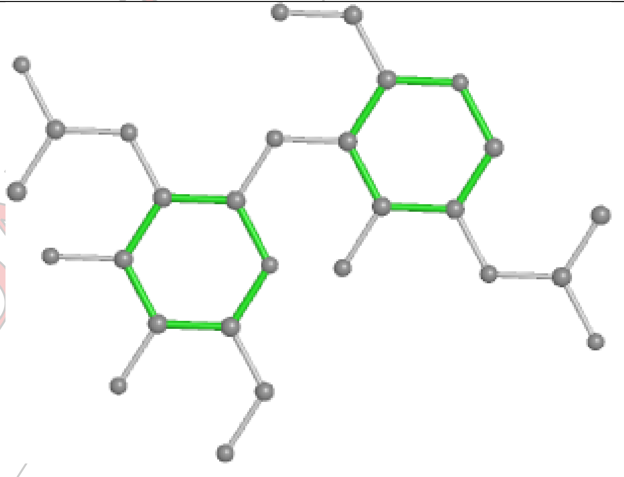

Rings

PRELIMINARY

Oligosaccharide Chain X

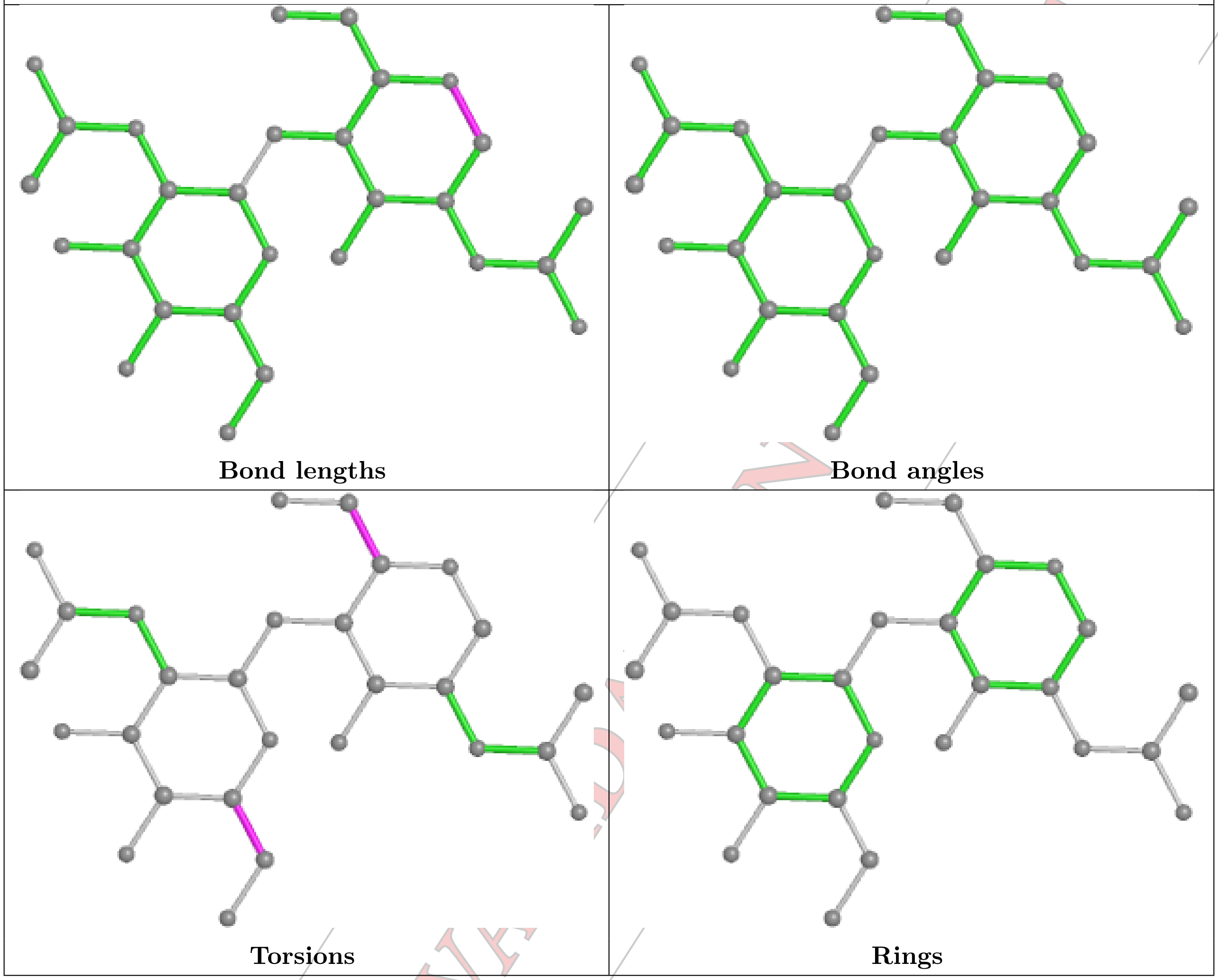

PRELIMINARY

Oligosaccharide Chain AA

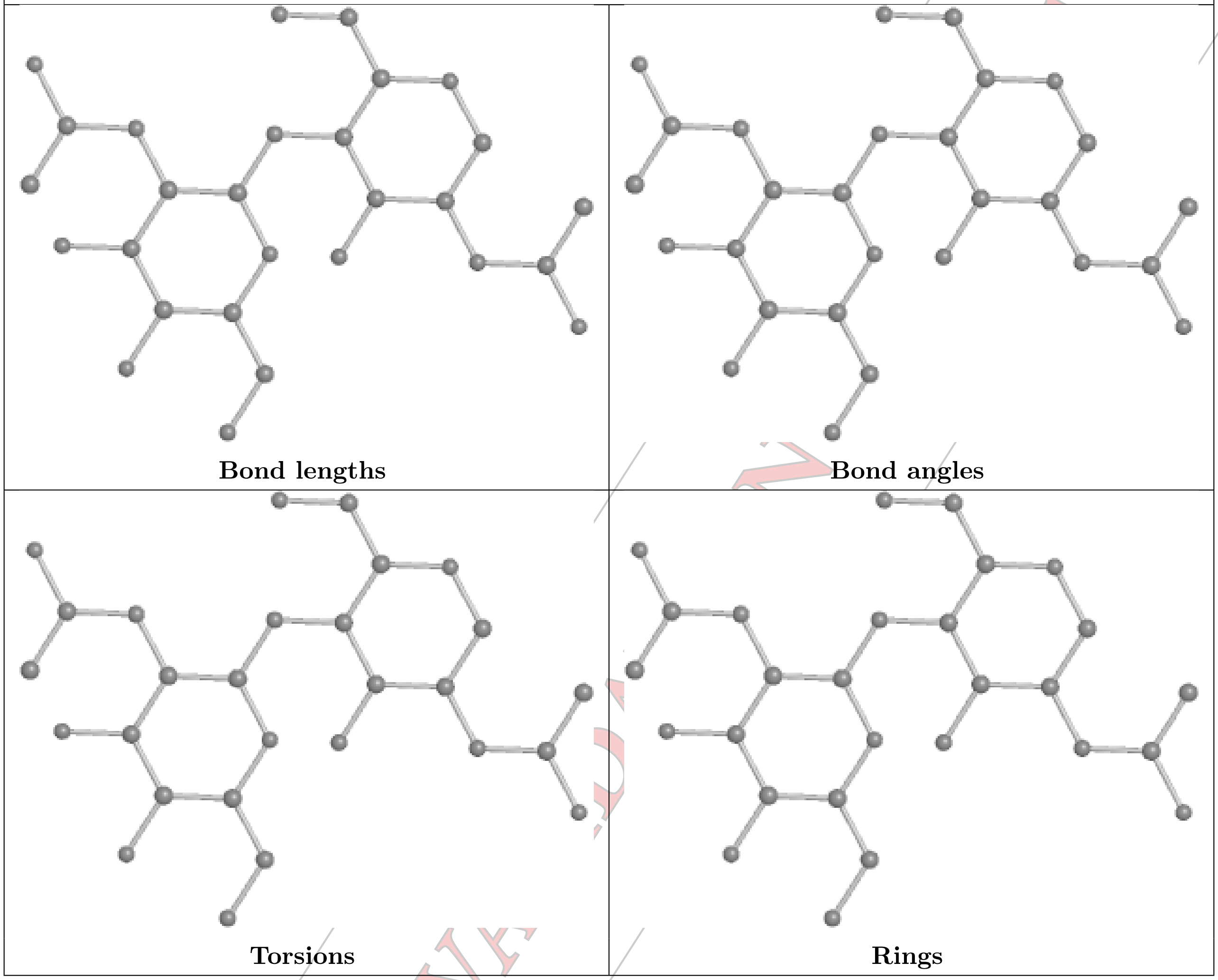

Oligosaccharide Chain S

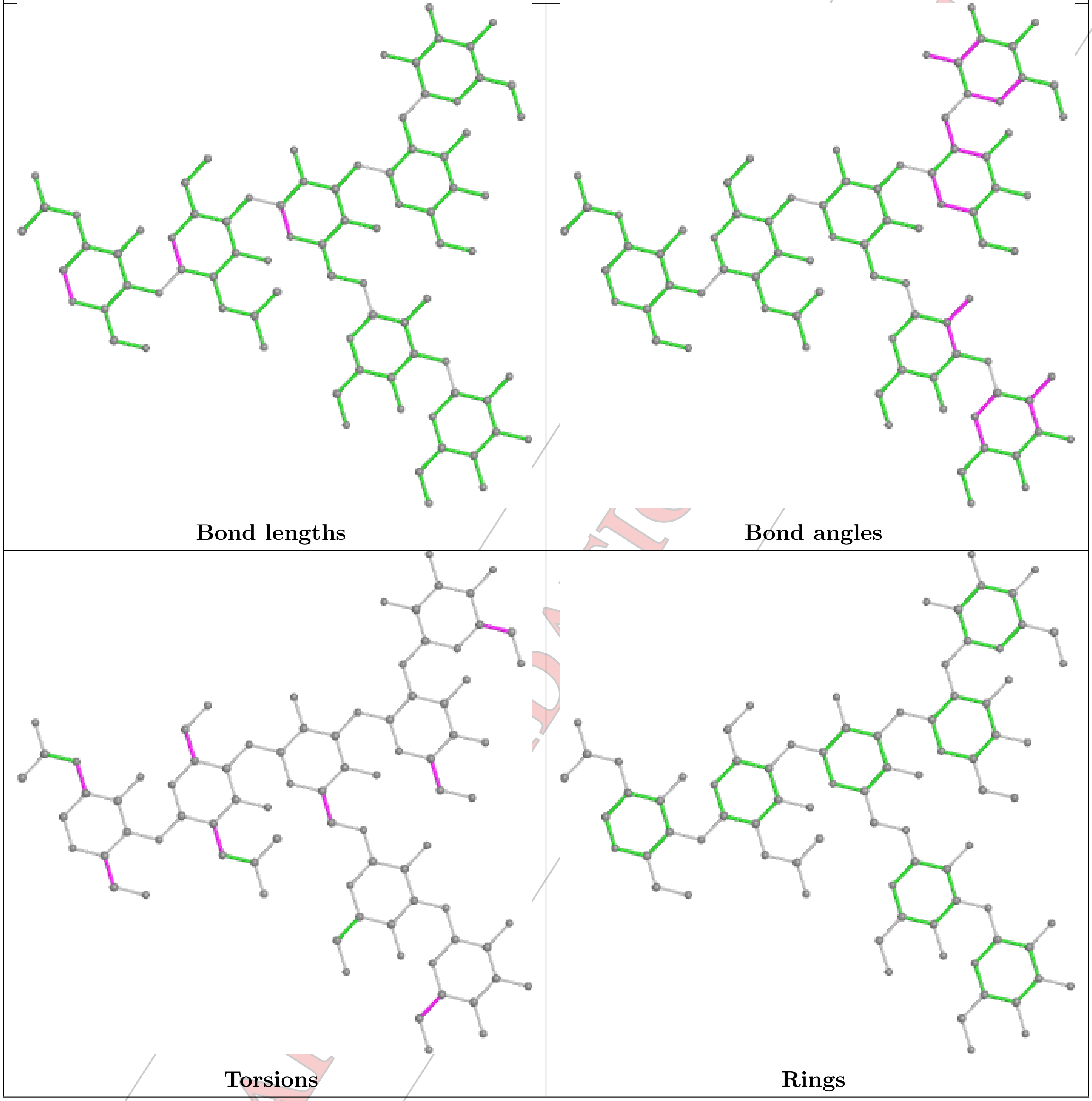

Oligosaccharide Chain U

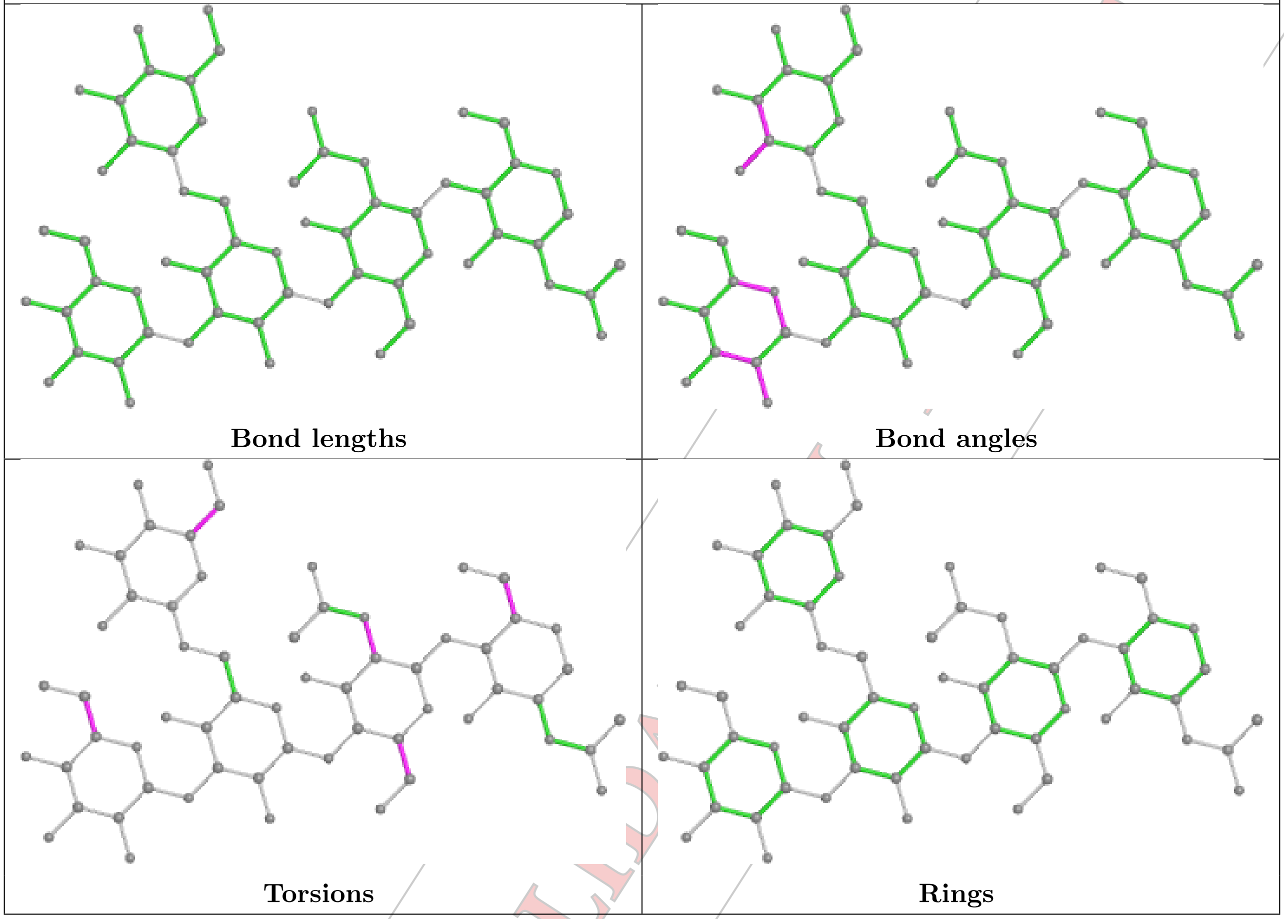

Oligosaccharide Chain V

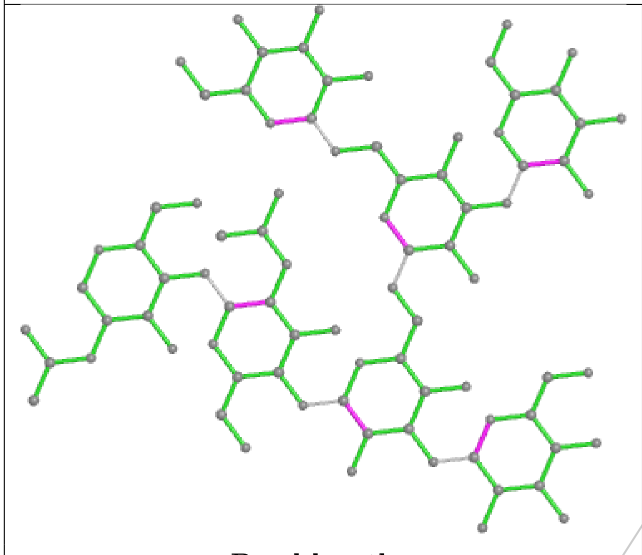

Bond lengths

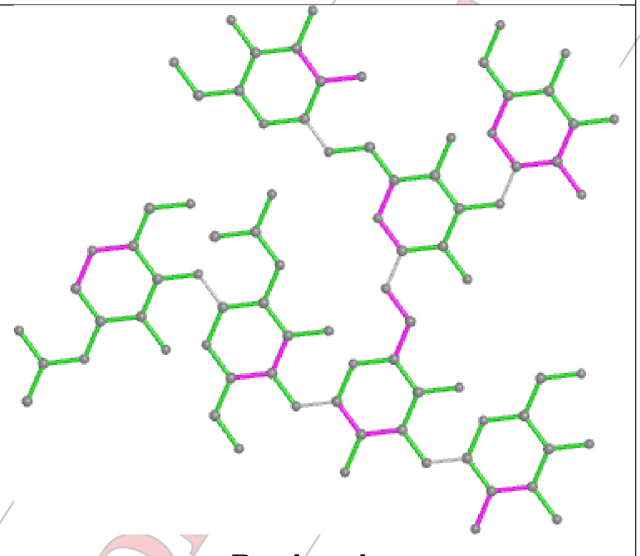

Bond angles

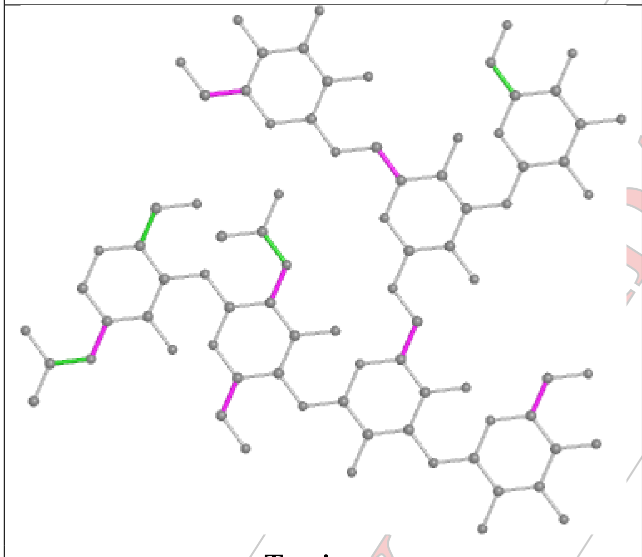

Torsions

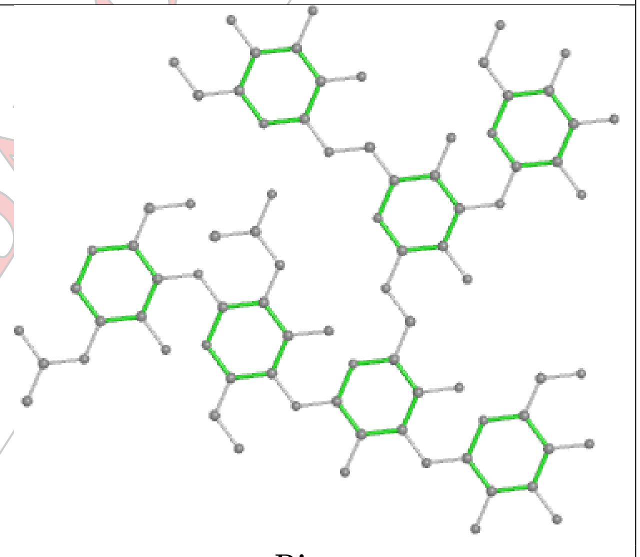

Rings

PRELIMINARY

Oligosaccharide Chain W

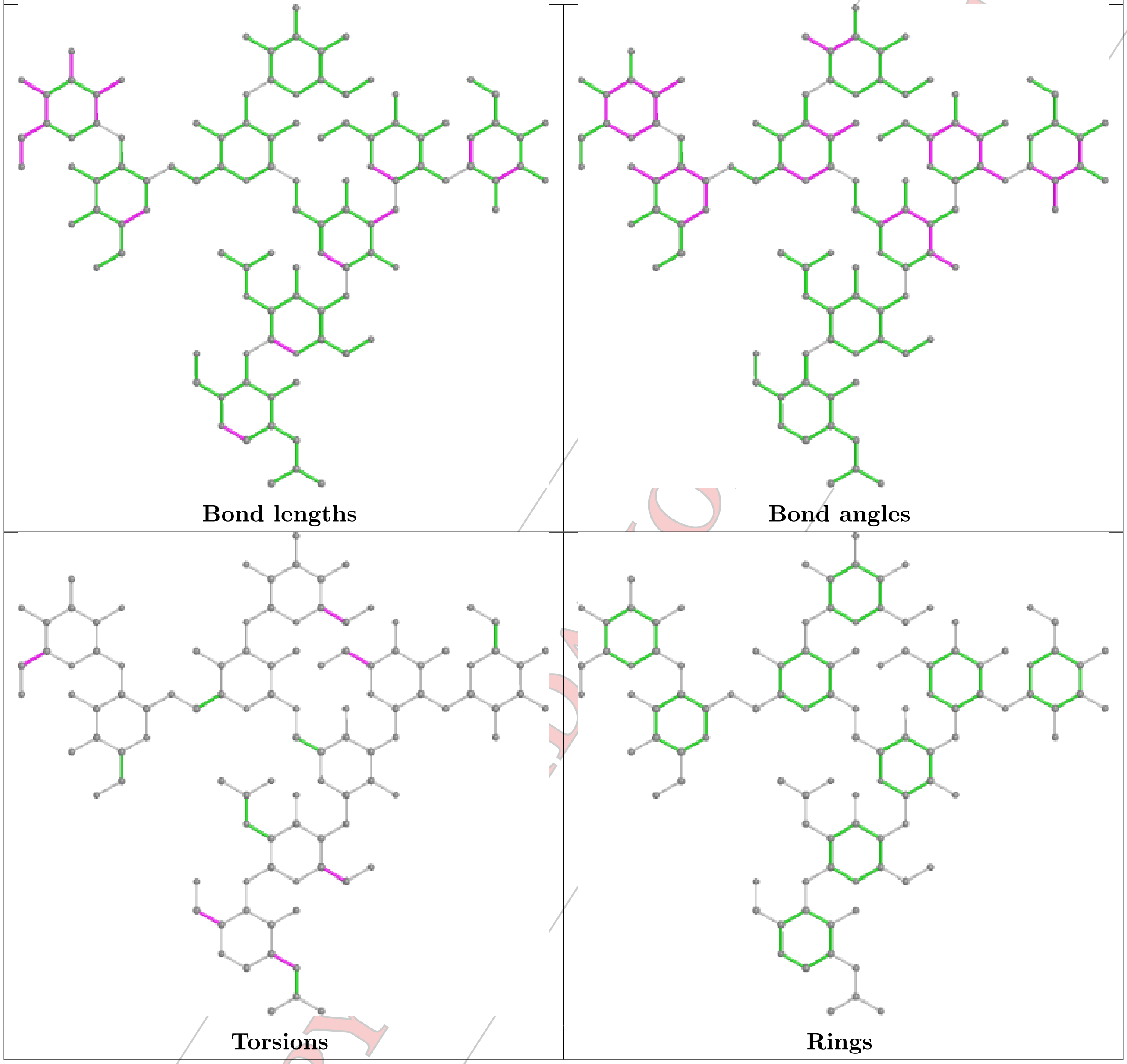

5.6 Ligand geometry (i)

12 ligands are modelled in this entry.

In the following table, the Counts columns list the number of bonds (or angles) for which Mogul statistics could be retrieved, the number of bonds (or angles) that are observed in the model and the number of bonds (or angles) that are defined in the Chemical Component Dictionary. The Link column lists molecule types, if any, to which the group is linked. The Z score for a bond length (or angle) is the number of standard deviations the observed value is removed from the expected value. A bond length (or angle) with $|Z| > 2$ is considered an outlier worth inspection. RMSZ is the root-mean-square of all Z scores of the bond lengths (or angles).

PRELIMINARY

Mol	Type	Chain	Res	Link	Bond lengths			Bond angles		
					Counts	RMSZ	#|Z| > 2	Counts	RMSZ	#|Z| > 2
12	NAG	F	1637	2	14,14,15	0.14	0	17,19,21	0.51	0
12	NAG	B	1618	2	14,14,15	0.28	0	17,19,21	0.40	0
12	NAG	E	1343	1	14,14,15	0.30	0	17,19,21	0.38	0
12	NAG	J	1618	2	14,14,15	0.27	0	17,19,21	0.40	0
11	MAN	E	1340	-	11,11,12	1.22	1 (9%)	15,15,17	1.19	1 (6%)
12	NAG	J	1637	2	14,14,15	0.14	0	17,19,21	0.51	0
12	NAG	B	1637	2	14,14,15	0.14	0	17,19,21	0.51	0
11	MAN	A	1340	-	11,11,12	1.21	1 (9%)	15,15,17	1.19	1 (6%)
11	MAN	I	1340	-	11,11,12	1.22	1 (9%)	15,15,17	1.19	1 (6%)
12	NAG	F	1618	2	14,14,15	0.26	0	17,19,21	0.41	0
12	NAG	A	1343	1	14,14,15	0.31	0	17,19,21	0.38	0
12	NAG	I	1343	1	14,14,15	0.30	0	17,19,21	0.38	0

In the following table, the Chirals column lists the number of chiral outliers, the number of chiral centers analysed, the number of these observed in the model and the number defined in the Chemical Component Dictionary. Similar counts are reported in the Torsion and Rings columns. '-' means no outliers of that kind were identified.

Mol	Type	Chain	Res	Link	Chirals	Torsions	Rings
12	NAG	F	1637	2	-	2/6/23/26	0/1/1/1
12	NAG	B	1618	2	-	1/6/23/26	0/1/1/1
12	NAG	E	1343	1	-	2/6/23/26	0/1/1/1
12	NAG	J	1618	2	-	1/6/23/26	0/1/1/1
11	MAN	E	1340	-	-	1/2/19/22	0/1/1/1
12	NAG	J	1637	2	-	2/6/23/26	0/1/1/1
12	NAG	B	1637	2	-	2/6/23/26	0/1/1/1
11	MAN	A	1340	-	-	1/2/19/22	0/1/1/1
11	MAN	I	1340	-	-	1/2/19/22	0/1/1/1
12	NAG	F	1618	2	-	1/6/23/26	0/1/1/1
12	NAG	A	1343	1	-	2/6/23/26	0/1/1/1
12	NAG	I	1343	1	-	2/6/23/26	0/1/1/1

All (3) bond length outliers are listed below:

Mol	Chain	Res	Type	Atoms	Z	Observed(Å)	Ideal(Å)
11	A	1340	MAN	C4-C5	2.60	1.58	1.53
11	I	1340	MAN	C4-C5	2.59	1.58	1.53
11	E	1340	MAN	C4-C5	2.58	1.58	1.53

All (3) bond angle outliers are listed below:

PRELIMINARY VALIDATION REPORT

Mol	Chain	Res	Type	Atoms	Z	Observed(^{o})	Ideal(^{o})
11	E	1340	MAN	O2-C2-C3	-2.80	104.53	110.14
11	I	1340	MAN	O2-C2-C3	-2.79	104.55	110.14
11	A	1340	MAN	O2-C2-C3	-2.79	104.56	110.14

There are no chirality outliers.

All (18) torsion outliers are listed below:

Mol	Chain	Res	Type	Atoms
12	B	1637	NAG	C4-C5-C6-O6
12	F	1637	NAG	C4-C5-C6-O6
12	J	1637	NAG	C4-C5-C6-O6
12	B	1637	NAG	O5-C5-C6-O6
12	F	1637	NAG	O5-C5-C6-O6
12	J	1637	NAG	O5-C5-C6-O6
12	B	1618	NAG	O5-C5-C6-O6
12	F	1618	NAG	O5-C5-C6-O6
12	J	1618	NAG	O5-C5-C6-O6
12	A	1343	NAG	O5-C5-C6-O6
12	E	1343	NAG	O5-C5-C6-O6
12	I	1343	NAG	O5-C5-C6-O6
11	A	1340	MAN	O5-C5-C6-O6
11	I	1340	MAN	O5-C5-C6-O6
11	E	1340	MAN	O5-C5-C6-O6
12	A	1343	NAG	C4-C5-C6-O6
12	E	1343	NAG	C4-C5-C6-O6
12	I	1343	NAG	C4-C5-C6-O6

There are no ring outliers.

No monomer is involved in short contacts.

5.7 Other polymers (i)

There are no such residues in this entry.

5.8 Polymer linkage issues (i)

The following chains have linkage breaks:

Mol	Chain	Number of breaks
1	A	1

Continued on next page...

PRELIMINARY VALIDATION REPORT

Continued from previous page...

Mol	Chain	Number of breaks
1	E	1
1	I	1

All chain breaks are listed below:

Model	Chain	Residue-1	Atom-1	Residue-2	Atom-2	Distance (Å)
1	A	218:CYS	C	219:ALA	N	1.19
1	E	218:CYS	C	219:ALA	N	1.19
1	I	218:CYS	C	219:ALA	N	1.19

PRELIMINARY VALIDATION REPORT

6 Map visualisation (i)

This section contains visualisations of the EMDB entry D_1000255470. These allow visual inspection of the internal detail of the map and identification of artifacts.

No raw map or half-maps were deposited for this entry and therefore no images, graphs, etc. pertaining to the raw map can be shown.

6.1 Orthogonal projections (i)

6.1.1 Primary map

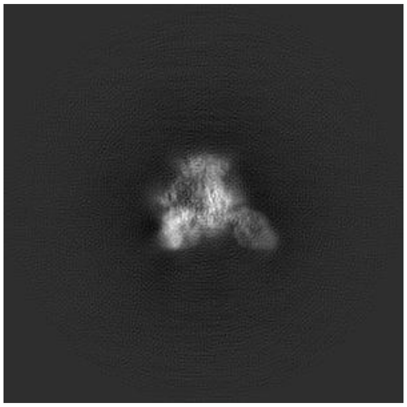

X

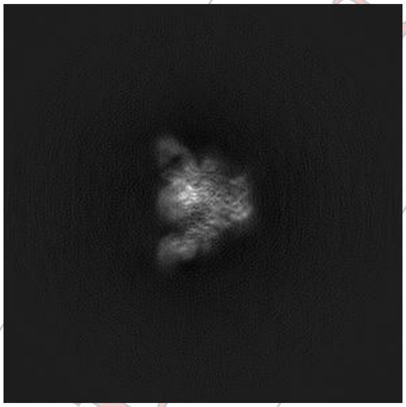

Y

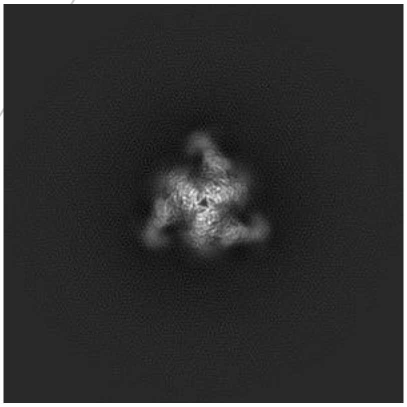

Z

The images above show the map projected in three orthogonal directions.

6.2 Central slices (i)

6.2.1 Primary map

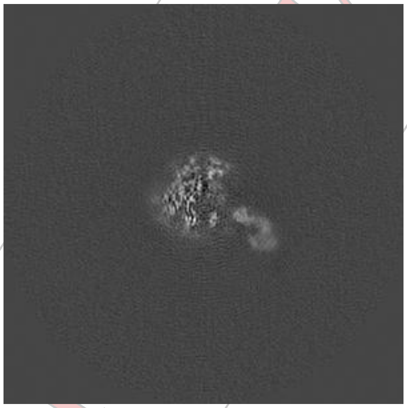

X Index: 200

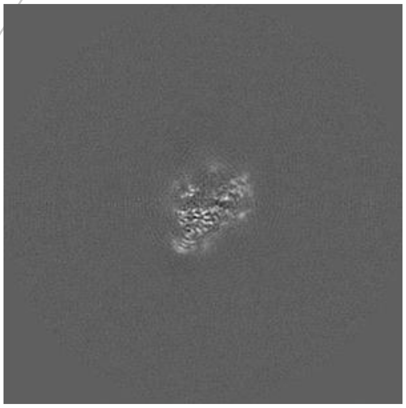

Y Index: 200

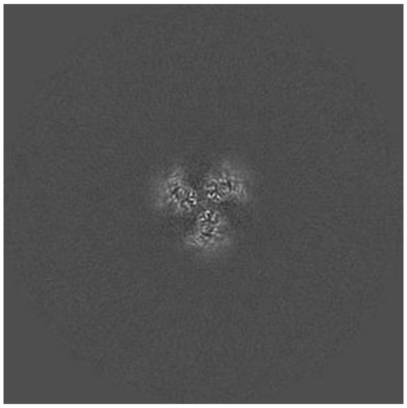

Z Index: 200

PRELIMINARY VALIDATION REPORT

The images above show central slices of the map in three orthogonal directions.

6.3 Largest variance slices (i)

6.3.1 Primary map

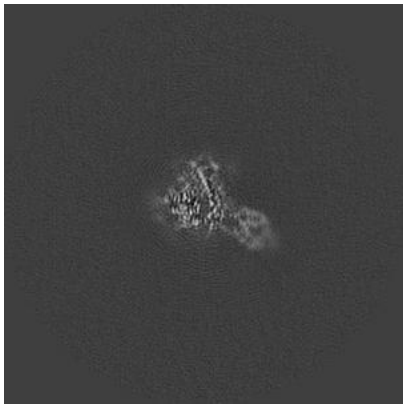

X Index: 205

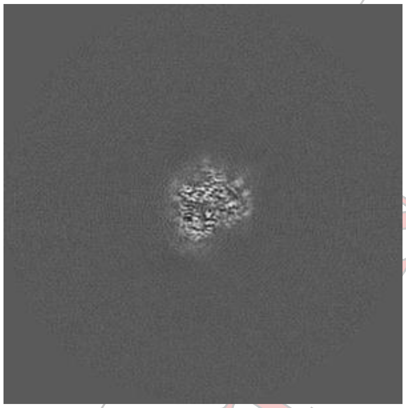

Y Index: 206

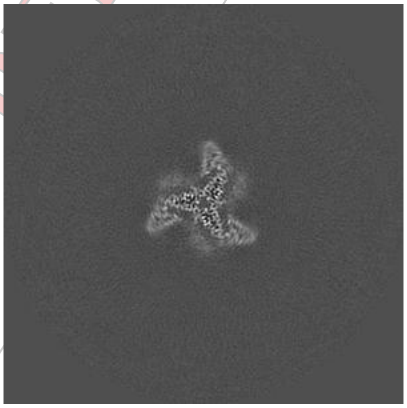

Z Index: 186

The images above show the largest variance slices of the map in three orthogonal directions.

6.4 Orthogonal surface views (i)

6.4.1 Primary map

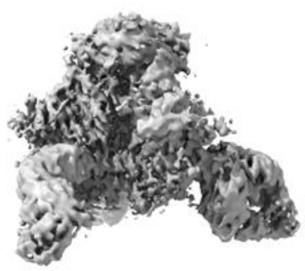

X

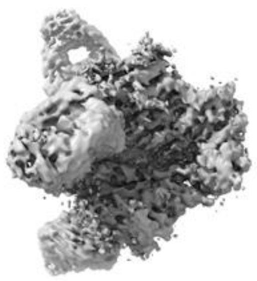

Y

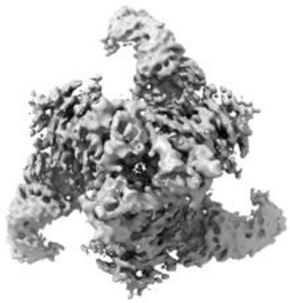

Z

The images above show the 3D surface view of the map at the recommended contour level 7.8. These images, in conjunction with the slice images, may facilitate assessment of whether an appropriate contour level has been provided.

6.5 Mask visualisation ⓘ

This section was not generated. No masks/segmentation were deposited.

PRELIMINARY VALIDATION REPORT

7 Map analysis (i)

This section contains the results of statistical analysis of the map.

7.1 Map-value distribution (i)

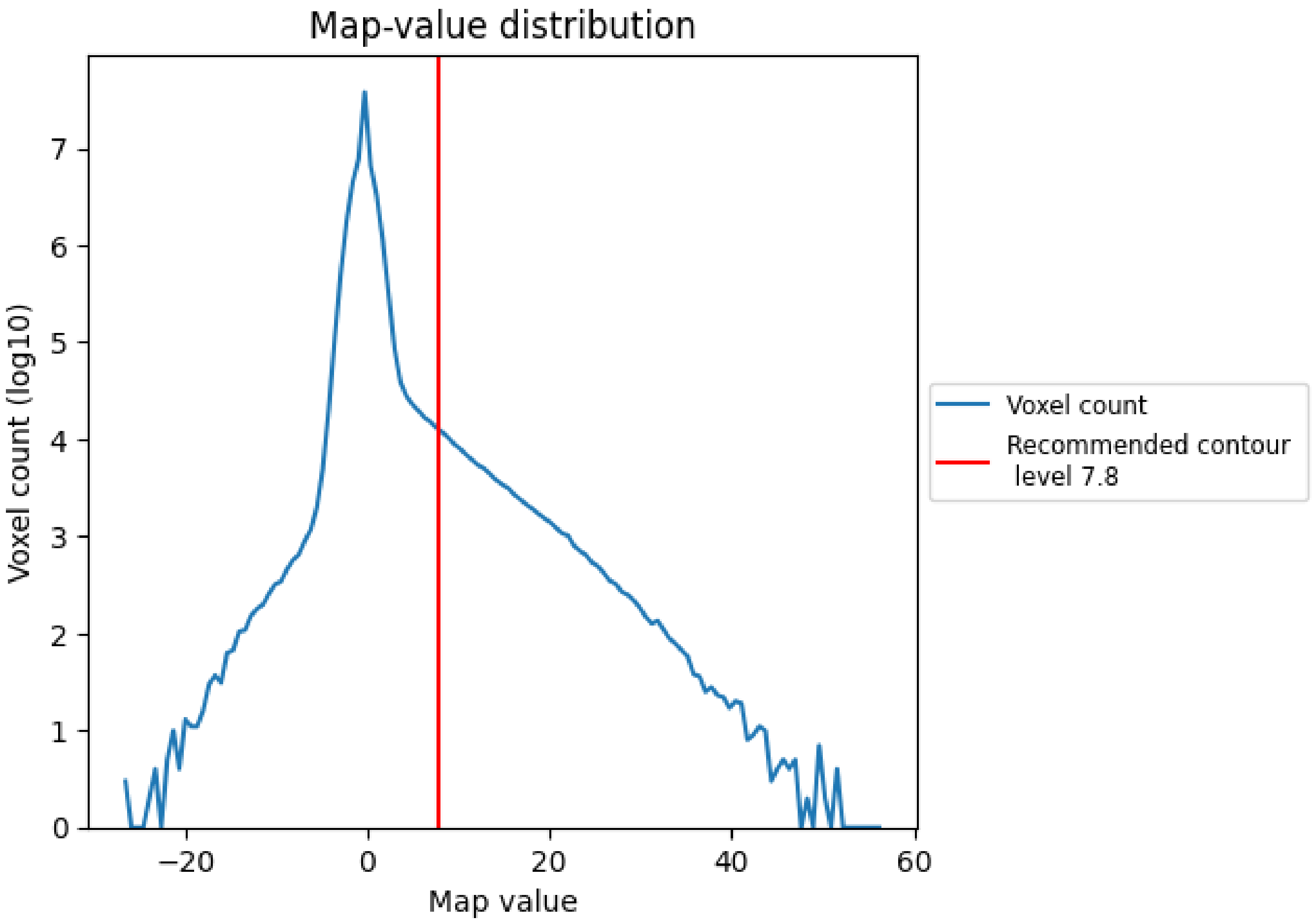

The map-value distribution is plotted in 128 intervals along the x-axis. The y-axis is logarithmic. A spike in this graph at zero usually indicates that the volume has been masked.

PRELIMINARY REPORT

7.2 Volume estimate ⓘ

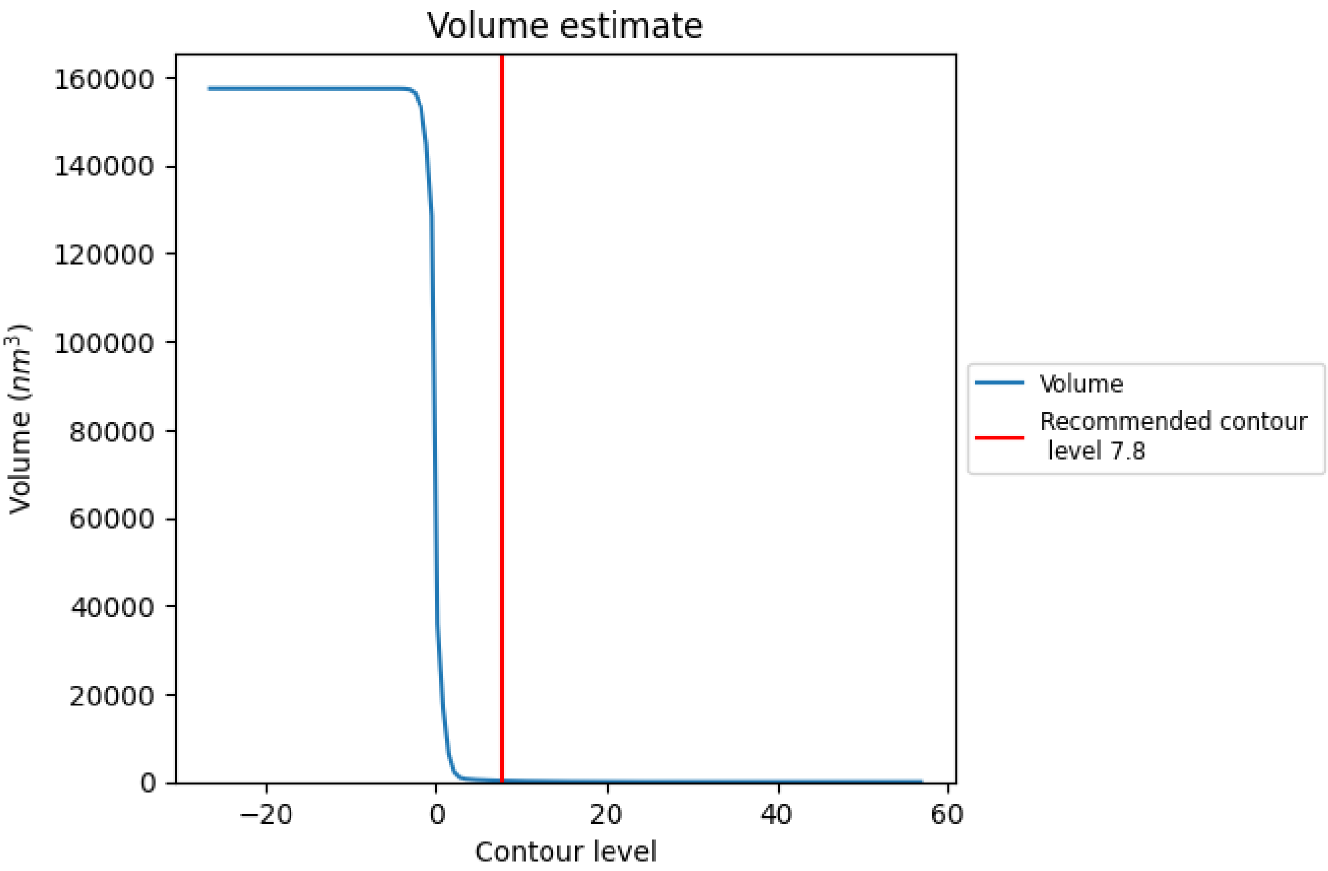

The volume at the recommended contour level is 270 nm^3; this corresponds to an approximate mass of 244 kDa.

The volume estimate graph shows how the enclosed volume varies with the contour level. The recommended contour level is shown as a vertical line and the intersection between the line and the curve gives the volume of the enclosed surface at the given level.

PRELIMINARY VA...

7.3 Rotationally averaged power spectrum (i)

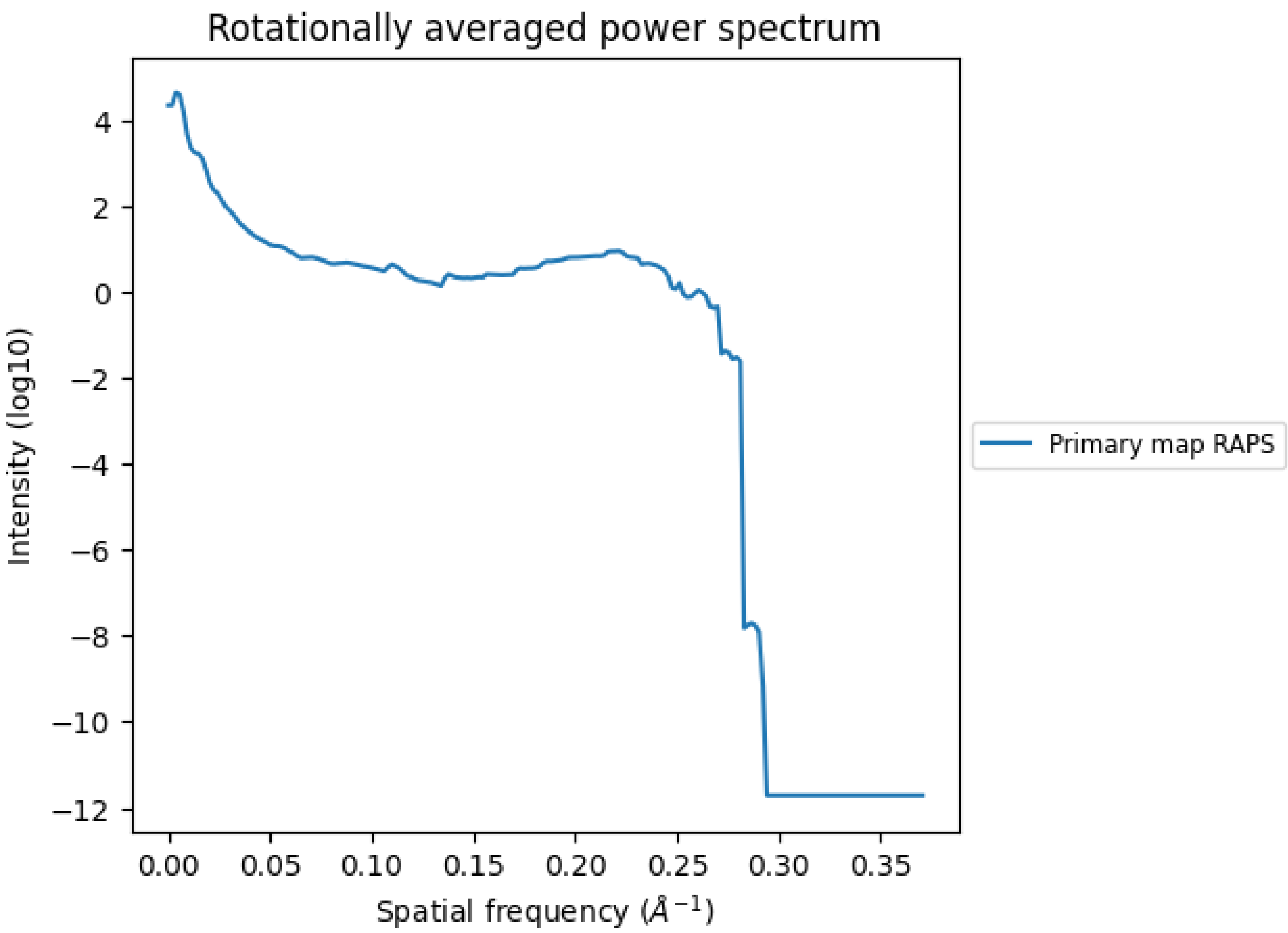

PRELIMINARY

8 Fourier-Shell correlation (i)

Fourier-Shell Correlation (FSC) is the most commonly used method to estimate the resolution of single-particle and subtomogram-averaged maps. The shape of the curve depends on the imposed symmetry, mask and whether or not the two 3D reconstructions used were processed from a common reference. The reported resolution is shown as a black line. A curve is displayed for the half-bit criterion in addition to lines showing the 0.143 gold standard cut-off and 0.5 cut-off.

8.1 FSC (i)

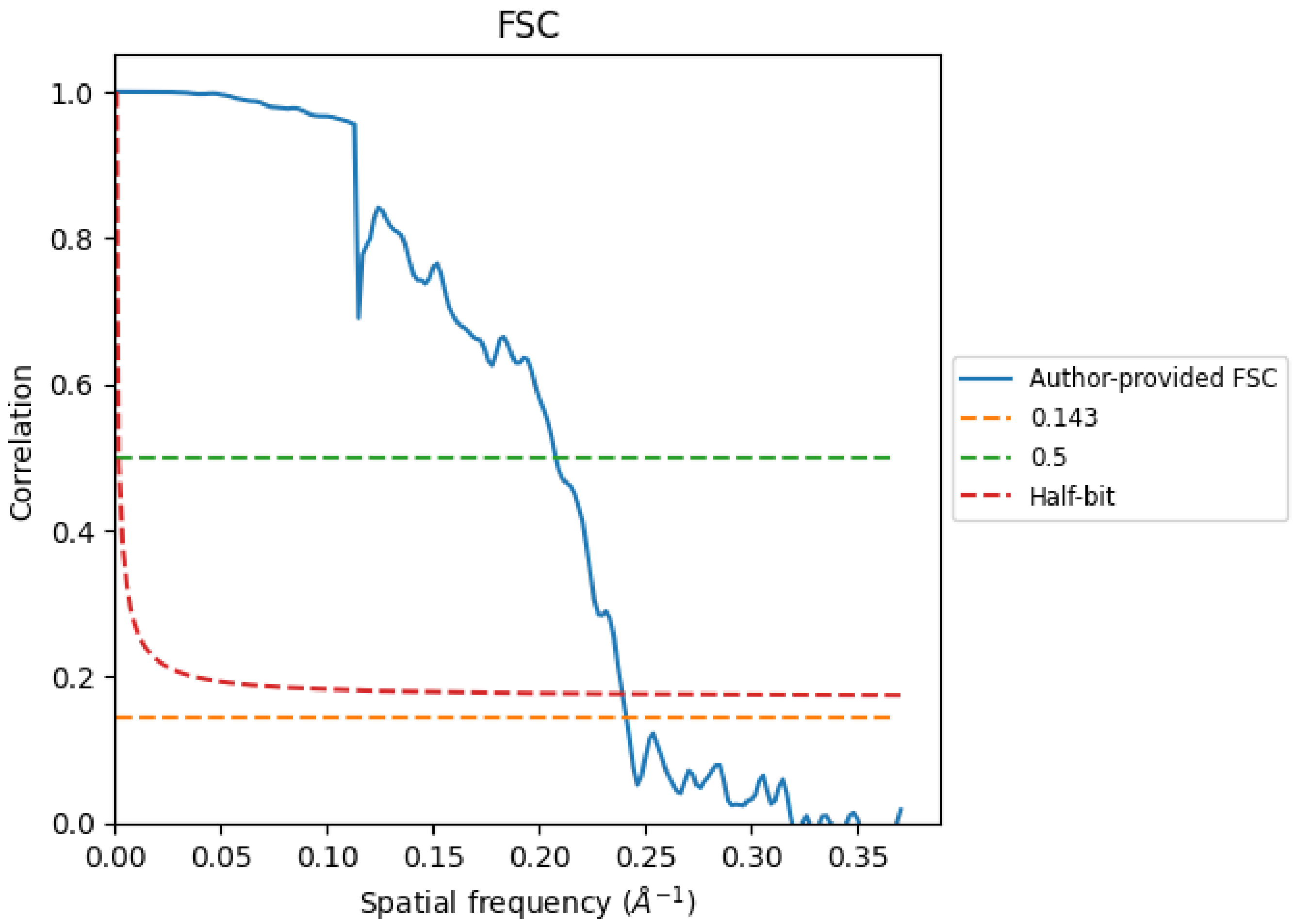

PRELIMINARY REPORT

8.2 Resolution estimates (i)

Resolution estimate (Å)	Estimation criterion (FSC cut-off)		
	0.143	0.5	Half-bit
Reported by author	-	-	-
Author-provided FSC curve	4.18	4.86	4.21
Calculated*	-	-	-

*Resolution estimate based on FSC curve calculated by comparison of deposited half-maps.

PRELIMINARY VALIDATION REPORT

9 Map-model fit (i)

This section contains information regarding the fit between EMDB map D_1000255470 and PDB model D_1000255470. Per-residue inclusion information can be found in section 3 on page 13.

9.1 Map-model overlay (i)

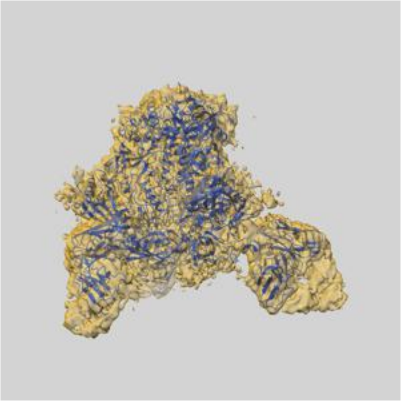

X

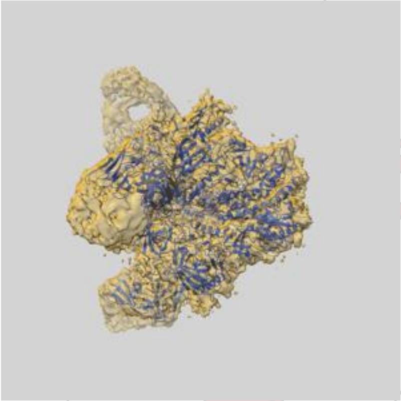

Y

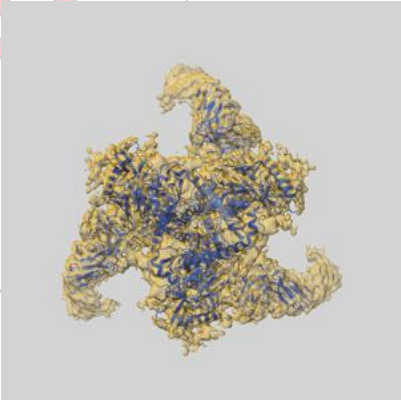

Z

The images above show the 3D surface view of the map at the recommended contour level 7.8 at 50% transparency in yellow overlaid with a ribbon representation of the model coloured in blue. These images allow for the visual assessment of the quality of fit between the atomic model and the map.

PRELIMINARY VALIDATION REPORT

9.2 Atom inclusion (i)

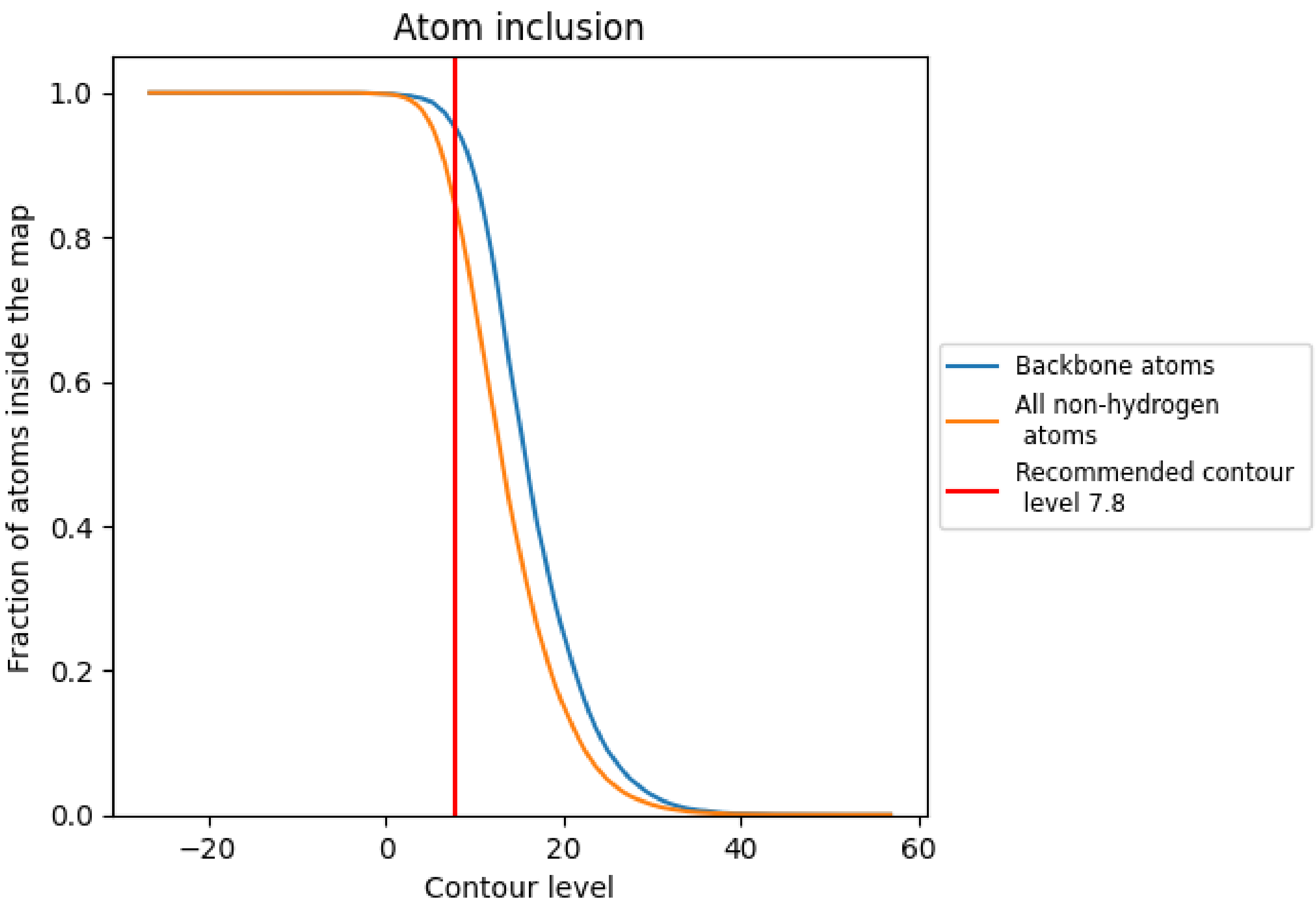

At the recommended contour level, 95% of all backbone atoms, 85% of all non-hydrogen atoms, are inside the map.

PRELIMINARY
