# Supplementary material for: Functional development of a V3/glycan-specific broadly neutralizing antibody isolated from a case of HIV superinfection
Source: eLife. 2021 Jul 15;10:e68110. doi: 10.7554/eLife.68110 (PMC8376252; doi:10.7554/eLife.68110)
Supplement: Transparent reporting form [file elife-68110-transrepform.docx]

***eLife’s* transparent reporting form**

We encourage authors to provide detailed information *within their submission* to facilitate the interpretation and replication of experiments. Authors can upload supporting documentation to indicate the use of appropriate reporting guidelines for health-related research (see [EQUATOR Network](http://www.equator-network.org/%20)), life science research (see the [BioSharing Information Resource](https://biosharing.org/)), or the [ARRIVE guidelines](http://www.plosbiology.org/article/info:doi/10.1371/journal.pbio.1000412) for reporting work involving animal research. Where applicable, authors should refer to any relevant reporting standards documents in this form.

If you have any questions, please consult our Journal Policies and/or contact us: [editorial@elifesciences.org](mailto:editorial@elifesciences.org).

**Sample-size estimation**

- You should state whether an appropriate sample size was computed when the study was being designed
- You should state the statistical method of sample size computation and any required assumptions
- If no explicit power analysis was used, you should describe how you decided what sample (replicate) size (number) to use

Please outline where this information can be found within the submission (e.g., sections or figure legends), or explain why this information doesn’t apply to your submission:

Sample-size estimation does not apply to this study that is focused on one individual. Sample size estimation is also not relevant for antibody lineage development.

**Replicates**

- You should report how often each experiment was performed
- You should include a definition of biological versus technical replication
- The data obtained should be provided and sufficient information should be provided to indicate the number of independent biological and/or technical replicates
- If you encountered any outliers, you should describe how these were handled
- Criteria for exclusion/inclusion of data should be clearly stated
- High-throughput sequence data should be uploaded before submission, with a private link for reviewers provided (these are available from both GEO and ArrayExpress)

Please outline where this information can be found within the submission (e.g., sections or figure legends), or explain why this information doesn’t apply to your submission:

Technical and biological replicate details are outlined in the Methods section for all relevant techniques – “TZM-bl Neutralization Assay”, “Biolayer Interferometry”, and “Mutational Antigenic Profiling”. Where “independent experiments” are considered technical replicates.

Replicate source data are included for all biolayer interferometry experiments and TZM-bl neutralization assays, including for the mutational antigenic profiling.

Outliers were excluded from the TZM-bl neutralization average IC_50_ calculation if the IC_50_ values deviated more than 10-fold from the rest of the technical replicates. We did not remove outlier IC_50_ values from the antigenic profiling validation experiments because the fold change in IC_50_ for each mutant was calculated relative to the wildtype pseudovirus for each technical replicate individually prior to calculating and reporting the average fold change.

Exclusion criteria for the “Sequence Analysis and Clonal Family Clustering” are described in the Methods section.

Antibody gene deep sequencing data are publicly available at BioProject SRA, accession number PRJNA674442.

**Statistical reporting**

- Statistical analysis methods should be described and justified
- Raw data should be presented in figures whenever informative to do so (typically when N per group is less than 10)
- For each experiment, you should identify the statistical tests used, exact values of N, definitions of center, methods of multiple test correction, and dispersion and precision measures (e.g., mean, median, SD, SEM, confidence intervals; and, for the major substantive results, a measure of effect size (e.g., Pearson's r, Cohen's d)
- Report exact p-values wherever possible alongside the summary statistics and 95% confidence intervals. These should be reported for all key questions and not only when the p-value is less than 0.05.

Please outline where this information can be found within the submission (e.g., sections or figure legends), or explain why this information doesn’t apply to your submission:

(For large datasets, or papers with a very large number of statistical tests, you may upload a single table file with tests, Ns, etc., with reference to sections in the manuscript.)

No statistical reporting was performed for this submission.

**Group allocation**

- Indicate how samples were allocated into experimental groups (in the case of clinical studies, please specify allocation to treatment method); if randomization was used, please also state if restricted randomization was applied
- Indicate if masking was used during group allocation, data collection and/or data analysis

Please outline where this information can be found within the submission (e.g., sections or figure legends), or explain why this information doesn’t apply to your submission:

Group allocation does not apply to this study since there were no experimental groups or clinical studies performed. Antibody lineage members (the only “groups” in this submission) were defined computationally, as described in Methods section “Sequence Analysis and Clonal Family Clustering” and “Naïve Inference and Lineage Reconstruction.”

**Additional data files (“source data”)**

- We encourage you to upload relevant additional data files, such as numerical data that are represented as a graph in a figure, or as a summary table
- Where provided, these should be in the most useful format, and they can be uploaded as “Source data” files linked to a main figure or table
- Include model definition files including the full list of parameters used
- Include code used for data analysis (e.g., R, MatLab)
- Avoid stating that data files are “available upon request”

Please indicate the figures or tables for which source data files have been provided:

Figures 2-8 source data has been uploaded for the biolayer interferometry experiments, TZM-bl neutralization assays, and cryo-EM map and coordinate PDB files.

Figure 1 and Table 1 are derived from computational analyses based on the deep sequencing data, which is a publicly available dataset with BioProject accession number PRJNA674442.
